# Iridium-Catalyzed Asymmetric Ring-Opening of Oxabenzonorbornadienes with *N*-Substituted Piperazine Nucleophiles

**DOI:** 10.3390/molecules201219748

**Published:** 2015-11-27

**Authors:** Wen Yang, Renshi Luo, Dingqiao Yang

**Affiliations:** 1 Key Laboratory of Theoretical Chemistry of Environment, Ministry of Education, School of Chemistry and Environment, South China Normal University, Guangzhou 510006, China; 18971194938@yeah.net; 2 School of Pharmaceutical Sciences, Gannan Medical University, Yi Xue Yuan Road, Ganzhou 341000, China; luorenshi2010@163.com

**Keywords:** iridium catalyst, asymmetric ring-opening, oxabenzonorbornadiene, chiral bisphosphine ligand, *N*-substituted piperazines

## Abstract

Iridium-catalyzed asymmetric ring-opening of oxabenzonorbornadienes with *N*-substituted piperazines was described. The reaction afforded the corresponding ring-opening products in high yields and moderate enantioselectivities in the presence of 2.5 mol % [Ir(COD)Cl]_2_ and 5.0 mol % (*S*)-*p*-Tol-BINAP. The effects of various chiral bidentate ligands, catalyst loading, solvent, and temperature on the yield and enantioselectivity were also investigated. A plausible mechanism was proposed to account for the formation of the corresponding *trans*-ring opened products based on the X-ray structure of product **2i**.

## 1. Introduction

The use of oxabicyclic templates to introduce *trans-*1,2-bifunctional groups to the carbocyclic molecule skeleton is an effective strategy for the synthesis of complex molecules. Pioneering work in this field was first described by Lautens and co-workers [1,2,3,4,5,6,7,8,9,10,11,12,13,14,15,16,17] who reported rhodium-catalyzed asymmetric ring-opening (ARO) of oxabicyclic alkenes to produce the corresponding products in high yields and excellent enantioselectivities (up to 99% *ee*). The asymmetric ring-opening has been extensively studied with a broad range of nucleophiles, including organomagnesium, organolithium, organozinc reagents, organoboronic acids, alcohols, phenols, carboxylic acids, terminal alkynes, and aromatic amines. In addition, many other transition metal catalysts have been tested, including Cu [18,19,20,21,22,23], Pd [24,25,26,27,28,29,30,31,32,33,34,35], Ni [36,37,38,39,40,41,42], Zr [43], Fe [44], Ru [45,46,47,48,49,50,51,52], and Pt [53,54,55] catalysts. For example, Cheng *et al*. [56,57,58,59] recently described asymmetric ring-opening of oxabenzonorbornadiene with alkyl- or alkenyl- or allylzirconium reagents and zinc powder under mild conditions catalyzed by Ni(dppe)Br_2_ or Pd((*R*)-binap)Cl_2_, which yielded the corresponding 1,2-dihydronaphth-1-ols in good to excellent yields with high enantioselcetivities (up to 90% *ee*). Carretero *et al*. [60,61] reported a general copper-catalyzed ring-opening of oxabicylic alkenes with Grignard reagents. Hou *et al*. [30] investigated the asymmetric ring-opening of oxabicyclic alkenes with arylboronic acids catalyzed by the chiral phosphine-containing palladacycle, providing corresponding products in high yields and high *ee*. On the other hand, the different kinds of ligands previously used were bisphosphine ligands, including (*S*)-BINAP, (*R*)-(*S*)-PPF-P*^t^*Bu_2_ and DPPF. Halide and triflate salts such as NH_4_F, Et_3_N∙HCl, NH_4_Br, NH_4_I, Bu_4_NI, and AgOTf were also used as additives to enhance the enantioselectivities of the ARO reaction. Recently, our group demonstrated that iridium-catalyzed asymmetric ring-opening of oxa- and azabicyclic alkenes with nitrogen- or oxygen-based nucleophiles, such as amines, alcohols, phenols and Grignard reagents [62,63,64,65,66,67,68,69,70,71,72,73,74]. Furthermore, a new iridium-monophosphine catalyst was found to be efficient for asymmetric ring-opening of benzonorbornadiene with amines, providing a series of chiral substituted dihydronaphthalenes in high yields (up to 98%) and excellent enantioselectivities (>99% *ee*) [71]. To expand the scope of this novel Ir-catalyzed reaction, we are interested in studying the asymmetric ring-opening of oxabicyclic olefins with nitrogen-based nucleophiles in the presence of an iridium catalyst. Meanwhile, we also tried to optimize the catalytic system by using additive of NH_4_I in the reaction. Herein, we reported iridium-catalyzed asymmetric ring-opening of 1,4-dihydro-1,4-epoxynaphthalene (**1a**) or 1,4-dihydro-6,7-dimethoxy-1,4-epoxynaphthalene (**1b**) with *N*-substituted piperazine nucleophiles, which afforded the corresponding ring-opening products in good yields (up to 99%) with moderate enantioselectivities. This new method also offered potentially useful synthetic routes to optically active 2-*N*-substituted piperazine 1,2-dihydronaphthalen-1-ols.

## 2. Results and Discussion

**Scheme 1:**
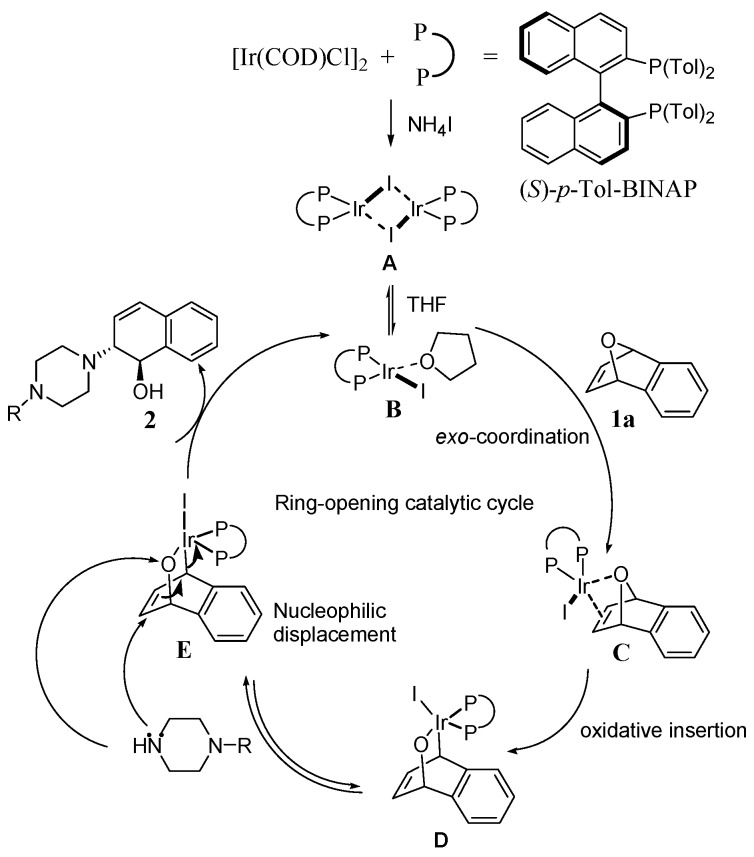
The proposed mechanism for asymmetric ring-opening of **1a** with *N*-substituted piperazines.

The substrates **1a**–**1b** were readily prepared by Diels-Alder reactions of benzynes with furan according to literature procedures [75]. To understand the nature of the catalytic ring-opening and optimize the reaction conditions, we first chose different chiral bisphosphine ligands, including (*S*)-BINAP, (*R)*-(*S*)-PPF-P*^t^^-^*Bu_2_, (*S*)-*p*-Tol-BINAP, and (*S*)-(*R*)-NMe_2_-PPh_2_-Mandyphos, to validate the catalytic activity of the iridium complexes. Consequently, a more efficient iridium catalyst system for the ring-opening reaction was explored. The different types of chiral ligands reacted with [Ir(COD)Cl]_2_ to form iridium complexes to determine the viability of the enantioselectivity (Scheme 1). To probe the iridium-catalyzed asymmetric ring-opening of oxabicyclic alkene **1a** with 1-(2-fluorophenyl)piperazine, chiral bisphosphine ligand (*S*)-*p*-Tol-BINAP was used and 1 equivalent of NH_4_I was added as the additive. We found that the ring-opening product **2a** was obtained in high yield (up to 99%) with moderate enantioselectivity (54% *ee*) (Table 1, entry 4). However, the enantiomeric excess value was low (2%–58% *ee*) when (*S*)-BINAP and ferrocene bisphosphine ligands were used as the chiral ligands (Table 1, entries 1–3). The enantiomeric excess value was 55% *ee* when (*S*)-*p*-Tol-BINAP was used as the ligand in the presence of 1.25% mol [Ir(COD)Cl]_2_ (Table 1, entry 5). Therefore, (*S*)-*p*-Tol-BINAP was chosen as the optimized ligand.

**Table 1:** Effects of chiral bisphosphine ligands and catalyst loading ^a^. 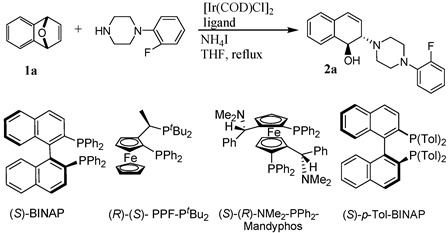

Entry	Ligand (mol %)	[Ir(COD)Cl]_2_ (mol %)	Time (h)	Yield ^b^ (%)	*ee* ^c^ (%)
1	(*S*)-BINAP(5.0)	2.5	12	76	37
2	(*R*)-(*S*)-PPF-P*^t^*^-^Bu_2_(5.0)	2.5	12	76	2
3	(*S*)-(*R*)-NMe_2_-PPh_2_-Mandyphos(5.0)	2.5	12	30	58
4	(*S*)-*p*-Tol-BINAP(5.0)	2.5	12	99	54
5	(*S*)-*p*-Tol-BINAP(2.5)	1.25	12	83	55

^a^ Conditions: [Ir(COD)Cl]_2_ (2.5 mol %) and chiral ligand (5 mol %) were dissolved in 2.0 mL THF. NH_4_I (1 equiv.) was then added and stirred for another 10–20 min. Substrate **1a** (0.3 mmol, 1 equiv.) was added and the mixture was heated to reflux. 1-(2-Fluorophenyl)piperazine (2 equiv.) was added at the first sign of reflux; ^b^ Isolated yield; ^c^ *ee* was determined by HPLC with a Chiralcel OD or AD column.

With the catalyst system consisting of [Ir(COD)Cl]_2_ and (*S*)-*p*-Tol-BINAP in hand, other reaction parameters were further optimized. We screened several commonly used solvents (Table 2, entries 1–9), the solvent effect on enantioselectivities of ring-opening reaction was remarkable, as seen from Table 2.

**Table 2:** Effects of solvent on the ring-opening ^a^. 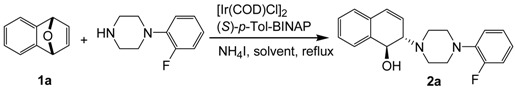

Entry	Solvent	Time (h)	Yield ^b^ (%)	*ee* ^c^ (%)
1	ClCH_2_CH_2_Cl	12	nr	--
2	DMF	12	nr	--
3	DME	12	nr	--
4	THF	12	99	54
5	THP	12	81	53
6	toluene	12	79	52
7	CH_3_CN	12	88	52
8	CH_2_Cl_2_	12	15	56
9	1,4-dioxane	12	95	50

^a^ Conditions: [Ir(COD)Cl]_2_ (2.5 mol %) and (*S*)-*p*-Tol-BINAP (5.0 mol %) were dissolved in 2.0 mL solvent. NH_4_I (1 equiv.) was then added and stirred for another 10–20 min. Substrate **1a** (0.3 mmol, 1 equiv.) was added and the mixture was heated to reflux. 1-(2-Fluorophenyl)piperazine (2 equiv.) was added at the first sign of reflux; ^b^ Isolated yield; ^c^ *ee* was determined by HPLC with a Chiralcel OD or AD column.

It was found that in 1,4-dioxane, CH_3_CN, toluene, tetrahydropyran (THP) and tetrahydrofuran (THF), the reactions were much faster than in any other solvents, and the reactions were completed in 12 h (Table 2, entries 4–7, and 9). However, the enantioselectivity was found to be 54% *ee* (Table 2, entry 4). There were no ring-opening products formed when the reactions were performed in dimethylformamide (DMF), 1,2-dimethoxyethane (DME) or 1,2-dichloroethane (Table 2, entries 1–3). Reactions in CH_2_Cl_2_ afforded the ring-opening products in a low yield (15%) with moderate enantioselectivity (56% *ee*) (Table 2, entry 8). Among several solvents examined, THF turned out to be the best, yielding the corresponding ring-opening product **2a** in 99% yield with 54% *ee*. 

The influence of temperature was also investigated in the iridium-catalyzed asymmetric ring-opening reaction of oxabicyclic alkene **1a** with 1-(2-fluorophenyl)piperazine. No product was obtained when the reaction mixture was stirred at 25 °C for 12 h (Table 3, entry 1). It was further found that the temperature had little effect on the enantioselectivity (Table 3, entries 2–4). The product **2a** was obtained in 50% yield with 40% *ee* when the reaction mixture was stirred at 50 °C for 12 h (Table 3, entry 2). Furthermore, the product **2a** was obtained in 99% yield with 54% *ee* when the reaction mixture was stirred at reflux (80 °C) (Table 3, entry 3). Consequently, the optimum reaction conditions were determined to be as follows: 2.5 mol % [Ir(COD)Cl]_2_, 5.0 mol % (*S*)-*p*-Tol-BINAP, 2 equiv. of 1-(2-fluorophenyl)piperazine, and 1 equiv. of NH_4_I to oxabicyclic alkene **1a** as additive in THF at 80 °C. 

**Table 3:** Effects of the temperature on the ring-opening ^a^. 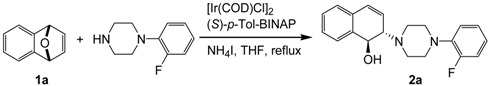

Entry	Temperature (°C)	Product	Time (h)	Yield ^b^ (%)	*ee* ^c^ (%)
1	25	**2a**	12	nr	--
2	50	**2a**	12	50	40
3	80	**2a**	12	99	54
4	100	**2a**	12	82	48

^a^ Conditions: [Ir(COD)Cl]_2_ (2.5 mol %) and (*S*)-*p*-Tol-BINAP (5.0 mol %) were dissolved in 2.0 mL THF. NH_4_I (1 equiv.) was then added and stirred for another 10–20 min. Substrate **1a** (0.3 mmol, 1 equiv.) was added and the mixture was heated. 1-(2-Fluorophenyl)piperazine (2 equiv.) was added at the first sign of reflux; ^b^ Isolated yield; ^c^ *ee* was determined by HPLC with a Chiralcel OD or AD column.

Under the optimized reaction conditions, the iridium-catalyzed ring-opening reaction of **1a** with different *N*-substituted piperazines was demonstrated to be an efficient method for the synthesis of *trans*-1,2-*N*-substituted piperazines 1,2-dihydronaphthalen-1-ols in high yields with moderate enantioselectives (Table 4). For example, various *N*-phenylpiperazines with either electron-donating or electron-withdrawing substituents at the phenyl position afforded the corresponding products in high yields (up to 99%) and good enantioselectivity in the presence of 2.5 mol % [Ir(COD)Cl]_2_ and 5.0 mol % (*S*)-*p*-Tol-BINAP (Table 4, entries 1–16, 18–19, and 22–24). 1,4-Dihydro-1,4-epoxynaphthalene (**1a**) with 1-(2-methoxyphenyl)piperazine however afforded the corresponding ring-opening product **2f** in high yield with poor enantioselectivity (Table 4, entry 6).

To further extend the scope of this transformation, the reaction of dimethoxy substituted oxabenzonorbornadiene **1b** with various *N*-substituted piperazines were also examined. It was found that the reactions of 1,4-dihydro-6,7-dimethoxy-1,4-epoxynaphthalene (**1b**), a less reactive substrate, with *N*-substituted piperazines offered the desired products in good yields with moderate enantioselectivity (Table 5, entries 1–9). 

Unfortunately, the reaction of 1,4-dihydro-6,7-dimethoxy-1,4-epoxynaphthalene (**1b**) with 1-(3,4-dichlorophenyl)piperazine afforded the corresponding ring-opening product **3e** in a lower yield (47%) with poor enantioselectivity (16% *ee*) (Table 5, entry 5). 

**Table 4:** Iridium-catalyzed asymmetric ring-opening of oxabenzonorbornadiene **1a** with *N*-substituted piperazines ^a^. 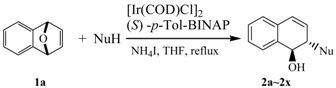

Entry	NuH	Product	Time (h)	Yield ^b^ (%)	*ee* ^c^ (%)
1	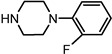	**2a**	12	99	54
2	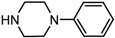	**2b**	12	87	36
3	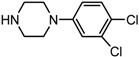	**2c**	12	86	67
4	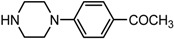	**2d**	8	87	38
5	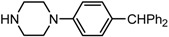	**2e**	8	98	49
6	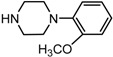	**2f**	8	81	33
7	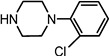	**2g**	8	89	50
8	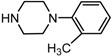	**2h**	8	87	54
9	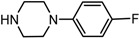	**2i**	10	91	45
10	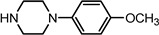	**2j**	10	85	54
11	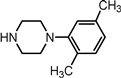	**2k**	10	98	36
12	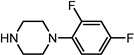	**2l**	10	97	43
13	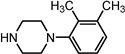	**2m**	12	88	47
14	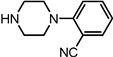	**2n**	10	95	54
15	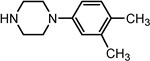	**2o**	10	96	58
16	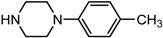	**2p**	10	85	27
17	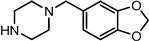	**2q**	12	86	57
18	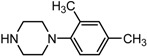	**2r**	12	89	59
19	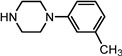	**2s**	10	90	59
20	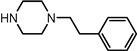	**2t**	8	78	50
21	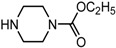	**2u**	6	85	51
22	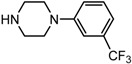	**2v**	7	83	54
23	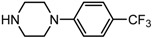	**2w**	8	76	56
24	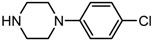	**2x**	8	82	39

^a^ Conditions: [Ir(COD)Cl]_2_ (2.5 mol %) and (*S*)-*p*-Tol-BINAP (5.0 mol %) were dissolved in 2.0 mL THF and stirred for 10–20 min. NH_4_I (1 equiv.) was then added and stirred for another 10–20 min. Substrate **1a** (0.3 mmol, 1 equiv.) was added and the mixture was heated to reflux. *N*-Substituted piperazine nucleophiles (2 equiv.) were added at the first sign of reflux; ^b^ Isolated yield; ^c^ *ee* was determined by HPLC with a Chiralcel OD or AD column.

**Table 5:** Substrate scope of the iridium-catalyzed asymmetric ring-opening reaction ^a^. 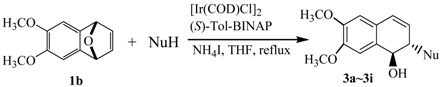

Entry	NuH	Product	Time (h)	Yield ^b^ (%)	*ee* ^c^ (%)
1	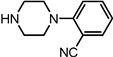	**3a**	24	77	37
2	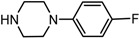	**3b**	24	73	49
3	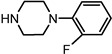	**3c**	24	79	38
4	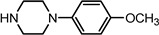	**3d**	24	62	59
5	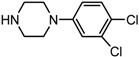	**3e**	24	47	16
6	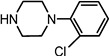	**3f**	24	51	43
7	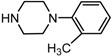	**3g**	24	76	38
8	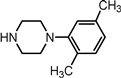	**3h**	24	86	35
9	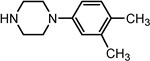	**3i**	24	81	45

^a^ Conditions: [Ir(COD)Cl]_2_ (2.5 mol %) and (*S*)-*p*-Tol-BINAP (5.0 mol %) were dissolved in 2.0 mL THF. NH_4_I (1 equiv.) was then added and stirred for another 10–20 min. Substrate **1b** (0.3 mmol, 1 equiv.) was added and the mixture was heated to reflux. *N*-Substituted piperazine nucleophiles (2 equiv.) were added at the first sign of reflux; ^b^ Isolated yield; ^c^ *ee* was determined by HPLC with a Chiralcel OD or AD column.

**Figure 1:**
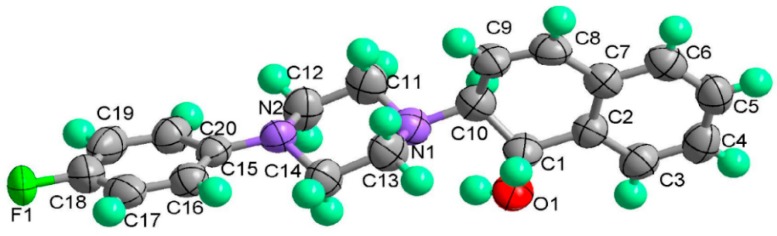
ORTEP plot for **2i** ^a^. ^a^ Crystal data. C_20_H_21_FN_2_O, *M* = 342.39. Monoclinic *a* = 9.572 (3), *b* = 10.034 (3), *c* = 17.732 (5), *alpha* = 90, *beta* = 90 (8), *gamma* = 90, *T* =296 K, space group. Orthorhombic, P 21 21 21, *Z* = 4. wR2 (reflections) = 0.1882 (3432). The Cambridge Crystallographic Data Centre (CCDC) 1415336 contains the supplementary crystallographic data for this paper. These data can be obtained free of charge via www.ccdc.cam.ac.uk/conts/retrieving.html.

The stereochemistry of 1,2*-trans* ring-opened product **2i** was unambiguously confirmed by X-ray crystallography. The single crystal of **2i** was achieved by solvent evaporation from a mixture of dichloromethane, petroleum ether and ethyl acetate. Its configuration was assigned as (1*S*, 2*S*) and confirmed as 1,2-*trans* configuration, as shown in Figure 1 (See Supplementary Materials for details). It is obvious that the ring-opening reaction favors the formation of *trans*-2-*N*-substituted piperazine 1,2-dihydro-naphthalen-1-ol products.

Based on our findings above, we propose a mechanism, outlined in Scheme 1. When [Ir(COD)Cl]_2_ was used as the iridium source, and reacted with (*S*)-*p*-Tol-BINAP to form the complex of [Ir(*S*)-*p*-Tol-BINAP)I]_2_ **A** in the presence of NH_4_I, which is then cleaved by solvent to give the monomeric iridium complex **B**. Reversible *exo* coordination of oxabenzonorbornadiene **1a** leads to iridium complex **C**, followed by oxidative insertion with retention to form a bridgehead C–O bond and produce the π-allyl iridium alkoxide complex **D**. We further propose that the oxidative cleavage of the C–O bond is the enantioselectivity discriminating step in the catalytic cycle. Once iridium complex **C** is formed, the iridium alkoxide complex could be protonated by the *N*-substituted piperazine nucleophiles to generate cationic iridium complex **E**. This proton transfer has two effects. First, the iridium species are made more electrophilic as a result of the positive charge, and the nucleophile is rendered more nucleophilic by deprotonation. Second, the positioning of the iridium metal on the π-allyl moiety will influence the regioselectivity of nucleophilic attack. Nucleophilic attack with inversion is proposed to occur adjacent to the alkoxy group in an S_N_2 fashion relative to the iridium metal. Finally, product **2** is subsequently liberated and the iridium monomer is regenerated, which will either reform the dimer or continue the catalytic cycle.

## 3. Experimental Section

### 3.1. Chemistry

#### 3.1.1. General

Solvents and solutions were transferred with syringes. ^1^H-NMR spectra were recorded at 400 MHz using a Varian XL (Palo Alto, CA, USA) 400 spectrometer with CDCl_3_ as reference standard (7.27 ppm). Spectral features are tabulated in the following order: Chemical shift (ppm); number of protons; multiplicity (s—singlet, d—doublet, t—triplet, q—quartet, m—multiplet, br—broad); coupling constants (*J*, Hz), ^13^C-NMR spectra were recorded at 400 MHz with CDCl_3_ as reference standard (77.23 ppm). IR spectra were obtained using a Nicolet DX (Madison, WI, USA) FT-IR spectrometer. High resolution mass was obtained from a VG 70-250S (double focusing) mass spectrometer at 70 ev (Waters, Milford, MA, USA). The enantiomeric excess value was measured by HPLC with CHIRALCEL OD or AD columns (Chiral Technologies, Minato-ku, Japan). Melting points were taken with a Tai-Ke melting point apparatus (Beijing, China). Analytical TLC was performed using EM separations percolated silica gel 0.2 mm layer UV 254 nm fluorescent sheets (Beijing, China). Column chromatography was performed as “Flash chromatography” as reported by using (200–300 mesh) Merck grade silica gel (Merck, Beijing, China). The THF, toluene, DME, and THP was distilled from sodium benzophenone ketyl immediately prior to use. DMF, CH_2_Cl_2_, CH_3_CN, ClCH_2_CH_2_Cl, and 1,4-dioxane were distilled from calcium hydride. Furan was distilled prior to use. All other reagents were obtained from Alfa Aesar (Shanghai, China) and *J* & K (Guangzhou, China) and used as received unless otherwise stated.

#### 3.1.2. Preparation of 1,4-Dihydro-1,4-epoxynaphthalene (**1a**) 

To a 100 mL round-bottomed flask with a reflux condenser tube, 10 mL furan and 10 mL DME were added. Taking two 25 mL dropping bottles, one with 4 mL iso-amyl nitrite and 10 mL DME (A), another with 2-aminobenzoic acid (2.75 g, 0.02 mol) dissolved by 10 mL DME (B). Then 1 mL A and 1 mL B were added to the refluxing furan solution per 4 minute. Firstly, the A was added, then the B. The solution became red brown, giving off gas when the reagents were added. Let the mixture refluxing until the solution did not release gas after all the reactants were added (about 15 min). After completion 2% sodium hydroxide (25 mL) was added to the mixture and transferred to separating funnel to rinse, which we can get the organic phase and the aqueous solution extracted three times by 15 mL petroleum ether (bp. 30–60 °C). Then the extractive solution and the organic phase were mixed together. The mixture was washed by water (15 mL × 4) and dried by anhydrous magnesium sulfate. After completion the reaction mixture was concentrated in *vacuo* and the solvents were removed, the crude mixture was purified by flash chromatography gave **1a** a yellow solid (1.72 g, 60%). R_f_ = 0.45 on silica gel (25% ethyl acetate in petroleum ether). m.p.: 55–56 °C. IR (thin film, cm^−1^) 3125 (s), 3040 (s), 3020 (s), 1958 (s), 1916 (s), 1814 (s), 1620 (s), 1562 (s), 1449 (s), 1345 (s), 1278 (s), 1195 (s), 1164 (s), 1128 (s), 1073 (s), 986 (s), 938 (s), 844 (s), 765 (s), 689 (s), 635 (s). ^1^H-NMR (400 MHz, CDCl_3_) δ 7.36–7.20 (m, 1H), 7.05 (t, *J* = 1.0 Hz, 1H), 7.02–6.94 (m, 1H), 5.74 (s, 1H). ^13^C-NMR (100 MHz, CDCl_3_) δ 149.0, 143.0, 125.0, 120.3, 82.3.

#### 3.1.3. Preparation of 1,4-Dihydro-6,7-dimethoxy-1,4-epoxynaphthalene (**1b**)

A stirred solution of 1,2-dibromo-4,5-dimethoxybenzene (2.0 g, 6.8 mmol) and furan (20 mL) in anhydrous THF (20 mL) was maintained at −78 °C under N_2_ and was treated dropwise with *n*-butyl lithium (1.29 mol/L) in hexane (5.3 mL). The solution was stirred at −78 °C for 0.5 h and then allowed to room temperature during 2 h. The work-up yielded a crude product was purified by flash chromatography (30% ethyl acetate in hexanes) to give **1b** a white solid (0.9 g, 65%). R_f_ = 0.21 on silica gel (30% ethyl acetate in hexanes); m.p.: 128–130 °C; IR (thin film, cm^−1^) 2926 (s), 2924 (s), 2839 (s), 1599 (s), 1485 (s), 1467 (s), 1325 (s), 1285 (s), 1063 (s); ^1^H-NMR (400 MHz, CDCl_3_) δ 7.04 (2H, t, *J* = 0.91 Hz, Ar-H), 6.97 (2H, s, =CH), 5.68 (2H, s, CH), 3.85 (6H, s, CH_3_O); ^13^C-NMR (100 MHz, CDCl_3_) δ 146.1, 143.6 141.9, 107.0, 82.8, 56.7.

#### 3.1.4. General Procedure for the Asymmetric Ring-Opening of **1a** with *N*-Substituted Piperazines

A 5.0 mL round-bottomed flask was equipped with a reflux condenser, 2.5 mol % chloro(1,5-cyclooctadiene)iridium (I) dimer [Ir(COD)Cl]_2_ and 5.0 mol % (*S*)-*p*-Tol-BINAP were added and followed by addition of anhydrous tetrahydrofuran (2.0 mL). After they were stirred for 10 min to produce a yellow solution. 1,4-Dihydro-1,4-epoxynaphthalene **1a** (50 mg, 0.3468 mmol) was added; then 10 min later, additive of ammonium iodide (1.0 equiv. to **1a**) was added and heated to reflux. At the first sign of reflux, *N*-substituted piperazine nucleophiles (2.0 equiv. to **1a**) were added. The reaction mixture was stirred at reflux and monitored by TLC until completion (typically 6–12 h). The solvent was removed in *vacuo* and the crude mixture was purified by column chromatography on silica gel to afford the desired products.

*(1S,2S)-2-[4-(2-Fluoro-phenyl)-piperazin-1-yl]-1,2-dihydro-naphthalen-1-ol* (**2a**). Prepared according to general procedure. **2a** was obtained as a white solid (111.9 mg, 99%) by flash chromatography (ethyl acetate: petroleum ether = 1:4, *v*/*v*). R_f_ = 0.21 on silica gel (ethyl acetate: petroleum ether = 1:4, *v*/*v*). The *ee* was determined to be 54% using HPLC analysis on a CHIRALCEL AD column, λ = 254 nm. Flow rate = 0.5 mL/min; Retention times in 10% 2-propanol in hexanes were 19.0 min (major) and 20.9 min (minor). m.p.: 125–126 °C; ${\left[\alpha \right]}_{D}^{25}$ = +83.2° (*c* = 68.9 mg, CHCl_3_); IR (thin film, cm^−1^) 3510 (br), 3054 (w), 2977 (s), 2934 (s), 2862 (s), 1490 (s), 1445 (s), 1383 (s), 1351 (s), 1077 (s), 846 (s); ^1^H-NMR (400 MHz, CDCl_3_) δ 7.60 (1H, d, *J* = 7.2 Hz), 7.31–7.23 (2H, m), 7.11–6.93 (5H, m), 6.58 (1H, dd, *J* = 2.4 Hz, *J* = 2.4 Hz), 6.17 (1H, dd, *J* = 2.4 Hz, *J* = 2.4 Hz), 4.93 (1H, 11.6), 3.53 (1H, dt, *J* = 11.6 Hz, *J* = 2.4 Hz), 3.22 (1H, br), 3.21–3.05 (4H, m), 3.02–2.98 (2H, m), 2.78–2.73 (2H, m); ^13^C-NMR (100 MHz, CDCl_3_) δ 157.1, 154.7, 140.2, 137.2, 131.9, 129.6, 128.1, 127.6, 126.4, 124.8, 124.7, 122.8, 119.2, 116.4, 67.9, 67.7, 51.3, 49.2. MS (ESI): calcd *m*/*z* for C_20_H_21_FN_2_O (M^+^) 324.16, found: 325.12 [M + H]^+^. Anal. Calcd for C_20_H_21_FN_2_O: C, 74.05; H, 6.53; N, 8.64. Found: C, 74.29; H, 6.74; N, 8.56.

*(1S,2S)-2-(4-Phenyl-piperazin-1-yl)-1,2-dihydro-naphthalen-1-ol* (**2b**). Prepared according to general procedure. **2b** was obtained as a white solid (93 mg, 87%) by flash chromatography (ethyl acetate: petroleum ether = 1:1, *v*/*v*). R_f_ = 0.42 on silica gel (ethyl acetate: petroleum ether = 1:1, *v*/*v*). The *ee* was determined to be 36% using HPLC analysis on a CHIRALCEL OD column, λ = 254 nm. Flow rate = 0.5 mL/min; Retention times in 2% 2-propanol in hexanes were 31.3 min (minor) and 35.9 min (major). m.p.: 162–163 °C; ${\left[\alpha \right]}_{D}^{25}$ = + 62.8° (*c* = 39.5 mg, CHCl_3_); IR (thin film, cm^−1^) 3345 (br), 3016 (w), 2928 (s), 2824 (s), 1597 (m), 1492 (s), 1452 (s), 1369 (m), 1226 (s), 1169 (s), 1133 (m), 1045 (m), 763 (s); ^1^H-NMR (400 MHz, CDCl_3_) δ 7.56 (1H, d, *J* = 7.2 Hz), 7.26–7.19 (4H, m), 7.05 (1H, dd, *J* = 4.2 Hz, *J* = 2.8 Hz), 6.91–6.82 (3H, m), 6.52 (1H, dd, *J* = 2.8 Hz, *J* = 2.8 Hz), 6.08 (1H, dd, *J* = 2.4 Hz, *J* = 2.4 Hz), 4.89 (1H, *J* = 7.6 Hz), 3.50 (1H, *J* = 2.8 Hz), 3.37 (1H, br), 3.23–3.13 (4H, m), 2.95–2.89 (2H, m), 2.70–2.65 (2H, m); ^13^C-NMR (100 MHz, CDCl_3_) δ 151.4, 137.2, 131.9, 129.6, 129.3, 128.1, 127.6, 126.4, 125.0, 124.5, 120.1, 116.4, 67.9, 67.6, 49.9, 49.1. MS (ESI): calcd *m*/*z* for C_20_H_22_N_2_O (M^+^) 306.17, found: 307.16 [M + H]^+^. Anal. Calcd for C_20_H_22_N_2_O: C, 78.40; H, 7.24; N, 9.14. Found: C, 78.21; H, 7.31; N, 9.28.

*(1S,2S)-2-[4-(3,4-Dichloro-phenyl)-piperazin-1-yl]-1,2-dihydro-naphthalen-1-ol* (**2c**). Prepared according to general procedure. **2c** was obtained as a white solid (112 mg, 86%) by flash chromatography (ethyl acetate: petroleum ether = 1:1, *v*/*v*). R_f_ = 0.37 on silica gel (ethyl acetate: petroleum ether = 1:1, *v*/*v*). The *ee* was determined to be 67% using HPLC analysis on a CHIRALCEL. AD column, λ = 254 nm. Flow rate = 0.5 mL/min; Retention times in 10% 2-propanol in hexanes were 33.9 min (major) and 36.9 min (minor). m.p.: 142–143 °C; ${\left[\alpha \right]}_{D}^{25}$ = +100.4° (*c* = 45.6 mg, CHCl_3_); IR (thin film, cm^−1^) 3456 (br), 3027 (w), 2936 (m), 2836 (s), 1594 (s), 1552 (m), 1483 (s), 1452 (m), 1237 (s), 1139 (m), 1044 (s), 782 (s); ^1^H-NMR (400 MHz, CDCl_3_) δ 7.51 (1H, d, *J* = 7.2 Hz), 7.30–7.20 (3H, m), 7.05 (1H, d, *J* = 6.8 Hz), 6.91 (1H, d, *J* = 2.8 Hz), 6.72 (1H, dd, *J* = 2.4 Hz, *J* = 2.8 Hz), 6.46 (1H, dd, *J* = 2.0 Hz, *J* = 1.6 Hz), 6.02 (1H, dd, *J* = 2.4 Hz, *J* = 2.4 Hz), 4.83 (1H, d, *J* = 11.6 Hz), 3.42 (1H, d, *J* = 10.8 Hz), 3.22–3.05 (5H, m), 2.98–2.85 (2H, m), 2.70–2.61 (2H, m); ^13^C-NMR (100 MHz, CDCl_3_) δ 150.7, 137.0, 132.9, 131.8, 130.6, 129.7, 128.1, 127.7, 126.4, 125.1, 124.3, 122.4, 117.5, 115.6, 67.9, 67.5, 49.4, 48.8. MS (ESI): calcd *m*/*z* for C_20_H_20_Cl_2_N_2_O (M^+^) 374.10, found: 375.05 [M + H]^+^. Anal. Calcd for C_20_H_20_Cl_2_N_2_O: C, 64.01; H, 5.37; N, 7.46. Found: C, 63.82; H, 5.69; N, 7.47.

*(1S,2S)-1-{4-(1-Hydroxy-1,2-dihydro-naphthalen-2-yl)-piperazin-1-yl]-phen-yl}-ethanone* (**2d**). Prepared according to general procedure. **2d** was obtained as a white solid (105 mg, 87%) by flash chromatography (ethyl acetate: petroleum ether = 2:1, *v*/*v*). R_f_ = 0.40 on silica gel (ethyl acetate: petroleum ether = 2:1, *v*/*v*). The *ee* was determined to be 38% using HPLC analysis on a CHIRALCEL OD column, λ = 254 nm. Flow rate = 0.5 mL/min; Retention times in 2% 2-propanol in hexanes were 30.3 min (minor) and 34.9 min (major). m.p.: 202–203 °C; ${\left[\alpha \right]}_{D}^{25}$ = −3.0° (*c* = 26.3 mg, CHCl_3_); IR (thin film, cm^−1^) 3396 (br), 3018 (w), 2923 (s), 2841 (s), 1739 (m), 1643 (s), 1597 (s), 1388 (s), 1245 (s), 1086 (s), 817 (s); ^1^H-NMR (400 MHz, CDCl_3_) δ 7.88 (2H, d, *J* = 8.8 Hz), 7.58 (1H, d, *J* = 6.8 Hz), 7.27 (2H, td, *J* = 7.6 Hz, *J* = 7.2 Hz), 7.10 (1H, d, *J* = 6.8 Hz), 6.88 (2H, d, *J* = 7.6 Hz), 6.57 (1H, d, *J* = 6.0 Hz), 6.08 (1H, d, *J* = 9.6 Hz), 4.93 (1H, d, *J* = 11.2 Hz), 3.54 (1H, d, *J* = 2.0 Hz), 3.41–3.35 (4H, m), 3.19 (1H, br), 2.97–2.94 (2H, m), 2.73–2.71 (2H, m), 2.52 (3H, s); ^13^C-NMR (100 MHz, CDCl_3_) δ 196.7, 154.3, 137.0, 131.9, 130.6, 129.8, 128.2, 128.0, 127.8, 126.5, 125.2, 124.3, 113.7, 68.0, 67.6, 48.9, 48.1, 26.3. MS (ESI): calcd *m*/*z* for C_22_H_24_N_2_O_2_ (M^+^) 348.18, found: 349.15 [M + H]^+^. Anal. Calcd for C_22_H_24_N_2_O_2_: C, 75.83; H, 6.94; N, 8.04. Found: C, 75.65; H, 7.34; N, 8.01.

*(1S,2S)-2-(4-Benzhydryl-piperazin-1-yl)-1,2-dihydro-naphthalen-1-ol* (**2e**). Prepared according to general procedure. **2e** was obtained as a white solid (135 mg, 98%) by flash chromatography (ethyl acetate: petroleum ether = 1:4, *v*/*v*). R_f_ = 0.22 on silica gel (ethyl acetate: petroleum ether = 1:4, *v*/*v*). The *ee* was determined to be 49% using HPLC analysis on a CHIRALCEL OD column, λ = 254 nm. Flow rate = 0.5 mL/min; Retention times in 2% 2-propanol in hexanes were 18.2 min (major) and 19.8 min (minor). m.p.: 160–161 °C; ${\left[\alpha \right]}_{D}^{25}$ = +150.2° (*c* = 33.3 mg, CHCl_3_); IR (thin film, cm^−1^) 3429 (br), 3027 (w), 2923 (s), 2807 (m), 1594 (s), 1487 (m), 1451 (s), 1383 (m), 1133 (s), 1040 (s), 741 (s), 697 (s); ^1^H-NMR (400 MHz, CDCl_3_) δ 7.66 (1H, d, *J =* 7.2 Hz), 7.52 (4H, d, *J =* 7.6 Hz), 7.39–7.25 (8H, m), 7.15 (1H, d, *J =* 7.2 Hz), 6.62 (1H, dd, *J =* 2.4 Hz, *J =* 2.4 Hz), 6.26 (1H, dd, *J =* 2.4 Hz, *J =* 2.4 Hz), 4.94 (1H, *J =* 11.6 Hz), 4.34 (1H, s), 3.53 (1H, dt, *J =* 11.6 Hz, *J =* 2.4 Hz), 3.45 (1H, br), 2.98–2.84 (2H, m), 2.69–2.61 (2H, m), 2.49–2.42 (4H, m); ^13^C-NMR (100 MHz, CDCl_3_) δ 142.8, 137.3, 132.0, 129.3, 128.7, 128.5, 128.1, 127.9, 127.5, 127.1, 126.3, 125.0, 124.9, 67.8, 67.3, 52.5, 49.2. MS (ESI): calcd *m*/*z* for C_27_H_28_N_2_O (M^+^) 396.22, found: 397.19 [M + H]^+^. Anal. Calcd for C_27_H_28_N_2_O: C, 81.87; H, 7.12; N, 7.06. Found: C, 81.52; H, 7.43; N, 7.02.

*(1S,2S)-2-[4-(2-Methoxy-phenyl)-piperazin-1-yl]-1,2-dihydro-naphthalen-1-ol* (2f)*.* Prepared according to general procedure. 2f was obtained as a white solid (95 mg, 81%) by flash chromatography (ethyl acetate: petroleum ether = 1:4, *v*/*v*). R_f_ = 0.17 on silica gel (ethyl acetate: petroleum ether = 1:4, *v*/*v*); The *ee* was determined to be 33% using HPLC analysis on a CHIRALCEL OD column, λ = 254 nm. Flow rate = 0.5 mL/min; Retention times in 2% 2-propanol in hexanes were 27.6 min (minor) and 29.3 min (major); m.p.: 148–149 °C; ${\left[\alpha \right]}_{D}^{25}$ = +53.8° (*c* = 69.9 mg, CHCl_3_); IR (thin film, cm^−1^) 3519 (br), 3089 (w), 2978 (s), 2934 (s), 2863 (s), 2805 (s), 1490 (s), 1445 (s), 1415 (s), 1383 (s), 1351 (s), 1297 (m), 1076 (s), 1044 (s), 935 (s), 846 (s); ^1^H-NMR (400 MHz, CDCl_3_) δ 7.61 (1H, d, *J* = 7.2 Hz), 7.29–7.25 (2H, m), 7.10 (1H, d, *J* = 7.2 Hz), 7.02 (1H, dt, *J* = 8.0 Hz, *J* = 2.4 Hz), 6.98–6.95 (2H, m), 6.88 (1H, d, *J* = 8.0 Hz), 6.57 (1H, dd, *J* = 2.4 Hz, *J* = 2.4 Hz), 6.20 (1H, dd, *J* = 2.4 Hz, *J* = 2.4 Hz), 4.95 (1H, d, *J* = 11.6 Hz), 3.88 (3H, s), 3.54 (1H, *J* = 6.4 Hz), 3.41 (1H, br), 3.18–3.01 (4H, m), 3.01–2.98 (2H, m), 2.79–2.70 (2H, m); ^13^C-NMR (100 MHz, CDCl_3_) δ 152.4, 141.3, 137.3, 132.0, 129.4, 128.0, 127.6, 126.3, 125.0, 124.9, 123.2, 121.1, 118.4, 111.2, 67.8, 67.7, 55.5, 51.4, 49.3. MS (ESI): calcd *m*/*z* for C_21_H_24_N_2_O_3_ (M^+^) 336.18, found: 337.10 [M + H]^+^. Anal. Calcd for C_21_H_24_N_2_O_3_: C, 74.97; H, 7.19; N, 8.33. Found: C, 74.89; H, 7.44; N, 8.56.

*(1S,2S)-2-[4-(2-Chloro-phenyl)-piperazin-1-yl]-1,2-dihydro-naphthalen-1-ol* (**2g**). Prepared according to general procedure. **2g** was obtained as a white solid (105 mg, 89%) by flash chromatography (ethyl acetate: petroleum ether = 1:3, *v*/*v*). R_f_ = 0.25 on silica gel (ethyl acetate: petroleum ether = 1:3, *v*/*v*). The *ee* was determined to be 50% using HPLC analysis on a CHIRALCEL AD column, λ = 254 nm. Flow rate = 0.5 mL/min; Retention times in 10% 2-propanol in hexanes were 19.4 min (major) and 20.3 min (minor). m.p.: 122–123 °C; ${\left[\alpha \right]}_{D}^{25}$ = +91.6° (*c* = 29.7 mg, CHCl_3_); IR (thin film, cm^−1^) 3468 (br), 3060 (w), 2927 (s), 2828 (s), 1588 (s), 1480 (s), 1453 (s), 1377 (m), 1230 (s), 1123 (s), 1040 (s), 781 (s), 749 (s); ^1^H-NMR (400 MHz, CDCl_3_) δ 7.61 (1H, d, *J* = 7.2 Hz), 7.37 (1H, d, *J* = 7.2 Hz), 7.31–7.22 (3H, m), 7.11–6.99 (2H, m), 6.97 (1H, d, *J =* 8.4 Hz), 6.58 (1H, dd, *J =* 2.8 Hz, *J =* 2.8 Hz), 6.21 (1H, dd, *J =* 2.8 Hz, *J =* 2.4 Hz), 4.95 (1H, 11.6), 3.53 (1H, dt, *J =* 11.6 Hz, *J =* 2.4 Hz), 3.34 (1H, br), 3.15–3.09 (4H, m), 3.05–2.95 (2H, m), 2.79–2.74 (2H, m); ^13^C-NMR (100 MHz, CDCl_3_) δ 149.3, 137.3, 132.0, 130.9, 129.6, 129.0, 128.1, 127.8, 127.6, 126.4, 124.9, 124.0, 120.6, 67.9, 67.8, 51.9, 49.4. MS (ESI): calcd *m*/*z* for C_20_H_21_ClN_2_O (M^+^) 340.13, found: 341.11 [M + H]^+^. Anal.Calcd for C_20_H_21_ClN_2_O: C, 70.48; H, 6.21; N, 8.22. Found: C, 70.20; H, 6.49; N, 8.49.

*(1S,2S)-2-[4-o-Toyl-piperazin-1-yl]-1,2-dihydro-naphthalen-1-ol* (**2h**). Prepared according to general procedure. **2h** was obtained as a white solid (97 mg, 87%) by flash chromatography (ethyl acetate: petroleum ether = 1:3, *v*/*v*). R_f_ = 0.2 on silica gel (ethyl acetate: petroleum ether = 1:3, *v*/*v*). The *ee* was determined to be 54% using HPLC analysis on a CHIRALCEL AD column, λ = 254 nm. Flow rate = 0.5 mL/min; Retention times in 10% 2-propanol in hexanes were 14.7 min (major) and 16.4 min (minor). m.p.: 115–116 °C; ${\left[\alpha \right]}_{D}^{25}$ = +95.9° (*c* = 49.1 mg, CHCl_3_); IR (thin film, cm^−1^) 3461 (br), 3019 (m), 2949 (s), 2878 (s), 2816 (w), 1596 (w), 1490 (s), 1453 (s), 1256 (m), 1224 (s), 1195 (m), 1132 (m), 1049 (s), 781 (s), 768 (s); ^1^H-NMR (400 MHz, CDCl_3_) δ 7.69 (1H, d, *J =* 7.2 Hz), 7.38–7.25 (4H, m), 7.19–7.07 (3H, m), 6.66 (1H, dd, *J =* 2.4 Hz, *J =* 2.4 Hz), 6.29 (1H, dd, *J =* 2.8 Hz, *J =* 2.4 Hz), 5.03 (1H, *J =* 12.0 Hz), 3.60 (1H, dt, *J =* 12.0 Hz, *J =* 2.8 Hz), 3.39 (1H, br), 3.11–3.02 (6H, m), 2.83–2.79 (2H, m), 2.42 (3H, s); ^13^C-NMR (100 MHz, CDCl_3_) δ 151.6, 137.4, 132.8, 132.0, 131.3, 129.5, 128.1, 127.6, 126.3, 125.0, 124.9, 123.5, 119.2, 67.9, 67.8, 52.5, 49.6, 18.1. MS (ESI): calcd *m*/*z* for C_21_H_24_N_2_O (M^+^) 320.19, found: 321.16 [M + H]^+^. Anal. Calcd for C_21_H_24_N_2_O: C, 78.71; H, 7.55; N, 8.74. Found: C, 78.49; H, 7.82; N, 8.69.

*(1S,2S)-2-[4-(4-Fluoro-phenyl)-piperazin-1-yl]-1,2-dihydro-naphthalen-1-ol* (**2i**). Prepared according to general procedure. **2i** was obtained as a white solid (101 mg, 91%) by flash chromatography (ethyl acetate: petroleum ether = 1:2, *v*/*v*). R_f_ = 0.34 on silica gel (ethyl acetate: petroleum ether = 1:2, *v*/*v*). The *ee* was determined to be 45% using HPLC analysis on a CHIRALCEL AD column, λ = 254 nm. Flow rate = 0.5 mL/min; Retention times in 10% 2-propanol in hexanes were 26.9 min (major) and 29.4 min (minor). m.p.: 167–168 °C; ${\left[\alpha \right]}_{D}^{25}$ = +133.2° (*c* = 48.2 mg, CHCl_3_); IR (thin film, cm^−1^) 3592 (br), 3214 (w), 2978 (s), 2934 (s), 2872 (s), 2806 (s), 1627 (w), 1489 (s), 1445 (s), 1415 (s), 1298 (s), 1067 (s), 1044 (s), 935 (m), 846 (s); ^1^H-NMR (400 MHz, CDCl_3_) δ 7.59 (1H, d, *J =* 7.2 Hz), 7.29–7.25 (2H, m), 7.09 (1H, d, *J =* 7.2 Hz), 7.00–6.96 (2H, m), 6.91–6.87 (2H, m), 6.57 (1H, dd, *J =* 2.8 Hz, *J =* 2.4 Hz), 6.13 (1H, dd, *J =* 2.4 Hz, *J =* 2.4 Hz), 4.93 (1H, d, *J =* 11.6 Hz), 3.53 (1H, dt, *J =* 11.6 Hz, *J =* 2.8 Hz), 3.27 (1H, br), 3.18–3.13 (4H, m), 3.00–2.95 (2H, m), 2.75–2.71 (2H, m); ^13^C-NMR (100 MHz, CDCl_3_) δ 158.7, 156.3, 148.1, 137.2, 131.9, 129.7, 128.1, 127.7, 126.4, 125.0, 125.0, 118.2, 115.9, 67.9, 67.6, 51.0, 49.1. MS (ESI): calcd *m*/*z* for C_20_H_21_FN_2_O (M^+^) 324.16, found: 325.15 [M + H]^+^. Anal. Calcd for C_20_H_21_FN_2_O: C, 74.05; H, 6.53; N, 8.64. Found: C, 74.19; H, 6.74; N, 8.66.

*(1S,2S)-2-[4-(4-Methoxy-phenyl)-piperazin-1-yl]-1,2-dihydro-naphthalen-1-ol* (**2j**). Prepared according to general procedure. **2j** was obtained as a white solid (99 mg, 85%) by flash chromatography (ethyl acetate: petroleum ether = 1:2, *v*/*v*). R_f_ = 0.23 on silica gel (ethyl acetate: petroleum ether = 1:2, *v*/*v*). The *ee* was determined to be 54% using HPLC analysis on a CHIRALCEL AD column, λ = 254 nm. Flow rate = 0.5 mL / min; Retention times in 10% 2-propanol in hexanes were 32.5 min (major) and 36.4 min (minor). m.p.: 178–179 °C; ${\left[\alpha \right]}_{D}^{25}$ = +102.5° (*c* = 59.9 mg, CHCl_3_); IR (thin film, cm^−1^) 3566 (br), 3219 (w), 2978 (s), 2934 (s), 2862 (s), 2805 (s), 1491 (s), 1445 (s), 1416 (m), 1383 (s), 1351 (s), 1297 (m), 1077 (s), 1044 (s), 935 (s), 846 (s); ^1^H-NMR (400 MHz, CDCl_3_) δ 7.60 (1H, d, *J =* 7.2 Hz), 7.31–7.23 (2H, m), 7.10 (1H, d, *J =* 8.4 Hz), 6.95–6.84 (4H, m), 6.57 (1H, dd, *J =* 2.4 Hz, *J =* 2.4 Hz), 6.15 (1H, dd, *J =* 2.0 Hz, *J =* 2.4 Hz), 4.93 (1H, *J =* 11.6 Hz), 3.73 (3H, s), 3.51 (1H, dt, *J =* 11.6 Hz, *J =* 2.4 Hz), 3.32 (1H, br), 3.19–3.08 (4H, m), 3.01–2.97 (2H, m), 2.76–2.71 (2H, m); ^13^C-NMR (100 MHz, CDCl_3_) δ 154.2, 145.8, 137.3, 132.0, 129.6, 128.1, 127.7, 126.5, 125.0, 124.7, 118.6, 114.7, 68.0, 67.7, 55.8, 51.5, 49.3. MS (ESI): calcd *m*/*z* for C_21_H_24_N_2_O_2_ (M^+^) 336.18, found: 337.10 [M + H]^+^. Anal. Calcd for C_21_H_24_N_2_O_2_: C, 74.97; H, 7.19; N, 8.33. Found: C, 74.77; H, 7.40; N, 8.25.

*(1S,2S)-2-[4-(2,5-Dimethyl-phenyl)-piperazin-1-yl]-1,2-dihydro-naphthalen-1-ol* (**2k**). Prepared according to general procedure. **2k** was obtained as a white solid (119 mg, 98%) by flash chromatography (ethyl acetate: petroleum ether = 1:4, *v*/*v*). R_f_ = 0.32 on silica gel (ethyl acetate: petroleum ether = 1:4, *v*/*v*). The *ee* was determined to be 36% using HPLC analysis on a CHIRALCEL AD column, λ = 254 nm. Flow rate = 0.5 mL/min; Retention times in 10% 2-propanol in hexanes were 13.8 min (major) and 14.3 min (minor). m.p.: 128–130 °C; ${\left[\alpha \right]}_{D}^{25}$ = +100.0° (*c* = 44.7 mg, CHCl_3_); IR (thin film, cm^−1^) 3509 (br), 3216 (w), 2977 (s), 2934 (s), 2862 (s), 2806 (s), 1490 (s), 1445 (s), 1415 (s), 1383 (s), 1351 (s), 1298 (m), 1127 (s), 1077 (s), 1044 (m), 935 (s), 846 (s); ^1^H-NMR (400 MHz, CDCl_3_) δ 7.52 (1H, d, *J =* 7.2 Hz), 7.21–7.13 (2H, m), 6.98 (2H, t, *J =* 6.8 Hz), 6.73 (2H, t, *J =* 11.6 Hz, *J =* 7.6 Hz), 6.47 (1H, dd, *J =* 2.4 Hz, *J =* 2.8 Hz), 6.10 (1H, dd, *J =* 2.4 Hz, *J =* 2.4 Hz), 4.84 (1H, 12.0), 3.42 (1H, dt, *J =* 12.0 Hz, *J =* 2.4 Hz), 3.37 (1H, br), 2.90–2.82 (6H, m), 2.64–2.58 (2H, m), 2.23 (3H, s), 2.19 (3H, s); ^13^C-NMR (100 MHz, CDCl_3_) δ 151.4, 137.3, 136.3, 132.0, 131.1, 129.4, 129.4, 128.0, 127.5, 126.3, 125.0, 124.8, 124.0, 119.9, 67.9, 67.7, 52.4, 49.3, 21.4, 17.7. MS (ESI): calcd *m*/*z* for C_22_H_26_N_2_O (M^+^) 334.20, found: 335.23 [M + H]^+^. Anal. Calcd for C_22_H_26_N_2_O: C, 79.00; H, 7.84; N, 8.38. Found: C, 78.83; H, 8.12; N, 8.32.

*(1S,2S)-2-[4-(2,5-Difluoro-phenyl)-piperazin-1-yl]-1,2-dihydro-naphthalen-1-ol* (**2l**). Prepared according to general procedure. **2l** was obtained as a white solid (115 mg, 97%) by flash chromatography (ethyl acetate: petroleum ether = 1:4, *v*/*v*). The absolute stereochemistry was determined by X-ray crystallography. R_f_ = 0.14 on silica gel (ethyl acetate: petroleum ether = 1:4, *v*/*v*). The *ee* was determined to be 43% using HPLC analysis on a CHIRALCEL AD column, λ = 254 nm. Flow rate = 0.5 mL/min; Retention times in 10% 2-propanol in hexanes were 20.2 min (major) and 23.2 min (minor). m.p.: 122–124 °C; ${\left[\alpha \right]}_{D}^{25}$ = +150.7° (*c* = 26.8 mg, CHCl_3_); IR (thin film, cm^−1^) 3507 (br), 3203 (w), 2988 (s), 2943 (s), 2872 (s), 1509 (s), 1445 (s), 1383 (s), 1297 (m), 1131 (s), 935 (s), 846 (s), 793 (s); ^1^H-NMR (400 MHz, CDCl_3_) δ 7.59 (1H, d, *J =* 7.2 Hz), 7.31–7.23 (2H, m), 7.09 (1H, d, *J =* 6.0 Hz), 6.95–6.78 (3H, m), 6.57 (1H, dd, *J =* 2.4 Hz, *J =* 2.4 Hz), 6.16 (1H, dd, *J =* 2.8 Hz, *J =* 2.8 Hz), 4.93 (1H, 11.6), 3.53 (1H, dt, *J =* 11.6 Hz, *J =* 2.8 Hz), 3.31 (1H, br), 3.13–2.96 (4H, m), 2.96–2.92 (2H, m), 2.75–2.70 (2H, m); ^13^C-NMR (100 MHz, CDCl_3_) δ 159.4, 157.0, 154.6, 137.2, 136.9, 131.9, 129.6, 128.1, 127.7, 126.4, 125.0, 119.7, 111.0, 110.8, 105.2, 104.7, 67.9, 67.7, 51.7, 49.2. MS (ESI): calcd *m*/*z* for C_20_H_20_F_2_N_2_O (M^+^) 342.15, found: 343.20 [M + H]^+^. Anal. Calcd for C_20_H_20_F_2_N_2_O: C, 70.16; H, 5.89; N, 8.18. Found: C, 70.19; H, 5.94; N, 8.26.

*(1S,2S)-2-[4-(2,3-Dimethyl-phenyl)-piperazin-1-yl]-1,2-dihydro-naphthalen-1-ol* (**2m**). Prepared according to general procedure. **2m** was obtained as a white solid (102 mg, 88%) by flash chromatography (ethyl acetate: petroleum ether = 1:4, *v*/*v*). R_f_ = 0.29 on silica gel (ethyl acetate: petroleum ether = 1:4, *v*/*v*). The *ee* was determined to be 47% using HPLC analysis on a CHIRALCEL AD column, λ = 254 nm. Flow rate = 0.5 mL/min; Retention times in 10% 2-propanol in hexanes were 13.5 min (major) and 14.4 min (minor). m.p.: 163–164 °C; ${\left[\alpha \right]}_{D}^{25}$ = +82.7° (*c* = 99.7 mg, CHCl_3_); IR (thin film, cm^−1^); 3590 (br), 3228 (w), 2974 (s), 2934 (s), 2873 (s), 2806 (s), 1490 (s), 1445 (s), 1415 (m), 1383 (s), 1351 (s), 1297 (m), 1133 (s), 1077 (s), 935 (s), 846 (s); ^1^H-NMR (400 MHz, CDCl_3_) δ 7.60 (1H, d, *J =* 7.2 Hz), 7.27–7.23 (2H, m), 7.08 (2H, t, *J =* 7.2 Hz), 6.91 (2H, dd, *J =* 4.8 Hz, *J =* 4.4 Hz), 6.55 (1H, dd, *J =* 2.8 Hz, *J =* 2.4 Hz), 6.19 (1H, dd, *J =* 2.4 Hz, *J =* 2.4 Hz), 4.92 (1H, *J =* 14.6 Hz), 3.50 (1H, dt, *J =* 11.6 Hz, *J =* 2.8 Hz), 3.41 (1H, br), 2.97–2.88 (6H, m), 2.69 (2H, t, *J =* 2.4 Hz), 2.27 (3H, s), 2.24 (3H, s); ^13^C-NMR (100 MHz, CDCl_3_) δ 151.7, 138.3, 137.5, 132.1, 131.5, 129.6, 128.2, 127.7, 126.5, 126.2, 125.4, 125.2, 125.0, 116.9, 68.0, 67.8, 53.0, 49.7, 21.0, 14.3. MS (ESI): calcd *m*/*z* for C_22_H_26_N_2_O (M^+^) 334.20, found: 335.28 [M + H]^+^. Anal. Calcd for C_22_H_26_N_2_O: C, 79.00; H, 7.84; N, 8.38. Found: C, 78.84; H, 8.17; N, 8.27.

*(1S,2S)-2-[4-(1-Hydroxy1,2-dihydro-naphthalen-2-yl-piperazin-1-yl]–benzo-nitrile* (**2n**). Prepared according to general procedure. **2n** was obtained as a white solid (109 mg, 95%) by flash chromatography (ethyl acetate: petroleum ether = 1:2, *v*/*v*). R_f_ = 0.20 on silica gel (ethyl acetate: petroleum ether = 1:2, *v*/*v*). The *ee* was determined to be 54% using HPLC analysis on a CHIRALCEL AD column, λ = 254 nm. Flow rate = 0.5 mL/min; Retention times in 10% 2-propanol in hexanes were 43.6 min (major) and 53.4 min (minor). m.p.: 126–127 °C; ${\left[\alpha \right]}_{D}^{25}$ = +111.4° (*c* = 64.9 mg, CHCl_3_); IR (thin film, cm^−1^); 3692 (br), 3210 (w), 2934 (s), 2874 (s), 2805 (s), 2272 (w), 1627 (w), 1597 (w), 1491 (s), 1445 (s), 1383 (s), 1298 (s), 1118 (s), 1077 (s), 935 (s), 846 (s), 795 (m); ^1^H-NMR (400 MHz, CDCl_3_) δ 7.61–7.57 (2H, m), 7.51 (1H, td, *J =* 7.6 Hz, *J =* 1.6 Hz), 7.31–7.23 (2H, m), 7.10 (1H, d, *J =* 5.6 Hz), 7.05–7.02 (2H, m), 6.58 (1H, dd, *J =* 2.4 Hz, *J =* 2.4 Hz), 6.18 (1H, dd, *J =* 2.4 Hz, *J =* 2.8 Hz), 6.15 (1H, dd, *J =* 2.0 Hz, *J =* 2.4 Hz), 4.94 (1H, *J =* 11.2 Hz), 3.53 (1H, dt, *J =* 11.6 Hz, *J =* 2.4 Hz), 3.33–3.23 (4H, m), 3.07–3.01 (2H, m), 2.82–2.76 (2H, m); ^13^C-NMR (100 MHz, CDCl_3_) δ 155.8, 137.1, 134.5, 134.0, 132.5, 131.9, 128.1, 127.7, 126.4, 124.9, 122.1, 118.6, 106.3, 67.9, 67.7, 52.3, 49.2. MS (ESI): calcd *m*/*z* for C_21_H_21_N_3_O (M^+^) 331.17, found: 332.22 [M + H]^+^. Anal. Calcd for C_21_H_21_N_3_O: C, 76.11; H, 6.39; N, 12.68. Found: C, 76.01; H, 6.69; N, 12.51.

*(1S,2S)-2-[4-(3,4-Dimethyl-phenyl)-piperazin-1-yl]-1,2-dihydro-naphthalen-1-ol* (**2o**). Prepared according to general procedure. **2o** was obtained as a white solid (111 mg, 96%) by flash chromatography (ethyl acetate: petroleum ether = 1:4, *v*/*v*). R_f_ = 0.20 on silica gel (ethyl acetate: petroleum ether = 1:4, *v*/*v*). The *ee* was determined to be 58% using HPLC analysis on a CHIRALCEL AD column, λ = 254 nm. Flow rate = 0.5 mL/min; Retention times in 10% 2-propanol in hexanes were 22.6 min (minor) and 23.8 min (major). m.p.: 125–126 °C; ${\left[\alpha \right]}_{D}^{25}$ = +109.7° (*c* = 59.5 mg, CHCl_3_); IR (thin film, cm^−1^); 3512 (br), 3211 (w), 2974 (s), 2806 (s), 1615 (s), 1490 (s), 1445 (s), 1416 (s), 1383 (m), 1298 (s), 1142 (s), 1044 (s), 935 (s), 846 (s); ^1^H-NMR (400 MHz, CDCl_3_) δ 7.59 (1H, d, *J =* 7.2 Hz), 7.31–7.23 (2H, m), 7.07 (1H, d, *J =* 7.2 Hz), 7.04 (1H, d, *J =* 7.2 Hz), 6.78 (1H, d, *J =* 2.4 Hz), 6.71 (1H, dd, *J =* 2.4 Hz, *J =* 2.4 Hz), 6.56 (1H, dd, *J =* 2.4 Hz, *J =* 2.8 Hz), 6.15 (1H, dd, *J =* 2.0 Hz, *J =* 2.4 Hz), 4.93 (1H, 12.0), 3.53 (1H, dt, *J =* 11.6 Hz, *J =* 2.8 Hz), 3.37 (1H, br), 3.24–3.14 (4H, m), 3.01–2.96 (2H, m), 2.75–2.70 (2H, m), 2.25 (3H, s), 2.20 (3H, s); ^13^C-NMR (100 MHz, CDCl_3_) δ 149.8, 137.3, 137.2, 131.9, 130.4, 129.6, 128.5, 128.1, 127.6, 126.4, 124.9, 124.7, 118.5, 114.2, 67.9, 67.7, 50.6, 49.2, 20.4, 19.0. MS (ESI): calcd *m*/*z* for C_22_H_26_N_2_O (M^+^) 334.20, found: 335.28 [M + H]^+^. Anal. Calcd for C_22_H_26_N_2_O: C, 79.00; H, 7.84; N, 8.38. Found: C, 78.72; H, 8.31; N, 8.48.

*(1S,2S)-2-[4-p-Tolyl-piperazin-1-yl]-1,2-dihydro-naphthalen-1-ol* (**2p**). Prepared according to general procedure. **2p** was obtained as a white solid (94 mg, 85%) by flash chromatography (ethyl acetate: petroleum ether = 1:3 , *v*/*v*). R_f_ = 0.28 on silica gel (ethyl acetate: petroleum ether = 1:3, *v*/*v*). The *ee* was determined to be 27% using HPLC analysis on a CHIRALCEL AD column, λ = 254 nm. Flow rate = 0.5 mL/min; Retention times in 10% 2-propanol in hexanes were 23.0 min (major) and 23.6 min (minor). m.p.: 193–194 °C; ${\left[\alpha \right]}_{D}^{25}$ = +108.9° (*c* = 57.1 mg, CHCl_3_); IR (thin film, cm^−1^) 3422 (br), 3022 (w), 2918 (w), 2833 (m), 1649 (s), 1515 (s), 1499 (s), 1382 (s), 1241 (s), 1140 (s), 1046 (s), 781 (s), 746 (s); ^1^H-NMR (400 MHz, CDCl_3_) δ 7.60 (1H, d, *J =* 6.8 Hz), 7.31–7.23 (2H, m), 7.09 (3H, d, *J =* 7.2 Hz), 6.86 (2H, d, *J =* 7.2 Hz), 6.57 (1H, dd, *J =* 2.4 Hz, *J =* 2.4 Hz), 6.15 (1H, dd, *J =* 2.4 Hz, *J =* 2.4 Hz), 4.94 (1H, d, *J =* 11.6 Hz), 3.52 (1H, dt, *J =* 11.2 Hz, *J =* 2.8 Hz), 3.36 (1H, br), 3.24–3.14 (4H, m), 3.01–2.96 (2H, m), 2.75–2.70 (2H, m), 2.29 (3H, s); ^13^C-NMR (100 MHz, CDCl_3_) δ 149.4, 137.2, 131.9, 129.9, 129.7, 129.6, 128.1, 127.6, 126.4, 124.9, 124.6, 116.8, 67.9, 67.7, 50.6, 49.2, 20.6. MS (ESI): calcd *m*/*z* for C_21_H_24_N_2_O (M^+^) 320.19, found: 321.25 [M + H]^+^. Anal. Calcd for C_21_H_24_N_2_O: C, 78.71; H, 7.55; N, 8.74. Found: C, 78.52; H, 7.81; N, 8.66.

*(1S,2S)-2-(4-Benzo[1,3] dioxol-5-yl-piperazin-1-yl]-1,2-dihydro-naphthalen-1-ol* (**2q**). Prepared according to general procedure. **2q** was obtained as a white solid (108 mg, 86%) by flash chromatography (ethyl acetate: petroleum ether = 1:1, *v*/*v*). R_f_ = 0.27 on silica gel (ethyl acetate: petroleum ether = 1:1, *v*/*v*). The *ee* was determined to be 57% using HPLC analysis on a CHIRALCEL AD column, λ = 254 nm. Flow rate = 1.0 mL/min; Retention times in 10% 2-propanol in hexanes were 20.5 min (minor) and 25.5 min (major). m.p.: 112–113 °C; ${\left[\alpha \right]}_{D}^{25}$ = +81.8° (*c* = 99.3 mg, CHCl_3_); IR (thin film, cm^−1^) 3514 (br), 3211 (w), 2925 (s), 2868 (s), 1627 (w), 1488 (s), 1446 (s), 1415 (s), 1298 (s), 1044 (s), 935 (s), 846 (s), 795 (s); ^1^H-NMR (400 MHz, CDCl_3_) δ 7.59 (1H, d, *J* = 6.8 Hz), 7.29–7.25 (2H, m), 7.09 (1H, d, *J* = 8.4 Hz), 6.89 (1H, s), 6.78 (2H, s), 6.55 (1H, dd, *J* = 2.4 Hz, *J =* 2.8 Hz), 6.45 (1H, dd, *J* = 2.0, *J =* 2.4 Hz), 5.97 (2H, s), 4.90 (1H, d, *J =* 11.2 Hz), 3.49–3.46 (4H, m), 2.89–2.84 (2H, m), 2.63–2.53 (6H, m); ^13^C-NMR (100 MHz, CDCl_3_) δ 147.8, 146.8, 137.3, 132.0, 132.0, 129.3, 128.0, 127.6, 126.3, 125.0, 124.8, 122.5, 109.7, 108.1, 67.9, 67.5, 62.9, 53.6, 49.0. MS (ESI): calcd *m*/*z* for C_22_H_24_N_2_O_3_ (M^+^) 364.18. Found 365.26 [M + H]^+^. Anal. Calcd for C_22_H_24_N_2_O_3_: C, 72.50; H, 6.64; N, 7.69. Found: C, 72.29; H, 6.44; N, 7.76.

*(1S,2S)-2-[4-(2,4-Dimethyl-phenyl)-piperazin-1-yl]-1,2-dihydro-naphthalen-1-ol* (**2r**). Prepared according to general procedure. **2r** was obtained as a white solid (103 mg, 89%) by flash chromatography (ethyl acetate: petroleum ether = 1:4, *v*/*v*). R_f_ = 0.26 on silica gel (ethyl acetate: petroleum ether = 1:4, *v*/*v*). The *ee* was determined to be 59% using HPLC analysis on a CHIRALCEL AD column, λ = 254 nm. Flow rate = 0.5 mL/min; Retention times in 10% 2-propanol in hexanes were 13.1 min (major) and 14.7 min (minor). m.p.: 113–114 °C; ${\left[\alpha \right]}_{D}^{25}$ = +67.6° (*c* = 65.1 mg, CHCl_3_); IR (thin film, cm^−1^) 3589 (br), 3209 (w), 2990 (s), 2806 (s), 2778 (s), 1627 (w), 1491 (s), 1445 (s), 1415 (s), 1383 (s), 1298 (m), 1141 (s), 1076 (s), 935 (s), 846 (s); ^1^H-NMR (400 MHz, CDCl_3_) δ 7.62 (1H, d, *J* = 7.6 Hz), 7.31–7.23 (2H, m), 7.09 (1H, d, *J* = 7.2 Hz, *J =* 2.4 Hz), 7.02–6.95 (3H, m), 6.57 (1H, dd, *J* = 2.4 Hz, *J =* 2.4 Hz), 6.21 (1H, dd, *J* = 2.4 Hz, *J =* 2.4 Hz), 4.94 (1H, d, *J =* 11.6 Hz), 3.51 (1H, dt, *J =* 12 Hz, *J =* 2.4 Hz), 3.38 (1H, s), 2.99–2.90 (6H, m), 3.72–2.69 (2H, m), 2.31 (6H, s); ^13^C-NMR (100 MHz, CDCl_3_) δ 149.2, 137.4, 132.8, 132.6, 132.0, 132.0, 129.4, 128.0, 127.6, 127.2, 126.3, 125.1, 124.9, 119.1, 67.9, 67.7, 52.6, 49.6, 20.9, 17.9. MS (ESI): calcd *m*/*z* for C_22_H_26_N_2_O (M^+^) 334.20, found: 335.25 [M + H]^+^. Anal. Calcd for C_22_H_26_N_2_O: C, 79.00; H, 7.84; N, 8.38. Found: C, 79.03; H, 8.13; N, 8.27.

*(1S,2S)-2-[4-m-Tolyl-piperazin-1-yl]-1,2-dihydro-naphthalen-1-ol* (**2s**). Prepared according to general procedure. **2s** was obtained as a white solid (100 mg, 90%) by flash chromatography (ethyl acetate: petroleum ether = 1:4, *v*/*v*). R_f_ = 0.26 on silica gel (ethyl acetate: petroleum ether = 1:4, *v*/*v*). The *ee* was determined to be 59% using HPLC analysis on a CHIRALCEL AD column, λ = 254 nm. Flow rate = 1.0 mL/min; Retention times in 10% 2-propanol in hexanes were 22.2 min (minor) and 22.7 min (major). m.p.: 94–96 °C; ${\left[\alpha \right]}_{D}^{25}$ = +107.4° (*c* = 46.2 mg, CHCl_3_); IR (thin film, cm^−1^) 3507 (br), 3158 (w), 2978 (s), 2934 (s), 2861 (s), 2806 (m), 1491 (s), 1445 (s), 1416 (s), 1383 (s), 1351 (s), 1298 (m), 1154 (s), 1077 (s), 935 (s), 846 (s); ^1^H-NMR (400 MHz, CDCl_3_) δ 7.69 (1H, d, *J =* 7.2 Hz), 7.36 (2H, td, *J =* 2.4 Hz, *J =* 7.2 Hz), 7.27 (1H, t, *J =* 7.6 Hz), 7.19 (1H, d, *J =* 7.2 Hz), 6.84 (3H, dd, *J =* 4.8 Hz, *J =* 7.6 Hz), 6.66 (1H, dd, *J =* 2.4 Hz, *J =* 2.4 Hz), 6.23 (1H, dd, *J =* 2.4 Hz, *J =* 2.4 Hz), 5.03 (1H, d, *J =* 11.6 Hz), 3.62 (1H, dt, *J =* 11.6 Hz, *J =* 2.0 Hz), 3.43 (1H, br), 3.36–3.27 (4H, m), 3.09–3.04 (2H, m), 2.83–2.78 (2H, m); ^13^C-NMR (100 MHz, CDCl_3_) δ 151.5, 139.0, 137.2, 131.9, 129.7, 129.1, 128.1, 127.6, 126.4, 124.6, 121.0, 117.3, 113.6, 67.9, 67.7, 50.1, 49.2, 21.9. MS (ESI): calcd *m*/*z* for C_21_H_24_N_2_O (M^+^) 320.19, found: 321.25 [M + H]^+^. Anal. Calcd for C_21_H_24_N_2_O: C, 78.71; H, 7.55; N, 8.74. Found: C, 78.69; H, 7.94; N, 8.56.

*(1S,2S)-2-(4-Phenethyl-cyclohexyl)-1,2-dihydro-naphthalen-1-ol* (**2t**). Prepared according to general procedure. **2t** was obtained as a white solid (90 mg, 78%) by flash chromatography (ethyl acetate: petroleum ether = 1:2, *v*/*v*). R_f_ = 0.20 on silica gel (ethyl acetate: petroleum ether = 1:2, *v*/*v*). The *ee* was determined to be 50% using HPLC analysis on a CHIRALCEL AD column, λ = 254 nm. Flow rate = 1.0 mL/min; Retention times in 10% 2-propanol in hexanes were 10.1 min (major) and 11.4 min (minor). m.p.: 112–113 °C; ${\left[\alpha \right]}_{D}^{25}$ = +87.9° (*c* = 36.5 mg, CHCl_3_); IR (thin film, cm^−1^) 3519 (br), 3089 (w), 2978 (s), 2934 (s), 2863 (s), 2805 (s), 1490 (s), 1445 (s), 1415 (s), 1383 (s), 1351 (s), 1297 (m), 1076 (s), 1044 (s), 935 (s), 846 (s); ^1^H-NMR (400 MHz, CDCl_3_) δ 7.60 (1H, d, *J =* 7.2 Hz), 7.34–7.21 (7H, m), 7.09 (1H, d, *J =* 1.2 Hz), 6.56 (1H, dd, *J =* 2.4 Hz, *J =* 2.4 Hz), 6.15 (1H, dd, *J =* 2.4 Hz, *J =* 2.4 Hz), 4.91 (1H, d, *J =* 11.6 Hz), 3.48 (2H, ddd, *J =* 2.4 Hz, *J =* 2.4 Hz, *J =* 6.8 Hz), 2.85–2.96 (4H, m), 2.65 (8H, dd, *J =* 4.8 Hz, *J =* 6.4 Hz); ^13^C-NMR (100 MHz, CDCl_3_) δ 140.5, 137.4, 132.1, 129.5, 128.9, 128.8, 128.7, 128.6, 128.1, 127.6, 126.3, 125.1, 67.7, 67.6, 60.7, 53.9, 49.1, 33.9. MS (ESI): calcd *m*/*z* for C_22_H_26_N_2_O (M^+^) 334.20, found: 335.18 [M + H]^+^. Anal. Calcd for C_22_H_26_N_2_O: C, 79.00; H, 7.84; N, 8.38. Found: C, 79.89; H, 8.05; N, 8.26.

*(1S,2S)-4-(1-Hydroxy-1,2-dihydro-naphthalen-2-yl)-piperazine-1-carboxy-lic acid ethyl ester* (**2u**). Prepared according to general procedure. **2u** was obtained as a white solid (89 mg, 85%) by flash chromatography (ethyl acetate: petroleum ether = 1:2, *v*/*v*). R_f_ = 0.17 on silica gel (ethyl acetate: petroleum ether = 1:2, *v*/*v*). The *ee* was determined to be 51% using HPLC analysis on a CHIRALCEL AD column, λ = 254 nm. Flow rate = 1.0 mL/min; Retention times in 10% 2-propanol in hexanes were 16.0 min (minor) and 21.7 min (major). m.p.: 147–148 °C; ${\left[\alpha \right]}_{D}^{25}$ = +93.2° (*c* = 65.3 mg, CHCl_3_); IR (thin film, cm^−1^) 3517 (br), 3087 (w), 2977 (s), 2934 (s), 2867 (s), 2806 (s), 1710 (m), 1490 (s), 1445 (s), 1415 (m), 1383 (s), 1351 (s), 1298 (m), 1077 (s), 1044 (m), 935 (m), 846 (s); ^1^H-NMR (400 MHz, CDCl_3_) δ 7.57 (1H, d, *J =* 7.6 Hz), 7.31–7.23 (2H, m), 7.09 (1H, d, *J =* 6.8 Hz), 6.56 (1H, dd, *J =* 2.0 Hz, *J =* 2.0 Hz), 6.03 (1H, dd, *J =* 2.4 Hz, *J =* 2.8 Hz), 4.90 (1H, d, *J =* 10.8 Hz), 4.16 (2H, dd, *J =* 14.4 Hz, *J =* 6.8 Hz), 3.56–3.47 (5H, m), 3.38 (1H, br), 2.74 (2H, t, *J =* 6.8 Hz), 2.54 (2H, t, *J =* 6.0 Hz), 1.29 (3H, t, *J =* 7.2 Hz); ^13^C-NMR (100 MHz, CDCl_3_) δ 155.5, 137.0, 131.8, 129.5, 128.0, 127.6, 126.3, 125.2, 124.5, 67.8, 67.6, 61.4, 48.9, 44.2, 14.7. MS (ESI): calcd *m*/*z* for C_17_H_22_N_2_O_3_ (M^+^) 302.16, found: 303.08 [M + H]^+^. Anal. Calcd for C_1__7_H_22_N_2_O_3_: C, 67.53; H, 7.33; N, 9.26. Found: C, 67.39; H, 7.68; N, 9.15.

*(1S,2S)-2-[4-(3-Trifluoromethyl-phenyl)-piperazin-1-yl]-1,2-dihydro–naphthalen-1-ol* (**2v**). Prepared according to general procedure. **2v** was obtained as a white solid (107 mg, 83%) by flash chromatography (ethyl acetate: petroleum ether = 1:3, *v*/*v*). R_f_ = 0.26 on silica gel (ethyl acetate: petroleum ether = 1:3, *v*/*v*). The *ee* was determined to be 54% using HPLC analysis on a CHIRALCEL AD column, λ = 254 nm. Flow rate = 1.0 mL/min; Retention times in 2% 2-propanol in hexanes were 35.2 min (minor) and 36.3 min (major). m.p.: 120–121 °C; ${\left[\alpha \right]}_{D}^{25}$ = +87.6° (*c* = 46.8 mg, CHCl_3_); IR (thin film, cm^−1^) 3517 (br), 3092 (w), 2978 (s), 2869 (s) 1628 (w), 1491 (m), 1444 (s), 1383 (s), 1351 (s), 1297 (m), 1133 (s), 1077 (s), 934 (s), 846 (s); ^1^H-NMR (400 MHz, CDCl_3_) δ 7.59 (1H, d, *J =* 7.2 Hz), 7.34 (1H, t, *J =* 8.0 Hz), 7.29–7.23 (2H, m), 7.13–7.06 (4H, m), 6.57 (1H, d, *J =* 10.0 Hz), 6.11 (1H, dd, *J =* 2.0 Hz, *J =* 2.0 Hz), 4.94 (1H, d, *J =* 12.0 Hz), 3.53 (1H, dd, *J =* 2.4 Hz, *J =* 2.0 Hz), 3.30–3.22 (5H, m), 2.97 (2H, dd, *J =* 3.2 Hz, *J =* 5.2 Hz), 2.72 (2H, t, *J =* 11.2 Hz); ^13^C-NMR(100 MHz, CDCl_3_) δ 151.5, 137.1, 131.9, 129.8, 129.7, 128.2, 127.8, 126.5, 125.1, 124.7, 119.1, 116.2, 116.2, 112.5, 112.5, 68.0, 67.6, 49.5, 49.0. MS (ESI): calcd *m*/*z* for C_21_H_21_F_3_N_2_O (M^+^) 374.16, found: 375.12 [M + H]^+^. Anal. Calcd for C_21_H_21_F_3_N_2_O: C, 67.37; H, 5.65; N, 7.48. Found: C, 67.16; H, 5.89; N, 7.36.

*(1S,2S)-2-[4-(4-Trifluoromethyl-phenyl)-piperazin-1-yl]-1,2-dihydro-naphth-alen-1-ol* (**2w**). Prepared according to general procedure. **2w** was obtained as a white solid (98 mg, 76%) by flash chromatography (ethyl acetate: petroleum ether = 1:3, *v*/*v*). R_f_ = 0.20 on silica gel (ethyl acetate: petroleum ether = 1:3, *v*/*v*). The *ee* was determined to be 56% using HPLC analysis on a CHIRALCEL AD column, λ = 254 nm. Flow rate = 1.0 mL/min; Retention times in 10% 2-propanol in hexanes were 13.7 min (major) and 15.5 min (minor). m.p.: 215–216 °C; ${\left[\alpha \right]}_{D}^{25}$ = +102.8° (*c* = 52.7 mg, CHCl_3_); IR (thin film, cm^−1^) 3591 (br), 3210 (w), 2978 (s), 2873 (s), 2805 (s) 1617 (w), 1490 (s), 1445 (s), 1416 (s), 1389 (s), 1297 (s), 1131 (s), 1077 (s), 935 (s), 845 (s); ^1^H-NMR (400 MHz, CDCl_3_) δ 7.51 (1H, d, *J =* 7.2 Hz), 7.42 (2H, d, *J =* 8.8 Hz), 7.18 (2H, m), 7.02 (1H, d, *J =* 7.2 Hz), 6.86 (2H, d, *J =* 8.8 Hz), 6.50 (1H, dd, *J =* 2.8 Hz, *J =* 2.4 Hz), 6.03 (1H, dd, *J =* 2.4 Hz, *J =* 2.8 Hz), 4.86 (1H, d, *J =* 11.2 Hz), 3.47 (1H, d, *J =* 11.2 Hz), 3.30–3.20 (5H, m), 2.93–2.88 (2H, m), 2.68–2.63 (2H, m); ^13^C-NMR (100 MHz, CDCl_3_) δ 153.5, 137.1, 131.9, 129.9, 128.3, 127.8, 126.7, 126.7, 126.7, 126.6, 126.5, 125.1, 124.2, 114.9, 68.1, 67.8, 49.0, 48.9. MS (ESI): calcd *m*/*z* for C_21_H_21_F_3_N_2_O (M^+^) 374.16, found: 375.25 [M + H]^+^. Anal. Calcd for C_21_H_21_F_3_N_2_O: C, 67.37; H, 5.65; N, 7.48. Found: C, 67.13; H, 5.86; N, 7.36.

*(1S,2S)-2-[4-(4-Chloro-phenyl)-piperazin-1-yl]-1,2-dihydro-naphthalen-1-ol* (**2x**). Prepared according to general procedure. **2x** was obtained as a white solid (97 mg, 82%) by flash chromatography (ethyl acetate: petroleum ether = 1:2, *v*/*v*). R_f_ = 0.3 on silica gel (ethyl acetate: petroleum ether = 1:2, *v*/*v*). The *ee* was determined to be 39% using HPLC analysis on a CHIRALCEL AD column, λ = 254 nm. Flow rate = 1.0 mL/min; Retention times in 10% 2-propanol in hexanes were 15.2 min (major) and 16.9 min (minor). m.p.: 203–204 °C; ${\left[\alpha \right]}_{D}^{25}$ = +100.1° (*c* = 65.1 mg, CHCl_3_); IR (thin film, cm^−1^) 3585 (br), 3212 (w), 2978 (s), 2935 (s), 2806 (s) 1634 (w), 1490 (s), 1445 (s), 1415 (s), 1351 (s), 1298 (s), 1134 (s), 1076 (s), 935 (s), 845 (s); ^1^H-NMR (400 MHz, CDCl_3_) δ 7.60 (d, 1H, *J =* 7.2 Hz), 7.30–7.22 (m, 4H), 7.1 (d, 1H, *J =* 7.2 Hz), 6.86 (d, 2H, *J =* 8.8 Hz), 6.58 (dd, 1H, *J =* 2.0 Hz, *J =* 2.4 Hz), 6.13 (dd, 1H, *J =* 2.4 Hz, *J =* 2.4 Hz), 4.94 (d, 1H, *J =* 11.6 Hz), 3.54 (d, 1H, *J =* 11.6 Hz), 3.24–3.19 (m, 5H), 3.00–2.96 (m, 2H), 2.76–2.71 (m, 2H); ^13^C-NMR (100 MHz, CDCl_3_) δ 150.1, 137.2, 131.9, 129.8, 129.2, 128.2, 127.8, 126.5, 125.1, 125.0, 124.4, 117.6, 68.0, 67.7, 50.0, 49.1. MS (ESI): calcd *m*/*z* for C_20_H_21_ClN_2_O (M^+^) 340.13, found: 341.20 [M + H]^+^. Anal. Calcd for C_20_H_21_ClN_2_O: C, 70.48; H, 6.21; N, 8.22. Found: C, 70.29; H, 6.44; N, 8.16.

*(1S,2S)-2-[4-(1-Hydroxy-6,7-dimethoxy-1,2-dihydro-naphthalen-2-yl)-piper-azin-1-yl]-benzonitrile* (**3a**). Prepared according to general procedure. **3a** was obtained as a white solid (74 mg, 77%) by flash chromatography (ethyl acetate: petroleum ether = 1:1, *v*/*v*). R_f_ = 0.16 on silica gel (ethyl acetate: petroleum ether = 1:1, *v*/*v*). The *ee* was determined to be 37% using HPLC analysis on a CHIRALCEL AD column, λ = 254 nm. Flow rate = 1.0 mL/min; Retention times in 10% 2-propanol in hexanes were 73.4 min (major) and 86.0 min (minor). m.p.: 192–193 °C; ${\left[\alpha \right]}_{D}^{25}$ = +65.5° (*c* = 37.8 mg, CHCl_3_); IR (thin film, cm^−1^) 3512 (br), 3018 (w), 2927 (s), 2809 (s), 2271 (w), 1635 (w), 1490 (s), 1445 (s), 1351 (s), 1297 (s), 1135 (s), 1078 (s), 935 (s), 846 (s); ^1^H-NMR (400 MHz, CDCl_3_) δ 7.57 (1H, dd, *J* = 1.2 Hz, *J =* 1.2 Hz), 7.48 (1H, t, *J =* 7.2 Hz), 7.13 (1H, s), 7.03–6.99 (2H, m), 6.45 (1H, s), 6.47 (1H, dd, *J =* 2.4 Hz, *J =* 2.4 Hz), 6.07 (1H, dd, *J =* 2.4 Hz, *J =* 2.4 Hz), 4.86 (1H, d, *J =* 11.2 Hz), 3.93 (3H, s), 3.87 (3H, s), 3.45 (1H, d, *J =* 7.2 Hz), 3.28–3.22 (4H, m), 3.15 (1H, br), 2.99–2.98 (2H, m), 2.78–2.77 (2H, m); ^13^C-NMR (100 MHz, CDCl_3_) δ 155.7, 148.8, 148.2, 134.5, 134.0, 130.0, 129.1, 124.8, 122.1, 118.8, 110.2, 108.8, 106.2, 68.0, 67.8, 56.2, 56.2, 52.2, 49.2. MS (ESI): calcd *m*/*z* for C_23_H_25_N_3_O_3_ (M^+^) 391.19, found: 392.10 [M + H]^+^. Anal. Calcd for C_23_H_25_N_3_O_3_: C, 70.57; H, 6.44; N, 10.73. Found: C, 70.46; H, 6.67; N, 10.55.

*(1S,2S)-2-[4-(4-Fluoro-phenyl)-piperazin-1-yl]-6,7-dimethoxy-1,2-dihydro-Naphthalen-1-ol* (**3b**). Prepared according to general procedure. **3b** was obtained as a white solid (69 mg, 73%) by flash chromatography (ethyl acetate: petroleum ether = 1:1, *v*/*v*). R_f_ = 0.20 on silica gel (ethyl acetate: petroleum ether = 1:1, *v*/*v*). The *ee* was determined to be 49% using HPLC analysis on a CHIRALCEL AD column, λ = 254 nm. Flow rate = 1.0 mL/min; Retention times in 10% 2-propanol in hexanes were 40.5 min (major) and 42.9 min (minor). m.p.: 200–201 °C; ${\left[\alpha \right]}_{D}^{25}$ = +79.1° (*c* = 41.2 mg, CHCl_3_); IR (thin film, cm^−1^) 3516 (br), 3022 (w), 2972 (s), 2935 (s), 2806 (s), 1635 (w), 1490 (s), 1445 (s), 1351 (s), 1298 (s), 1135 (s), 1078 (s), 935 (s), 846 (s); ^1^H-NMR (400 MHz, CDCl_3_) δ 7.15 (1H, s), 6.97 (2H, d, *J =* 8.4 Hz), 6.91–6.87 (2H, m), 6.66 (1H, s), 6.49 (1H, dd, *J =* 2.4 Hz, *J =* 2.4 Hz), 6.03 (1H, dd, *J =* 2.8 Hz, *J =* 2.4 Hz), 4.87 (1H, d, *J =* 11.2 Hz), 3.94 (3H, s), 3.88 (3H, s), 3.49 (1H, dt, *J =* 11.2 Hz, *J =* 2.4 Hz), 3.25 (1H, br), 3.18–3.13 (4H, m), 2.96–2.94 (2H, m), 2.74–2.73 (2H, m); ^13^C-NMR (100 MHz, CDCl_3_) δ 158.7, 156.3, 148.9, 148.3, 148.1, 130.0, 129.2, 124.8, 122.5, 118.2, 115.8, 115.6, 110.3, 108.9, 67.9, 67.8, 56.3, 56.3, 51.0, 49.2. MS (ESI): calcd *m*/*z* for C_22_H_25_FN_2_O_3_ (M^+^) 384.18, found: 385.07 [M + H]^+^. Anal. Calcd for C_22_H_25_FN_2_O_3_: C, 68.73; H, 6.55; N, 7.29. Found: C, 68.56; H, 6.78; N, 7.19.

*(1S,2S)-2-[4-(2-Fluoro-phenyl)-piperazin-1-yl]-6,7-dimethoxy-1,2-dihydro-naphthalen-1-ol* (**3c**). Prepared according to general procedure. **3c** was obtained as a white solid (74 mg, 79%) by flash chromatography (ethyl acetate: petroleum ether = 1:1, *v*/*v*). R_f_ = 0.29 on silica gel (ethyl acetate: petroleum ether = 1:1, *v*/*v*). The *ee* was determined to be 38% using HPLC analysis on a CHIRALCEL AD column, λ = 254 nm. Flow rate = 0.5 mL/min; Retention times in 10% 2-propanol in hexanes were 55.6 min (minor) and 60.3 min (major). m.p.: 160–161 °C; ${\left[\alpha \right]}_{D}^{25}$ = 70.9° (*c* = 35.9 mg, CHCl_3_); IR (thin film, cm^−1^) 3507 (br), 3188 (w), 2968 (s), 2890 (s), 2811 (s), 1610 (w), 1458 (s), 1379 (s), 1281 (s), 1183 (s), 1099 (s), 935 (s), 846 (s); ^1^H-NMR (400 MHz, CDCl_3_) δ 7.14 (1H, s), 7.06–6.93 (4H, m), 6.65 (1H, s), 6.47 (1H, dd, *J =* 2.4 Hz, *J =* 2.0 Hz), 6.05 (1H, dd, *J =* 2.4 Hz, *J =* 2.4 Hz), 4.86 (1H, d, *J =* 11.6 Hz), 3.93 (3H, s), 3.87 (3H, s), 3.46 (1H, d, *J =* 11.2 Hz), 3.22 (1H, br), 3.15–3.11 (4H, m), 2.98–2.95 (2H, m), 2.76–2.72 (2H, m); ^13^C-NMR (100 MHz, CDCl_3_) δ 148.8, 148.2, 130.0, 129.1, 124.8, 124.6, 122.7, 119.1, 119.1, 116.4, 116.2, 110.1, 108.8, 67.9, 67.8, 56.2, 56.2, 51.2, 51.2, 49.2. MS (ESI): calcd *m*/*z* for C_22_H_25_FN_2_O_3_ (M^+^) 384.18, found: 385.10 [M + H]^+^. Anal. Calcd for C_22_H_25_FN_2_O_3_: C, 68.73; H, 6.55; N, 7.29. Found: C, 68.62; H, 6.73; N, 7.18.

*(1S,2S)-6,7-Dimethoxy-2-(4-(4-methoxy-phenyl)-piperazin-1-yl]-1,2-dihydro–naphthalene-1-ol* (**3d**). Prepared according to general procedure. **3d** was obtained as a white solid (60 mg, 62%) by flash chromatography (ethyl acetate: petroleum ether = 1:1, *v*/*v*). R_f_ = 0.20 on silica gel (ethyl acetate: petroleum ether = 1:1, *v*/*v*). The *ee* was determined to be 59% using HPLC analysis on a CHIRALCEL AD column, λ = 254 nm. Flow rate = 0.5 mL / min; Retention times in 5% 2-propanol in hexanes were 92.2 min (major) and 110.1 min (minor). m.p.: 174–175 °C; ${\left[\alpha \right]}_{D}^{25}$ = +77.4° (*c* = 26.5 mg, CHCl_3_); IR (thin film, cm^−1^) 3696 (br), 3036 (w), 2924 (s), 2877 (s), 1682 (s), 1512 (s), 1452 (s), 1383 (w), 1244 (s), 1111 (s), 1013 (s), 935 (w), 815 (s); ^1^H-NMR (400 MHz, CDCl_3_) δ 7.15 (1H, s), 6.92 (2H, d, *J =* 2.0 Hz), 6.85 (2H, d, *J =* 2.0 Hz), 6.66 (1H, s), 6.48 (1H, dd, *J =* 2.8 Hz, *J =* 2.4 Hz), 6.05 (1H, dd, *J =* 2.4 Hz, *J =* 2.8 Hz), 4.86 (1H, d, *J =* 11.2 Hz), 3.94 (3H, s), 3.88 (3H, s), 3.77 (3H, s), 3.48 (1H, dt, *J =* 11.2 Hz, *J =* 2.4 Hz), 3.25 (1H, br), 3.15–3.10 (4H, m), 2.98–2.93 (2H, m), 2.75–2.70 (2H, m); ^13^C-NMR (100 MHz, CDCl_3_) δ 154.2, 148.9, 148.3, 145.9, 130.1, 129.1, 124.8, 122.8, 118.6, 114.7, 110.3, 108.9, 68.0, 67.9, 56.3, 56.3, 51.5, 49.3. MS (ESI): calcd *m*/*z* for C_23_H_28_N_2_O_4_ (M^+^) 396.20, found: 397.14 [M + H] ^+^. Anal. Calcd for C_23_H_28_N_2_O_4_: C, 69.67; H, 7.12; N, 7.07. Found: C, 74.89; H, 7.44; N, 8.56.

*(1S,2S)-2-[4-(3,4-Dichloro-phenyl)-piperazin-1-yl]-6,7-dimethoxy-1,2-dihydronaphthalen-1-ol* (**3e**). Prepared according to general procedure. **3e** was obtained as a white solid (50 mg, 47%) by flash chromatography (ethyl acetate: petroleum ether = 2:3, *v*/*v*). R_f_ = 0.20 on silica gel (ethyl acetate: petroleum ether = 2:3, *v*/*v*). The *ee* was determined to be 16% using HPLC analysis on a CHIRALCEL AD column, λ = 254 nm. Flow rate = 1.0 mL/min; Retention times in 10% 2-propanol in hexanes were 44.9 min (major) and 49.4 min (minor). m.p.: 187–188 °C; ${\left[\alpha \right]}_{D}^{25}$ = +25.2° (*c* = 11.9 mg, CHCl_3_); IR (thin film, cm^−1^) 3591 (br), 3210 (w), 2973 (s), 2933 (s), 2874 (s), 2806 (s), 1676 (s), 1490 (s), 1445 (s), 1415 (s), 1383 (s), 1297 (m), 1077 (s), 1044 (s), 935 (s), 846 (s); ^1^H-NMR (400 MHz, CDCl_3_) δ 7.28 (1H, dd, *J =* 2.8 Hz, *J =* 2.8 Hz), 7.15 (1H, s), 6.97 (1H, t, *J =* 2.8 Hz), 6.75 (1H, dt, *J =* 8.8 Hz, *J =* 2.7 Hz), 6.66 (1H, d, *J =* 2.4 Hz), 6.50 (1H, dd, *J =* 2.8 Hz, *J =* 2.4 Hz), 6.01 (1H, dd, *J =* 2.8 Hz, *J =* 2.4 Hz), 4.86 (1H, d, *J =* 11.6 Hz), 3.95 (3H, d, *J =* 2.4 Hz), 3.89 (3H, *J =* 2.4 Hz), 3.49 (1H, dd, *J =* 2.4 Hz, *J =* 2.4 Hz), 3.24–3.16 (5H, m), 2.95–2.91 (2H, m), 2.73–2.69 (2H, m); ^13^C-NMR (100 MHz, CDCl_3_) δ 150.8, 148.9, 148.3, 133.0, 130.6, 129.9, 129.3, 124.7, 122.5, 122.2, 117.5, 115.6, 110.3, 109.0, 68.0, 67.8, 56.2, 56.2, 49.4, 48.9. MS (ESI): calcd *m*/*z* for C_22_H_24_Cl_2_N_2_O_3_ (M^+^) 434.12, found: 435.04 [M + H]^+^. Anal. Calcd for C_22_H_24_Cl_2_N_2_O_3_: C, 60.70; H, 5.56; N, 6.43. Found: C, 60.57; H, 5.84; N, 6.36.

*(1S,2S)-2-[4-(2-Chloro-phenyl)-piperazin-1-yl]-6,7-dimethoxy-1,2-dihydro-naphthalen-1-ol* (**3f**). Prepared according to general procedure. **3f** was obtained as a white solid (50 mg, 51%) by flash chromatography (ethyl acetate: petroleum ether = 1:1, *v*/*v*). R_f_ = 0.22 on silica gel (ethyl acetate: petroleum ether = 1:1, *v*/*v*). The *ee* was determined to be 43% using HPLC analysis on a CHIRALCEL AD column, λ = 254 nm. Flow rate = 1.0 mL/min; Retention times in 2% 2-propanol in hexanes were 114.4 min (minor) and 125.3 min (major). m.p.: 140–141 °C; ${\left[\alpha \right]}_{D}^{25}$ = +75.3° (*c* = 38.3 mg, CHCl_3_); IR (thin film, cm^−1^) 3591 (br), 3211 (w), 2975 (s), 2934 (s), 2875 (s), 2806 (s), 1678 (w), 1490 (s), 1445 (s), 1378 (s), 1297 (m), 1108 (s), 1077 (s), 918 (s), 846 (s); ^1^H-NMR (400 MHz, CDCl_3_) δ 7.49 (1H, dd, *J =* 2.4 Hz, *J =* 2.4 Hz), 7.39–7.33 (1H, m), 7.27 (1H, s), 7.18 (1H, dd, *J =* 2.4 Hz, *J =* 2.4 Hz), 6.95 (1H, t, *J =* 7.2 Hz), 6.77 (1H, s), 6.60 (1H, dd, *J =* 2.4 Hz, *J =* 2.4 Hz), 6.21 (1H, dd, *J =* 2.4 Hz, *J =* 2.4 Hz), 4.98 (1H, d, *J =* 11.6 Hz), 4.07 (3H, s), 3.99 (3H, s), 3.61 (1H, d, *J =* 11.6 Hz), 3.24 (4H, br), 3.15–2.95 (2H, m), 2.83–2.64 (2H, m); ^13^C-NMR (100 MHz, CDCl_3_) δ 149.4, 148.9, 148.3, 130.9, 130.1, 139.1, 129.0, 127.8, 124.9, 124.0, 122.9, 120.6, 110.3, 108.9, 68.0, 56.3, 56.3, 52.0, 49.4, 29.9. MS (ESI): calcd *m*/*z* for C_22_H_25_ClN_2_O_3_ (M^+^) 400.16, found: 401.12 [M + H]^+^. Anal. Calcd for C_22_H_25_ClN_2_O_3_: C, 65.91; H, 6.29; N, 6.99. Found: C, 65.79; H, 6.48; N, 6.76.

*(1S,2S)-6,7-Dimethoxy-2-(4-o-tolyl-piperazin-1-yl]-1,2-dihydro-naphthalen-1-ol* (**3g**). Prepared according to general procedure. **3g** was obtained as a white solid (71 mg, 76%) by flash chromatography (ethyl acetate: petroleum ether = 1:2, *v*/*v*). R_f_ = 0.18 on silica gel (ethyl acetate: petroleum ether = 1:2, *v*/*v*). The *ee* was determined to be 38% using HPLC analysis on a CHIRALCEL AD column, λ = 254 nm. Flow rate = 1.0 mL/min; Retention times in 2% 2-propanol in hexanes were 79.8 min (minor) and 85.1 min (major). m.p.: 120–121 °C; ${\left[\alpha \right]}_{D}^{25}$ = +40.1° (*c* = 38.9 mg, CHCl_3_); IR (thin film, cm^−1^) 3693 (br), 3218 (w), 2980 (s), 2869 (s), 1681 (w), 1498 (s), 1445 (s), 1381 (s), 1351 (s), 1297 (m), 1142 (s), 935 (s), 846 (s);. ^1^H-NMR (400 MHz, CDCl_3_) δ 7.21–7.18 (3H, m), 7.06–6.99 (2H, m), 6.67 (1H, s), 6.50 (1H, dd, *J =* 2.4 Hz, *J =* 2.4 Hz), 6.12 (1H, dd, *J =* 2.4 Hz, *J =* 2.4 Hz), 4.88 (1H, d, *J =* 11.6 Hz), 3.96 (3H, s), 3.89 (3H, s), 3.49 (1H, dt, *J =* 6.8 Hz, *J =* 2.4 Hz), 3.27 (1H, br), 3.02–2.96 (6H, m), 2.75–2.71 (2H, m), 2.34 (3H, s); ^13^C-NMR (100 MHz, CDCl_3_) δ 151.6, 148.8, 148.2, 132.7, 131.3, 130.2, 128.9, 126.8, 124.8, 123.4, 123.0, 119.2, 110.2, 108.8, 68.0, 68.0, 56.3, 56.2, 52.4, 49.7, 18.1. MS (ESI): calcd *m*/*z* for C_23_H_28_N_2_O_3_ (M^+^) 380.21, found: 381.15 [M + H]^+^. Anal. Calcd for C_23_H_28_N_2_O_3_: C, 72.60; H, 7.42; N, 7.36. Found: C, 72.30; H, 7.64; N, 7.18.

*(1S,2S)-2-[4-(2,5-Dimethyl-phenyl)-piperazin-1-yl]-6,7-dimethoxy-1,2-di-hydro-naphthalen-1-ol* (**3h**). Prepared according to general procedure. **3h** was obtained as a white solid (83 mg, 86%) by flash chromatography (ethyl acetate: petroleum ether = 1:2, *v*/*v*). R_f_ = 0.29 on silica gel (ethyl acetate: petroleum ether = 1:2, *v*/*v*). The *ee* was determined to be 35% using HPLC analysis on a CHIRALCEL AD column, λ = 254 nm. Flow rate = 1.0 mL/min; Retention times in 2% 2-propanol in hexanes were 63.6 min (minor) and 65.1 min (major). m.p.: 138–139 °C; ${\left[\alpha \right]}_{D}^{25}$ = +73.2° (*c* = 68.5 mg, CHCl_3_); IR (thin film, cm^−1^) 3592 (br), 3211 (w), 2975 (s), 2934 (s), 2863 (s), 2805 (s), 1677 (w), 1490 (s), 1444 (s), 1381 (s), 1297 (m), 1119 (s), 935 (s), 846 (s); ^1^H-NMR (400 MHz, CDCl_3_) δ 7.19 (1H, s), 7.08 (1H, d, *J =* 7.6 Hz), 6.87 (1H, s), 6.83 (1H, d, *J =* 7.6 Hz), 6.67 (1H, s), 6.50 (1H, dd, *J =* 2.0 Hz, *J =* 2.0 Hz), 6.12 (1H, dd, *J =* 2.0 Hz, *J =* 2.0 Hz), 4.89 (1H, d, *J =* 11.6 Hz), 3.96 (3H, s), 3.90 (3H, s), 3.49 (1H, d, *J =* 12.0 Hz), 3.37 (1H, br), 3.04–2.95 (6H, m), 2.72 (2H, t, *J =* 6.8 Hz), 2.33 (3H, s), 2.29 (3H, s); ^13^C-NMR (100 MHz, CDCl_3_) δ 151.4, 148.8, 148.2, 136.3, 131.1, 130.2, 129.4, 129.0, 124.8, 124.0, 123.0, 119.9, 110.2, 108.8, 68.0, 67.9, 56.2, 56.2, 52.4, 49.7, 21.3, 17.6. MS (ESI): calcd *m*/*z* for C_24_H_30_N_2_O_3_ (M^+^) 394.23, found: 395.15 [M + H]^+^. Anal. Calcd for C_24_H_30_N_2_O_3_: C, 73.07; H, 7.66; N, 7.10. Found: C, 73.16; H, 7.91; N, 6.95.

*(1S,2S)-2-[4-(3,4-Dimethyl-phenyl)-piperazin-1-yl]-6,7-dimethoxy-1,2-di-hydro-naphthalen-1-ol* (**3i**). Prepared according to general procedure. **3i** was obtained as a white solid (78 mg, 81%) by flash chromatography (ethyl acetate: petroleum ether = 1:2, *v*/*v*). R_f_ = 0.16 on silica gel (ethyl acetate: petroleum ether = 1:2, *v*/*v*). The *ee* was determined to be 45% using HPLC analysis on a CHIRALCEL AD column, λ = 254 nm. Flow rate = 1.0 mL/min; Retention times in 2% 2-propanol in hexanes were 21.6 min (major) and 22.6 min (minor). m.p.: 145–146 °C; ${\left[\alpha \right]}_{D}^{25}$ = +95.6° (*c* = 29.3 mg, CHCl_3_); IR (thin film, cm^−1^) 3695 (br), 3219 (w), 2978 (s), 2936 (s), 2867 (s), 1699 (w), 1490 (w), 1445 (s), 1383 (s), 1351 (s), 1297 (m), 1124 (s), 1077 (s), 935 (s), 846 (s); ^1^H-NMR (400 MHz, CDCl_3_) δ 7.16 (1H, s), 7.04 (1H, d, *J =* 8.4 Hz), 6.78 (1H, d, *J =* 2.4 Hz), 6.77 (1H, dd, *J =* 2.4 Hz, *J =* 2.4 Hz), 6.66 (1H, s), 6.48 (1H, dd, *J =* 2.4 Hz, *J =* 2.4 Hz), 4.88 (1H, d, *J =* 6.8 Hz), 3.95 (3H, s), 3.89 (3H, s), 3.48 (1H, dt, *J =* 6.8 Hz, *J =* 2.4 Hz), 3.20 (1H, br), 3.19–3.16 (4H, m), 2.95–2.87 (2H, m), 2.75–2.69 (2H, m), 2.25 (3H, s), 2.21 (3H, s); ^13^C-NMR (100 MHz, CDCl_3_) δ 149.7, 148.7, 148.1, 137.2, 130.3, 130.0, 129.0, 128.4, 124.7, 122.6, 118.4, 114.1, 110.1, 108.8, 67.8, 67.8, 56.2, 56.2, 50.6, 49.2, 20.3, 18.9. MS (ESI): calcd *m*/*z* for C_24_H_30_N_2_O_3_ (M^+^) 394.23, found: 395.15 [M + H]^+^. Anal. Calcd for C_24_H_30_N_2_O_3_: C, 73.07; H, 7.66; N, 7.10. Found: C, 73.30; H, 8.16; N, 6.74.

## 4. Conclusions

In conclusion, we have developed the iridium-catalyzed asymmetric ring-opening of oxabicyclic alkenes with *N*-substituted piperazine nucleophiles. It may provide an efficient and practical access to optically pure *trans*-2-*N*-substituted piperazine 1,2-dihydronaphthalen-1-ols in high yields and moderate enantioselectivities. Catalyst systems consisting of four different kinds of chiral bisphosphine ligands with [Ir(COD)Cl]_2_ to form complexes as iridium catalysts were investigated, and (*S*)-*p*-Tol-BINAP ligand was found to give high yields and moderate enantioselectivities. Further investigation to expand the scope of the reactions as well as the study of the iridium-catalyzed asymmetric ring-opening reactions of oxabicyclic alkenes with other nucleophiles are in progress in our laboratory.

## Supplementary Materials

The full characterization data for all compounds **1a**–**1b**, **2a**–**2x** and **3a**–**3i**, including optical rotations, ^1^H-NMR and ^13^C-NMR, MS, elemental analysis and X-ray structure data for compound **2i** in CIF format**,** and HPLC conditions and spectra of compounds **2a**, **2c**, **2g**–**2h**, **2i**–**2k**, **2m**–**2o**, **2q**–**2r**, **2u**–**2x** and **3b** are provided in the Supplementary materials at: http://www.mdpi.com/1420-3049/20/12/19748/s1.

## References

[1] Lautens M., Fagnou K., Taylor M. (2000). Rhodium-catalyzed asymmetric ring opening of oxabicyclic alkenes with phenols. Org. Lett..

[2] Lautens M., Fagnou K. (2001). Effects of halide ligands and protic additives on enantioselectivity and reactivity in rhodium-catalyzed asymmetric ring-opening reactions. J. Am. Chem. Soc..

[3] Lautens M., Fagnou K., Taylor M., Rovis T. (2001). Rhodium-catalyzed asymmetric ring opening of oxabicyclic alkenes with heteroatom nucleophiles. J. Organomet. Chem..

[4] Lautens M., Fagnou K. (2001). Rhodium-catalysed asymmetric ring opening reactions with carboxylate nucleophiles. Tetrahedron.

[5] Lautens M., Fagnou K., Zunic V. (2002). An expedient enantioselective route to diaminotetralins: Application in the preparation of analgesic compounds. Org. Lett..

[6] Lautens M., Dockendorff C., Fagnou K., Malicki A. (2002). Rhodium-catalyzed asymmetric ring opening of oxabicyclic alkenes with organoboronic acids. Org. Lett..

[7] Lautens M., Fagnou K., Yang D.-Q. (2003). Rhodium-catalyzed asymmetric ring opening reactions of oxabicyclic alkenes: Application of halide effects in the development of a general process. J. Am. Chem. Soc..

[8] Lautens M., Fagnou K. (2004). Rhodium-catalyzed asymmetric ring opening reactions of oxabicyclic alkenes: Catalyst and substrate studies leading to a mechanistic working model. Proc. Natl. Acad. Sci. USA.

[9] Leong P., Lautens M. (2004). Rhodium-catalyzed asymmetric ring opening of oxabicyclic alkenes with sulfur nucleophiles. J. Org. Chem..

[10] Cho Y.-H., Zunic V., Senboku H., Olsen M., Lautens M. (2006). Rhodium-catalyzed ring-opening reactions of *N*-Boc-azabenzonorbornadienes with amine nucleophiles. J. Am. Chem. Soc..

[11] Nishimura T., Tsurumaki E., Kawamoto T., Guo X.-X., Hayashi T. (2008). Rhodium-catalyzed asymmetric ring-opening alkynylation of azabenzonorbornadienes. Org. Lett..

[12] Long Y.-H., Zhao S.-Q., Zeng H.-P., Yang D.-Q. (2010). Highly efficient rhodium-catalyzed asymmetric ring-opening reactions of oxabenzonorbornadiene with amine nucleophiles. Catal. Lett..

[13] Tsui G.C., Lautens M. (2012). Rhodium(I)-catalyzed domino asymmetric ring opening/enantioselective isomerization of oxabicyclic alkenes with water. Angew. Chem. Int. Ed..

[14] Zhu J., Tsui G.C., Lautens M. (2012). Rhodium-catalyzed enantioselective nucleophilic fluorination: Ring opening of oxabicyclic alkenes. Angew. Chem. Int. Ed..

[15] Tsui G.C., Ninnemann N.M., Hosotani A., Lautens M. (2013). Expedient synthesis of chiral oxazolidinone scaffolds via rhodium-catalyzed asymmetric ring-opening with sodium cyanate. Org. Lett..

[16] Zhang L., Le C.M., Lautens M. (2014). The use of silyl ketene acetals and enol ethers in the catalytic enantioselective alkylative ring opening of oxa/aza bicyclic alkenes. Angew. Chem. Int. Ed..

[17] Luo R.-S., Xie L., Liao J.-H., Xin H., Chan A.S.C. (2014). Tunable chiral monophosphines as ligands in enantioselective rhodium-catalyzed ring-opening of oxabenzonorbornadienes with amines. Tetrahedron Asymmetry.

[18] Bertozzi F., Pineschi M., Macchia F., Arnold L.A., Minnaard A.J., Feringa B.L. (2002). Copper phosphoramidite catalyzed enantioselective ring-opening of oxabicyclic alkenes: Remarkable reversal of stereocontrol. Org. Lett..

[19] Zhang W., Wang L.-X., Shi W.-J., Zhou Q.-L. (2005). Copper-catalyzed asymmetric ring opening of oxabicyclic alkenes with Grignard reagents. J. Org. Chem..

[20] Zhang W., Zhu S.-F., Qiao X.-C., Zhou Q.-L. (2008). Highly enantioselective copper-catalyzed ring opening of oxabicyclic alkenes with Grignard reagents. Chem. Asian J..

[21] Millet R., Gremaud L., Bernardez T., Palais L., Alexakis A. (2009). Electromagnetic composite films based on polypyrrole hydro-sponge and Fe_3_O_4_ ferrofluid. Synthesis.

[22] Millet R., Bernardez T., Palais L., Alexakis A. (2009). Copper-catalyzed desymmetrization of oxabenzonorbornadienes with aluminium reagents. Tetrahedron Lett..

[23] Bos P.H., Rudolph A., Pérez M., Fañanás-Mastral M., Harutyunyan S.R., Feringa B.L. (2012). Copper-catalyzed asymmetric ring opening of oxabicyclic alkenes with organolithium reagents. Chem. Commun..

[24] Lautens M., Renaud J.L., Hiebert S. (2000). Palladium-catalyzed enantioselective alkylative ring opening. J. Am. Chem. Soc..

[25] Lautens M., Dockendorff C. (2003). Palladium(II) catalyst systems for the addition of boronic acids to bicyclic alkenes: New scope and reactivity. Org. Lett..

[26] Cabrera S., Arrayás R.G., Carretero J.C. (2004). Cationic planar chiral palladium P,S complexes as highly efficient catalysts in the enantioselective ring opening of oxa- and azabicyclic alkenes. Angew. Chem. Int. Ed..

[27] Li M., Yan X.-X., Hong W., Zhu X.-Z., Cao B.-X., Sun J., Hou X.-L. (2004). Palladium-catalyzed enantioselective ring opening of oxabicyclic alkenes with organozinc halides. Org. Lett..

[28] Lautens M., Hiebert S. (2004). Scope of palladium-catalyzed alkylative ring opening. J. Am. Chem. Soc..

[29] Cabrera S., Arrayás R.G., Alonso I., Carretero J.C. (2005). Fesulphos-palladium(II) complexes as well-defined catalysts for enantioselective ring opening of meso heterobicyclic alkenes with organozinc reagents. J. Am. Chem. Soc..

[30] Zhang T.-K., Mo D.-L., Dai L.-X., Hou X.-L. (2008). Asymmetric ring-opening reaction of oxabicyclic alkenes with aryl boronic acids catalyzed by P-containing palladacycles. Org. Lett..

[31] Ogura T., Yoshida K., Yanagisawa A., Imamoto T. (2009). Optically active dinuclear palladium complexes containing a Pd−Pd bond: Preparation and enantioinduction ability in asymmetric ring-opening reactions. Org. Lett..

[32] Endo K., Tanaka K., Ogawa M., Shibata T. (2011). Multinuclear Pd/Zn complex-catalyzed asymmetric alkylative ring-opening reaction of oxabicyclic alkenes. Org. Lett..

[33] Huang X.-J., Mo D.-L., Ding C.-H., Hou X.-L. (2011). Chiral P-containing palladacycle-catalyzed asymmetric ring-opening reactions of oxabicyclic alkenes with alkenyl boronic acids. Synlett.

[34] Mo D.-L., Chen B., Ding C.-H., Dai L.-X., Ge G.-C., Hou X.-L. (2013). Switch of addition and ring-opening reactions of oxabicyclic alkenes with terminal alkynes by sp^2^-C, P- and sp^3^-C, P-palladacycle catalysis. Organometallics.

[35] Zhang Z.-M., Ouyang L., Wu W.-Q., Li J.-X., Zhang Z.-C., Jiang H.-F. (2014). Palladium-catalyzed intermolecular oxyvinylcyclization of alkenes with alkynes: An approach to 3-methylene γ-lactones and tetrahydrofurans. J. Org. Chem..

[36] Lautens M., Chiu P., Ma S., Rovis T. (1995). Nickel-catalyzed hydroalumination of oxabicyclic alkenes. Ligand effects on the regio- and enantioselectivity. J. Am. Chem. Soc..

[37] Lautens M., Rovis T. (1997). A new route to the enantioselective synthesis of cycloheptenols. temperature effects in the asymmetric reductive ring opening of [3.2.1] oxabicycloalkenes. J. Am. Chem. Soc..

[38] Lautens M., Rovis T. (1997). General strategy toward the tetrahydronaphthalene skeleton. An expedient total synthesis of sertraline. J. Org. Chem..

[39] Lautens M., Rovis T. (1998). Scope of nickel catalyzed asymmetric reductive ring opening reaction. Synthesis of enantiomerically enriched cyclohexenols. Tetrahedron.

[40] Li L.-P., Rayabarapu D.K., Nandi M., Cheng C.-H. (2003). Asymmetric reductive ring-opening of bicyclic olefins catalyzed by palladium and nickel complexes. Org. Lett..

[41] Mannathan S., Cheng C.-H. (2014). Nickel-catalyzed regio- and stereoselective reductive coupling of oxa- and azabicyclic alkenes with enones and electron-rich alkynes. Adv. Synth. Catal..

[42] Zeng Z.-Y., Yang D.-Q., Long Y.-H., Pan X.-J., Huang G.-B., Zuo X.-J., Zhou W. (2014). Nickel-catalyzed asymmetric ring opening of oxabenzonorbornadienes with arylboronic acids. J. Org. Chem..

[43] Millward D.B., Sammis G., Waymouth R.M. (2000). Ring-opening reactions of oxabicyclic alkene compounds: Enantioselective hydride and ethyl additions catalyzed by group 4 metals. J. Org. Chem..

[44] Nakamura M., Matsuo K., Znoue T., Nakamura E. (2003). Iron-catalyzed regio- and stereoselective ring opening of [2.2.1]- and [3.2.1]oxabicyclic alkenes with a Grignard reagent. Org. Lett..

[45] Villeneuve K., Tam W. (2006). Ruthenium-catalyzed isomerization of oxa/azabicyclic alkenes: An expedient route for the synthesis of 1,2-naphthalene oxides and imines. J. Am. Chem. Soc..

[46] Burton R.R., Tam W. (2007). Ruthenium(II)-catalyzed cyclization of azabenzonorbornadienes with alkynes. Org. Lett..

[47] Carreras J., Avenoza A., Busto J.H., Peregrina J.M. (2007). Regioselective ring-opening metathesis-cross metathesis of bridgehead-substituted 7-azanorbornene. Org. Lett..

[48] Cortez G.A., Baxter C.A., Schrock R.R., Hoveyda A.H. (2007). Comparison of Ru- and Mo-based chiral olefin metathesis catalysts. Complementarity in asymmetric ring-opening/cross-metathesis reactions of oxa- and azabicycles. Org. Lett..

[49] Villeneuve K., Tam W. (2007). Construction of isochromenes via a ruthenium-catalyzed reaction of oxabenzonorbornenes with propargylic alcohols. Organometallics.

[50] Machin B.P., Howell J., Mandel J., Blanchard N., Tam W. (2009). Ruthenium-catalyzed nucleophilic ring-opening reactions of a 3-aza-2-oxabicyclo[2.2.1]hept-5-ene with alcohols. Org. Lett..

[51] Ballantine M., Menard M.L., Tam W. (2009). Isomerization of 7-oxabenzonorbornadienes into naphthols catalyzed by [RuCl_2_(CO)_3_]_2_. J. Org. Chem..

[52] Tenaglia A., Marc S., Giordano L., Riggi I.D. (2011). Ruthenium-catalyzed coupling of oxabenzonorbornadienes with alkynes bearing a propargylic oxygen atom: Access to stereodefined benzonorcaradienes. Angew. Chem. Int. Ed..

[53] Pan X.-J., Huang G.-B., Long Y.-H., Zuo X.-J., Xu X., Gu F.-L., Yang D.-Q. (2014). Platinum(II)-catalyzed asymmetric ring-opening addition of arylboronic acids to oxabenzonorbornadienes. J. Org. Chem..

[54] Yang D.-Q., Liang N. (2014). Platinum-catalyzed *anti*-stereocontrolled ring-opening of oxabicyclic alkenes with Grignard reagents. Org. Biomol. Chem..

[55] Meng L., Yang W., Pan X.-J., Tao M., Cheng G., Wang S.-Y., Zeng H.-P., Long Y.-H., Yang D.-Q. (2015). Platinum-catalyzed asymmetric ring-opening reactions of oxabenzonorbornadienes with phenols. J. Org. Chem..

[56] Rayabarapu D.K., Chiou C.-F., Cheng C.-H. (2002). Highly stereoselective ring-opening addition of terminal acetylenes to bicyclic olefins catalyzed by nickel complexes. Org. Lett..

[57] Li L.-P., Rayabarapu D.K., Nandi M., Cheng C.-H. (2003). Asymmetric reductive ring-opening of bicyclic olefins catalyzed by palladium and nickel complexes. Org. Lett..

[58] Wu M.-S., Rayabarapu D.K., Cheng C.-H. (2004). Nickel-catalyzed addition of alkenylzirconium reagents to bicyclic olefins: A highly regio- and stereoselective ring-opening reaction. J. Org. Chem..

[59] Wu M.-S., Jeganmohan M., Cheng C.-H. (2005). A highly regio- and stereoselective nickel-catalyzed ring-opening reaction of alkyl- and allylzirconium reagents to 7-oxabenzonorbornadienes. J. Org. Chem..

[60] Arrayas R.G., Cabrera S.J., Carretero J.C. (2003). Copper-catalyzed *anti*-stereocontrolled ring opening of oxabicyclic alkenes with Grignard reagents. Org. Lett..

[61] Arrayas R.G., Cabrera S., Carretero J.C. (2005). Copper-catalyzed *anti*-stereocontrolled ring-opening of azabicyclic alkenes with Grignard reagents. Org. Lett..

[62] Yang D.-Q., Long Y.-H., Wang H., Zhang Z.-M. (2008). Iridium-catalyzed asymmetric ring-opening reactions of *N*-Boc-azabenzonorbornadiene with secondary amine nucleophiles. Org. Lett..

[63] Yang D.-Q., Hu P., Long Y.-H., Wu Y.-J., Zeng H.-P., Wang H., Zuo X.-J. (2009). Iridium-catalyzed asymmetric ring-opening reactions of oxabicyclic alkenes with secondary amine nucleophiles. Beilstein J. Org. Chem..

[64] Long Y.-H., Yang D.-Q., Zhang Z.-M., Wu Y.-J., Zeng H.-P., Chen Y. (2010). Iridium-catalyzed asymmetric ring opening of azabicyclic alkenes by amines. J. Org. Chem..

[65] Yang D.-Q., Long Y.-H., Wu Y.-J., Zuo X.-J., Tu Q.-Q., Fang S., Jiang L.-S., Wang S.-Y., Li C.-R. (2010). Iridium-catalyzed *anti*-stereocontrolled asymmetric ring opening of azabicyclic alkenes with primary aromatic amines. Organometallics.

[66] Yang D.-Q., Long Y.-H., Zhang J.-F., Zeng H.-P., Wang S.-Y., Li C.-R. (2010). Iridium-catalyzed asymmetric ring-opening reactions of oxabenzonorbornadienes with amine nucleophiles. Organometallics.

[67] Fang S., Liang X.-L., Long Y.-H., Li X.-L., Yang D.-Q., Wang S.-Y., Li C.-R. (2012). Iridium-catalyzed asymmetric ring-opening of azabicyclic alkenes with phenols. Organometallics.

[68] Cheng H.-C., Yang D.-Q. (2012). Iridium-catalyzed asymmetric ring-opening of oxabenzonorbornadienes with phenols. J. Org. Chem..

[69] Li S.-F., Chen H.-L., Yang Q.-J., Yu L., Fan C.-L., Zhou Y.-Y., Wang J., Fan B.-M. (2013). Iridium/NMDPP catalyzed asymmetric ring-opening reaction of oxabenzonorbornadienes with phenolic or naphtholic nucleophiles. Asian J. Org. Chem..

[70] Yang D.-Q., Xia J.-Y., Long Y.-H., Zeng Z.-Y., Zuo X.-J., Wang S.-Y., Li C.-R. (2013). Iridium-catalyzed asymmetric ring-opening of azabicyclic alkenes with alcohols. Org. Biomol. Chem..

[71] Luo R.-S., Liao J.-H., Xie L., Tang W.-J., Chan A.S.C. (2013). Asymmetric ring-opening of oxabenzonorbornadiene with amines promoted by a chiral iridium-monophosphine catalyst. Chem. Commun..

[72] Long Y.-H., Li X.-L., Pan X.-J., Ding D.-D., Xu X., Zuo X.-J., Yang D.-Q., Wang S.-Y., Li C.-R. (2014). Iridium-catalyzed asymmetric ring-opening of oxabicyclic alkenes with carboxylic acids. Catal. Lett..

[73] Long Y.-H., Wang W.-L., Yang D.-Q., Jiang H., Chen K.-X., Fang Y.-L. (2014). Iridium-catalyzed asymmetric ring-opening reactions of azabenzonorbornadiene with carboxylic acid nucleophiles. Mol. Divers..

[74] Lu L., Zhou Y.Y., Xu X., Li S.F., Xu J.B., Fan B.M., Lin C.Y., Bian Z.X., Chan A.S.C. (2014). Asymmetric ring opening reaction of oxabenzonorbornadienes with amines promoted by iridium/NMDPP complex. Tetrahedron Lett..

[75] Lautens M., Schmid G.A., Chau A. (2002). Remote electronic effects in the rhodium-catalyzed nucleophilic ring opening of oxabenzonorbornadienes. J. Org. Chem..

