# Supplementary material for: Iridium-Catalyzed Asymmetric Ring-Opening of Oxabenzonorbornadienes with N-Substituted Piperazine Nucleophiles
Source: Molecules. 2015 Nov 27;20(12):21103–24. doi: 10.3390/molecules201219748 (PMC6332042; doi:10.3390/molecules201219748)
Supplement: Supplementary file 1 [file molecules-20-19748-s001.pdf]

Supplementary Materials: Iridium-Catalyzed Asymmetric Ring-Opening of Oxabenzonorbornadienes with *N*-Substituted Piperazine Nucleophiles

Wen Yang, Renshi Luo, and Dingqiao Yang

1. Crystal Structure and Data of (1*S*,2*S*)-2-[4-(4-fluoro-phenyl)-piperazin-1-yl]-1,2-dihydro-naphthalen-1-ol (2i) (CCDC 1415336)

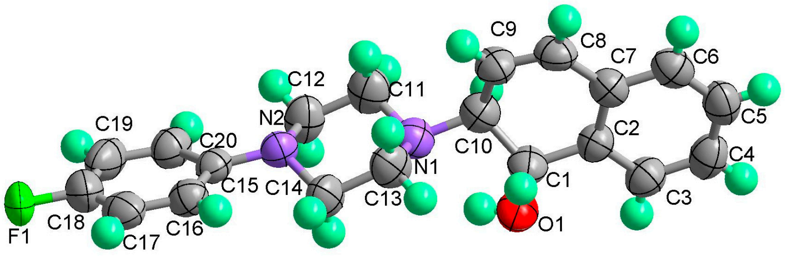

Figure S1. Crystal structure of **2i**.

Table S1. Crystal data and structure refinement for **2i**.

Identification Code	2i
Empirical formula	$C_{20}H_{21}FN_2O$
Formula weight	324.39
Temperature	296 (2) K
Wavelength	0.71073 A
Crystal system, space group	Orthorhombic, P 21 21 21
	a = 9.572 (3) A alpha = 90 deg.
Unit cell dimensions	*b* = 10.034 (3) A beta = 90 deg.
	c = 17.732 (5) A gamma = 90 deg.
Volume	1703.1 (9) A^3
Z, Calculated density	4, 1.265 Mg/m^3
Absorption coefficient	0.086 mm^{-1}
F (000)	688
Crystal size	0.22 × 0.20 × 0.18 mm
Theta range for data collection	2.30 to 26.40 deg.
Limiting indices	$-11 \leq 11$, $-12 \leq k \leq 12$, $-22 \leq l \leq 11$
Reflections collected/unique	9291/3432 [R(int) = 0.0357]
Completeness to theta = 26.40	98.5%
Refinement method	Full-matrix least-squares on F^2
Data/restraints/parameters	3432/1/218
Goodness-of-fit on F^2	1.037
Final R indices [I > 2sigma(I)]	R1 = 0.0583, wR2 = 0.1588
R indices (all data)	R1 = 0.1067, wR2 = 0.1882
Absolute structure parameter	1 (2)
Largest diff. peak and hole	0.357 and −0.205 e·A^{-3}

Table S2. Atomic coordinates ($\times 10^4$) and equivalent isotropic displacement parameters ($A^2 \times 10^3$) for **2i**. U (eq) is defined as one third of the trace of the orthogonalized Uij tensor.

x	y	z	U (eq)	
C (1)	−816 (5)	6098 (4)	5364 (2)	82 (1)
C (2)	413 (5)	6733 (4)	5210 (2)	83 (1)
C (3)	845 (4)	6884 (4)	4470 (2)	76 (1)
C (4)	21 (4)	6413 (3)	3877 (2)	65 (1)
C (5)	−1244 (4)	5778 (3)	4036 (2)	67 (1)
C (6)	−1654 (4)	5616 (4)	4786 (2)	78 (1)
C (7)	482 (4)	6457 (4)	3074 (2)	77 (1)
C (8)	−675 (4)	6622 (4)	2530 (2)	79 (1)
C (9)	−1806 (4)	5605 (4)	2699 (2)	75 (1)
C (10)	−2083 (4)	5292 (4)	3404 (2)	76 (1)
C (11)	309 (4)	5414 (4)	1437 (2)	86 (1)
C (12)	952 (4)	5605 (4)	663 (2)	84 (1)
C (13)	−530 (5)	7514 (4)	481 (2)	86 (1)
C (14)	−1147 (5)	7275 (4)	1241 (2)	89 (1)
C (15)	300 (3)	6263 (3)	−625 (2)	61 (1)
C (16)	860 (4)	5116 (4)	−963 (2)	71 (1)
C (17)	1098 (4)	5039 (5)	−1726 (2)	86 (1)
C (18)	762 (5)	6082 (6)	−2162 (2)	93 (1)
C (19)	216 (4)	7238 (5)	−1873 (2)	98 (1)
C (20)	7 (4)	7331 (4)	−1098 (2)	77 (1)
F (1)	1000 (4)	6028 (4)	−2928 (1)	150 (1)
N (1)	−144 (3)	6681 (3)	1753 (2)	77 (1)
N (2)	−23 (3)	6259 (3)	147 (2)	62 (1)
O (1)	1578 (2)	7409 (3)	2966 (1)	80 (1)

Symmetry transformations used to generate equivalent atoms:

Table S3. Bond lengths [Å] and angles [deg] for **2i**.

Bonding Lengthes	Å
C(1)-C(2)	1.365 (6)
C(1)-C(6)	1.389 (5)
C(2)-C(3)	1.384 (5)
C(3)-C(4)	1.397 (5)
C(4)-C(5)	1.397 (5)
C(4)-C(7)	1.492 (5)
C(5)-C(6)	1.396 (5)
C(5)-C(10)	1.462 (5)
C(7)-O(1)	1.432 (4)
C(7)-C(8)	1.478 (5)
C(8)-N(1)	1.469 (4)
C(8)-C(9)	1.518 (5)
C(9)-C(10)	1.316 (5)
C(11)-N(1)	1.455 (5)
C(11)-C(12)	1.515 (5)
C(12)-N(2)	1.462 (4)
C(13)-N(2)	1.474 (5)
C(13)-C(14)	1.491 (5)
C(14)-N(1)	1.449 (5)

Table S3. *Cont.*

Bonding Lengthes	Å
C(15)-C(20)	1.390 (5)
C(15)-N(2)	1.403 (4)
C(15)-C(16)	1.404 (5)
C(16)-C(17)	1.375 (5)
C(17)-C(18)	1.340 (6)
C(18)-C(19)	1.372 (6)
C(18)-F(1)	1.378 (5)
C(19)-C(20)	1.392 (5)
C(2)-C(1)-C(6)	120.8 (4)
C(1)-C(2)-C(3)	119.9 (4)
C(2)-C(3)-C(4)	120.5 (4)
C(3)-C(4)-C(5)	119.5 (3)
C(3)-C(4)-C(7)	122.8 (3)
C(5)-C(4)-C(7)	117.5 (3)
C(6)-C(5)-C(4)	119.2 (3)
C(6)-C(5)-C(10)	122.5 (3)
C(4)-C(5)-C(10)	118.3 (3)
C(1)-C(6)-C(5)	120.0 (4)
O(1)-C(7)-C(8)	112.8 (3)
O(1)-C(7)-C(4)	111.4 (3)
C(8)-C(7)-C(4)	113.9 (3)
N(1)-C(8)-C(7)	110.9 (3)
N(1)-C(8)-C(9)	117.3 (3)
C(7)-C(8)-C(9)	109.3 (3)
C(10)-C(9)-C(8)	119.4 (3)
C(9)-C(10)-C(5)	122.6 (3)
N(1)-C(11)-C(12)	111.1 (3)
N(2)-C(12)-C(11)	111.4 (3)
N(2)-C(13)-C(14)	110.8 (3)
N(1)-C(14)-C(13)	111.7 (3)
C(20)-C(15)-N(2)	123.2 (3)
C(20)-C(15)-C(16)	116.8 (3)
N(2)-C(15)-C(16)	119.9 (3)
C(17)-C(16)-C(15)	122.0 (4)
C(18)-C(17)-C(16)	119.0 (4)
C(17)-C(18)-C(19)	122.4 (4)
C(17)-C(18)-F(1)	119.9 (5)
C(19)-C(18)-F(1)	117.7 (5)
C(18)-C(19)-C(20)	118.7 (4)
C(19)-C(20)-C(15)	121.1 (4)
C(14)-N(1)-C(11)	108.4 (3)
C(14)-N(1)-C(8)	112.0 (3)
C(11)-N(1)-C(8)	115.4 (3)
C(15)-N(2)-C(12)	118.1 (3)
C(15)-N(2)-C(13)	117.5 (3)
C(12)-N(2)-C(13)	110.0 (3)

Symmetry transformations used to generate equivalent atoms:

Table S4. Anisotropic displacement parameters ($A^2 \times 10^3$) for **2i**. The anisotropic displacement factor exponent takes the form: −2 pi^2 [h^2 a^2 U11 + ... + 2 h k a × b × U12].

U11	U22	U33	U23	U13	U12	
C (1)	97 (3)	85 (3)	66 (2)	−1 (2)	7 (2)	17 (2)
C (2)	100 (3)	78 (2)	71 (3)	−9 (2)	−14 (2)	11 (2)
C (3)	75 (2)	73 (2)	79 (3)	−1 (2)	−6 (2)	−6 (2)
C (4)	69 (2)	60 (2)	65 (2)	−1 (2)	−4 (2)	−2 (2)
C (5)	70 (2)	61 (2)	70 (2)	0 (2)	6 (2)	1 (2)
C (6)	79 (2)	71 (2)	85 (3)	3 (2)	9 (2)	6 (2)
C (7)	68 (2)	89 (3)	74 (2)	4 (2)	1 (2)	−20 (2)
C (8)	77 (2)	86 (3)	76 (2)	1 (2)	−2 (2)	−1 (2)
C (9)	67 (2)	80 (2)	77 (2)	−9 (2)	−1 (2)	−15 (2)
C (10)	58 (2)	83 (3)	86 (3)	−7 (2)	3 (2)	−12 (2)
C (11)	94 (3)	78 (2)	86 (2)	25 (2)	5 (2)	17 (2)
C (12)	90 (3)	90 (3)	73 (2)	18 (2)	0 (2)	13 (2)
C (13)	111 (3)	73 (2)	75 (2)	0 (2)	−4 (2)	15 (2)
C (14)	105 (3)	78 (3)	85 (3)	−9 (2)	−2 (2)	13 (2)
C (15)	55 (2)	65 (2)	64 (2)	5 (2)	−6 (2)	−5 (2)
C (16)	69 (2)	70 (2)	74 (2)	−6 (2)	−8 (2)	−2 (2)
C (17)	68 (2)	101 (3)	89 (3)	−21 (3)	−4 (2)	−7 (2)
C (18)	84 (3)	127 (4)	67 (3)	−5 (3)	2 (2)	−1 (3)
C (19)	84 (3)	122 (4)	87 (3)	39 (3)	−13 (2)	−7 (3)
C (20)	79 (2)	72 (2)	80 (3)	12 (2)	−6 (2)	−1 (2)
F (1)	153 (2)	229 (4)	69 (2)	0 (2)	3 (2)	−5 (3)
N (1)	78 (2)	77 (2)	75 (2)	6 (2)	2 (2)	−9 (2)
N (2)	64 (2)	56 (2)	67 (2)	3 (1)	−2 (1)	4 (1)
O (1)	79 (1)	79 (2)	81 (2)	10 (1)	−2 (1)	−25 (1)

Table S5. Hydrogen coordinates (×10^4) and isotropic displacement parameters ($A^2 \times 10^3$) for **2i**.

x	y	z	U (eq)	
H (1)	−1095	5988	5863	99
H (2)	959	7063	5601	99
H (3)	1690	7303	4367	91
H (6)	−2488	5185	4898	94
H (7)	893	5583	2967	92
H (8)	−1086	7498	2633	95
H (9)	−2300	5206	2308	90
H (10)	−2842	4741	3505	91
H (11A)	−485	4818	1399	103
H (11B)	990	5008	1770	103
H (12A)	1791	6140	708	101
H (12B)	1214	4744	459	101
H (13A)	−1229	7907	153	104
H (13B)	240	8138	525	104
H (14A)	−1470	8114	1449	107
H (14B)	−1948	6688	1192	107
H (16)	1077	4387	−661	85
H (17)	1486	4275	−1937	103
H (19)	−11	7946	−2189	117
H (20)	−333	8120	−894	92
H (1A)	1548	7695	2533	119

2. Copies of ^{1}H- and ^{13}C-NMR Spectra of Compounds 1a–1b, 2a–2x and 3a–3i

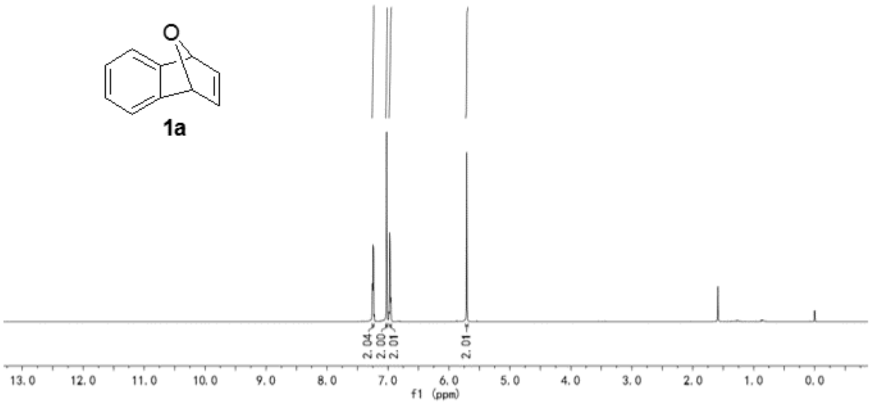

Figure S2. ^{1}H-NMR Spectra of Compound **1a**.

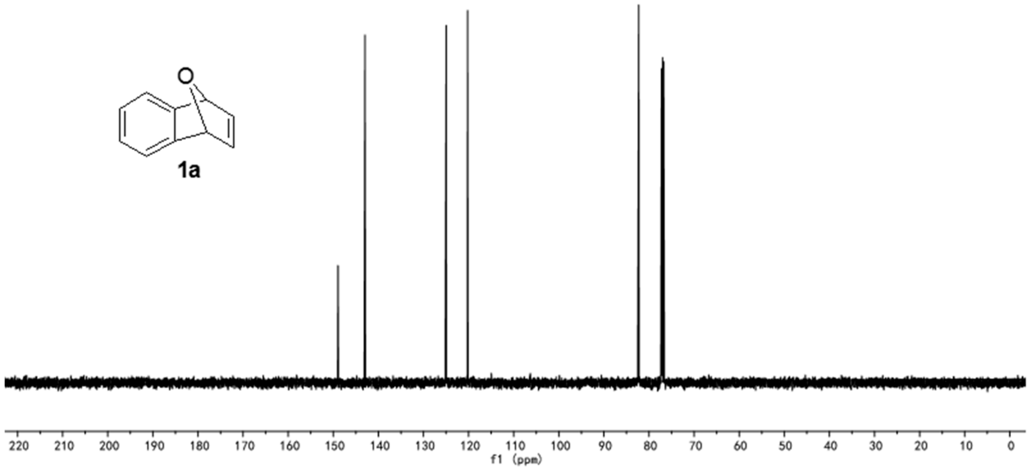

Figure S3. ^{13}C-NMR Spectra of Compound **1a**.

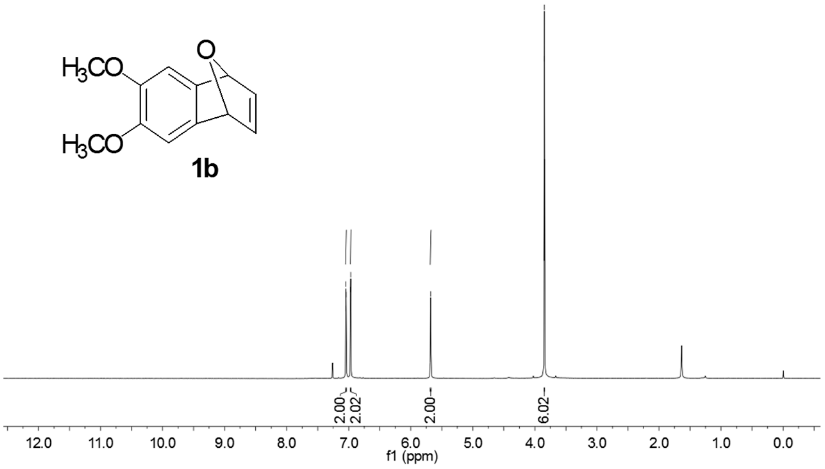

Figure S4. ^{1}H-NMR Spectra of Compound **1b**.

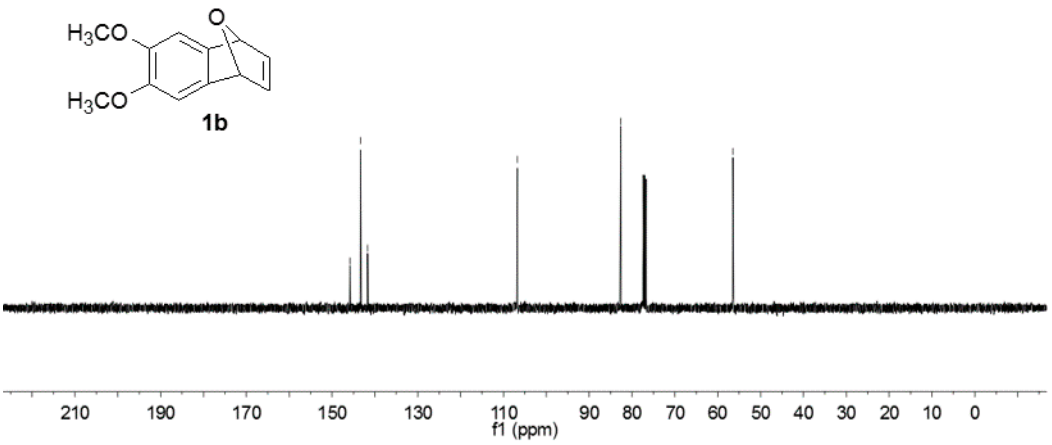

Figure S5. ^{13}C-NMR Spectra of Compound **1b**.

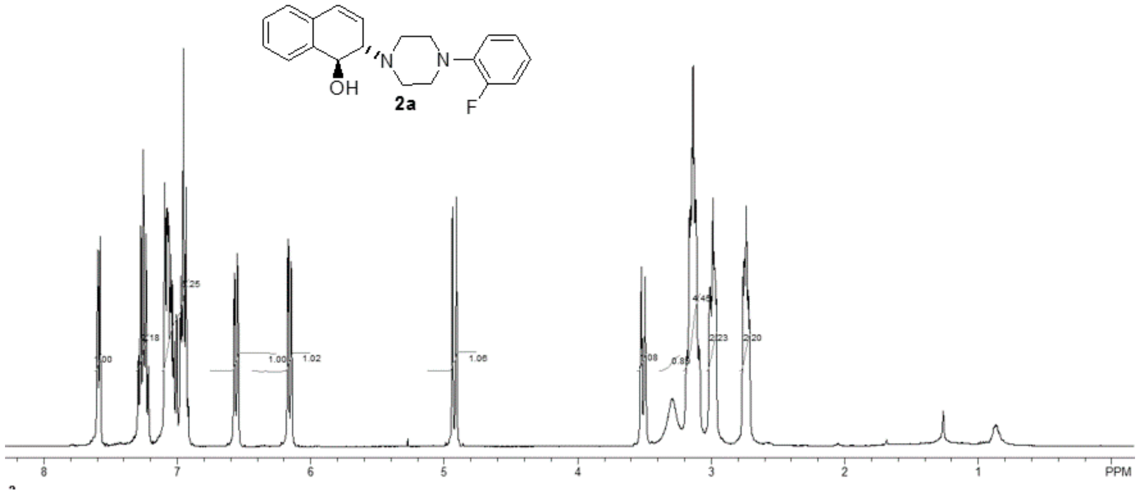

Figure S6. ^{1}H-NMR Spectra of Compound **2a**.

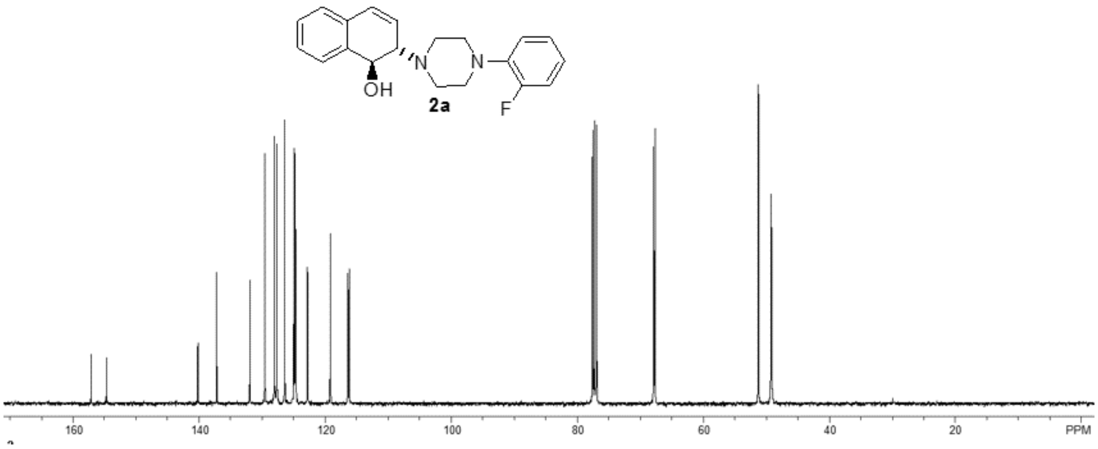

Figure S7. ^{13}C-NMR Spectra of Compound **2a**.

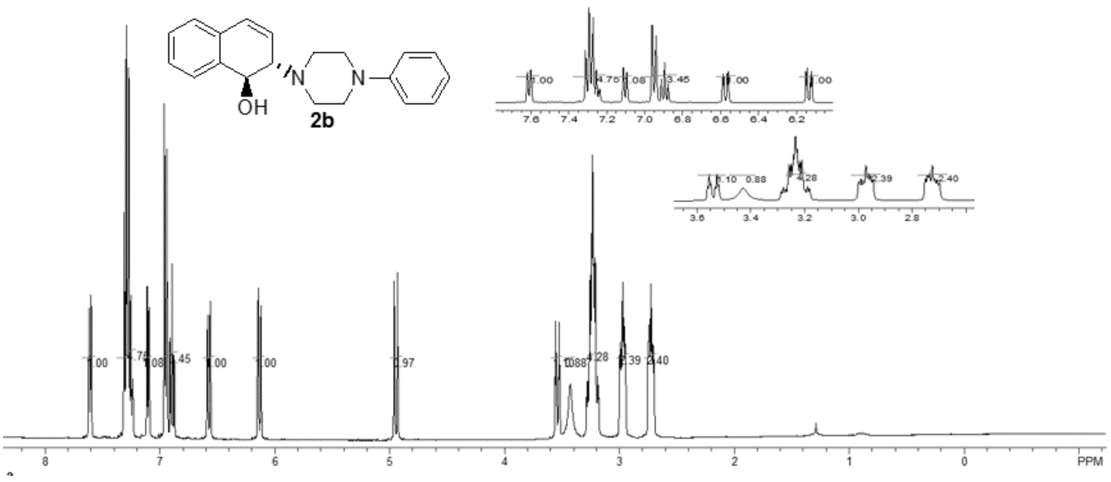

Figure S8. ^{1}H-NMR Spectra of Compound **2b**.

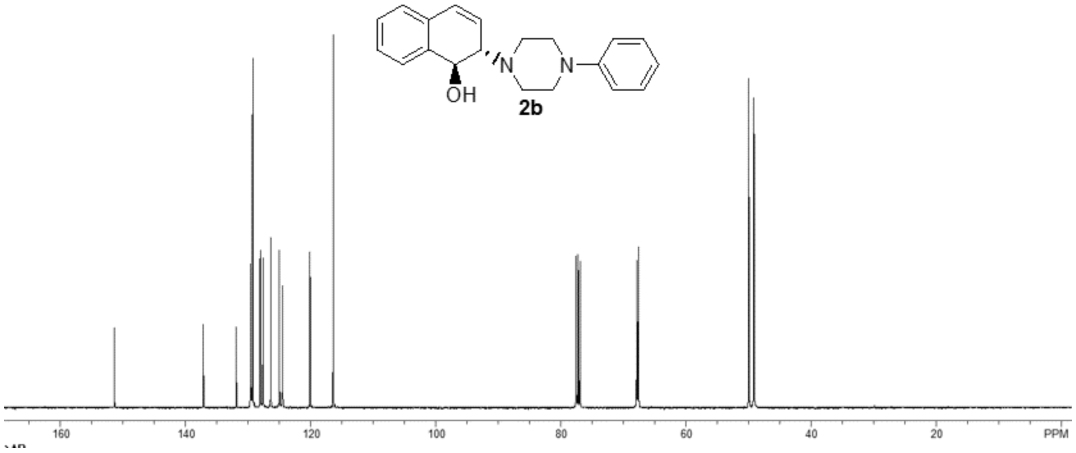

Figure S9. ^{13}C-NMR Spectra of Compound **2b**.

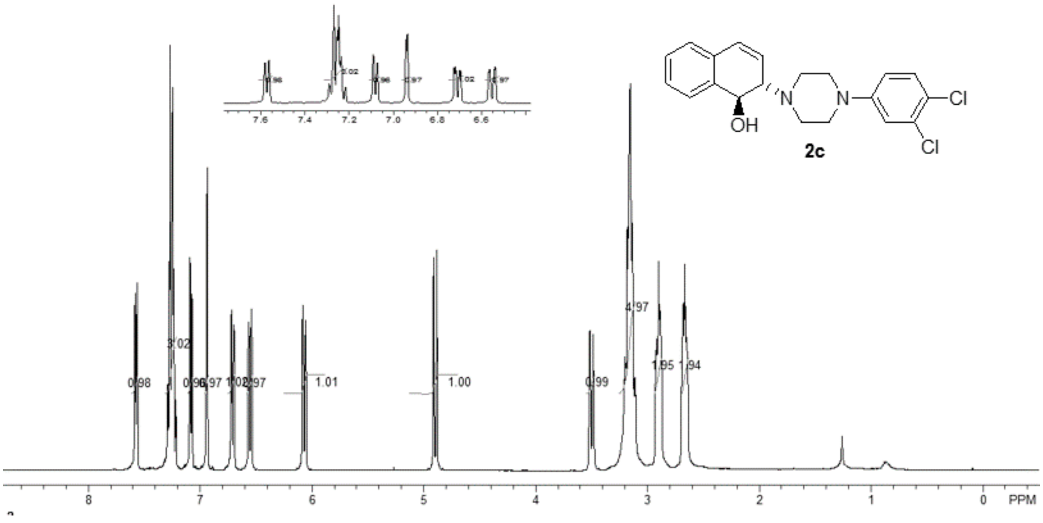

Figure S10. ^{1}H-NMR Spectra of Compound **2c**.

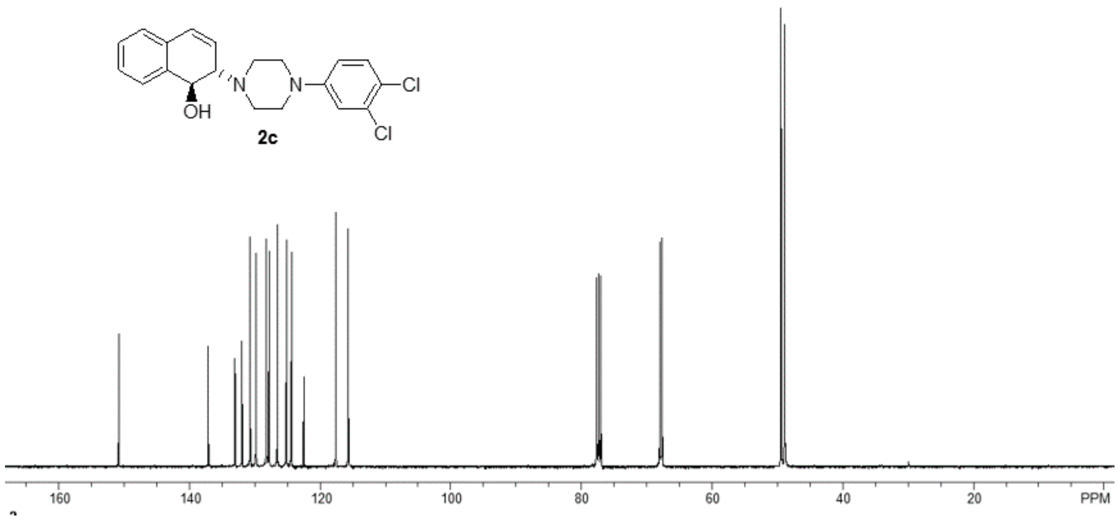

Figure S11. ^{13}C-NMR Spectra of Compound **2c**.

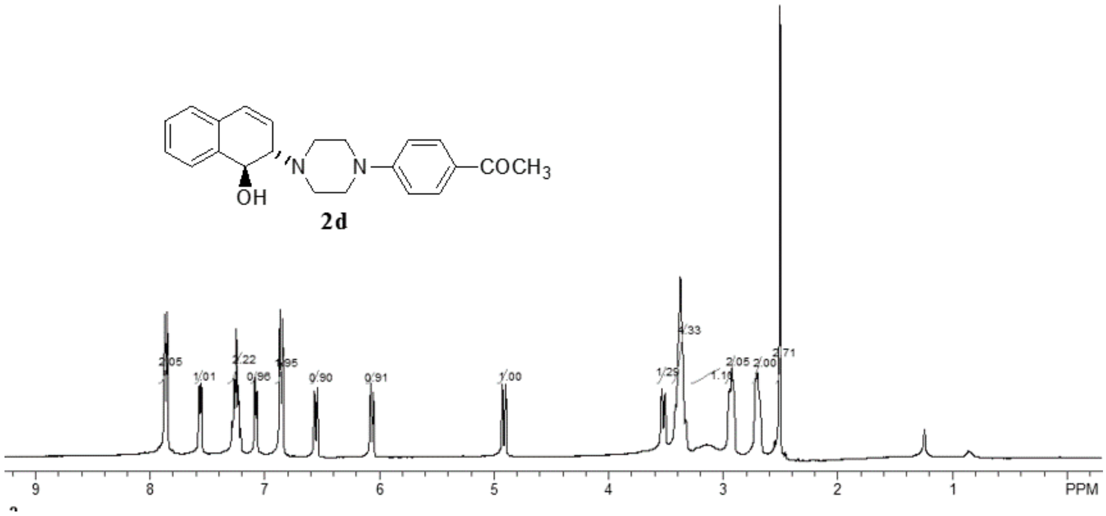

Figure S12. ^{1}H-NMR Spectra of Compound **2d**.

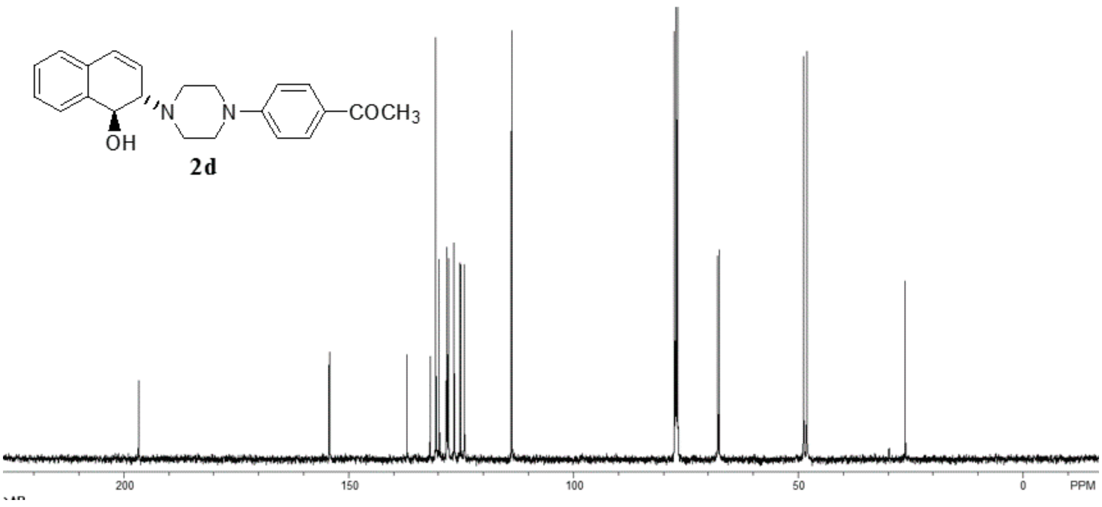

Figure S13. ^{13}C-NMR Spectra of Compound **2d**.

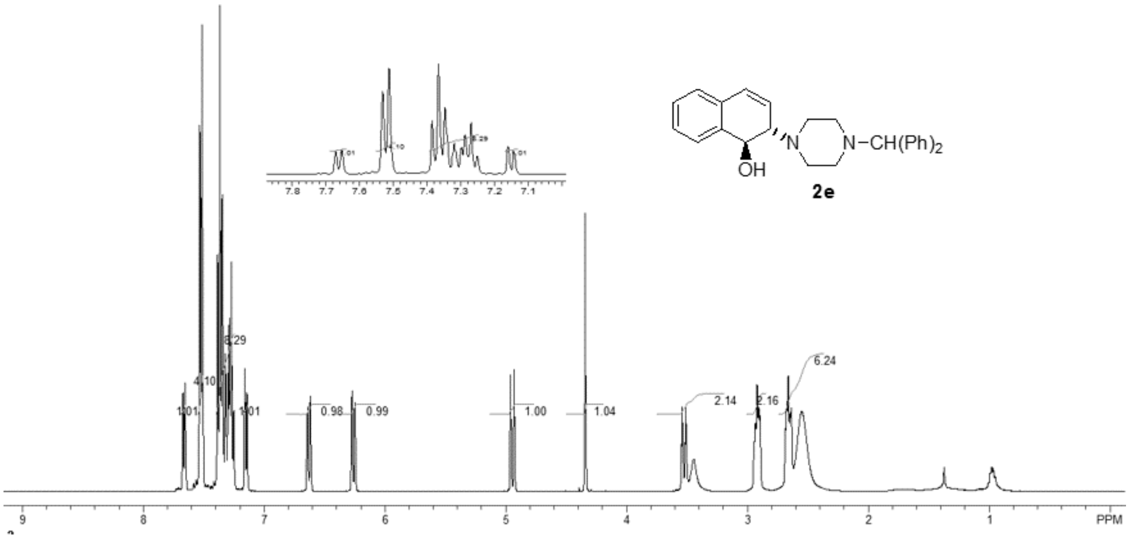

Figure S14. ^{1}H-NMR Spectra of Compound **2e**.

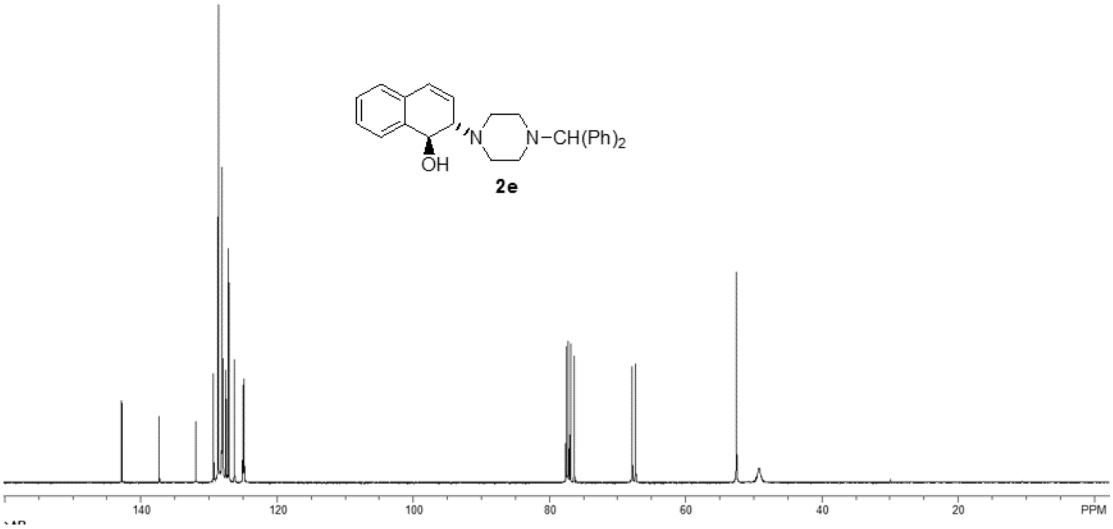

Figure S15. ^{13}C-NMR Spectra of Compound **2e**.

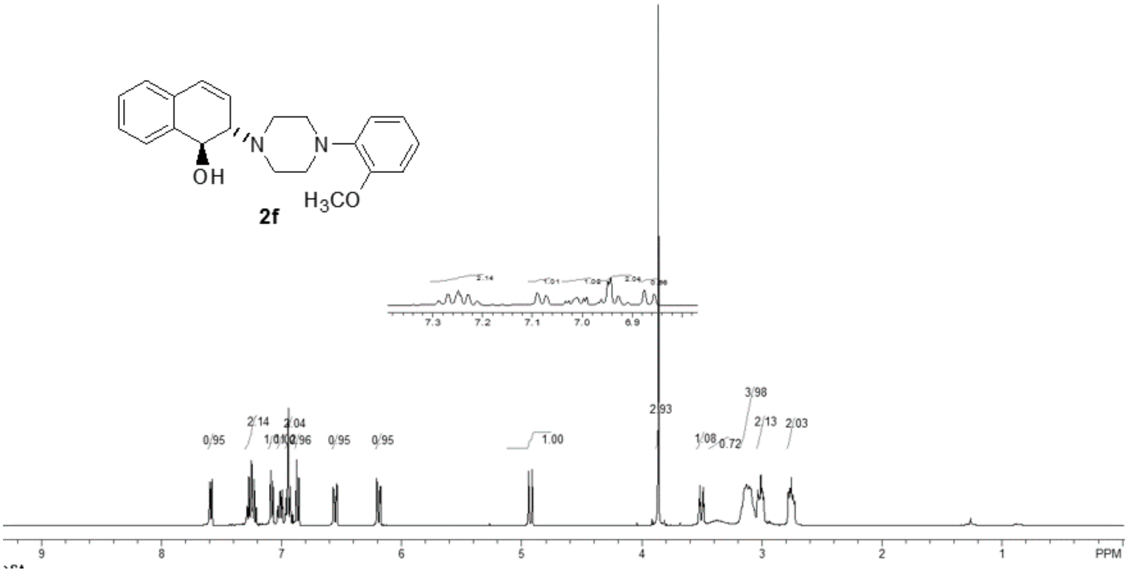

Figure S16. ^{1}H-NMR Spectra of Compound **2f**.

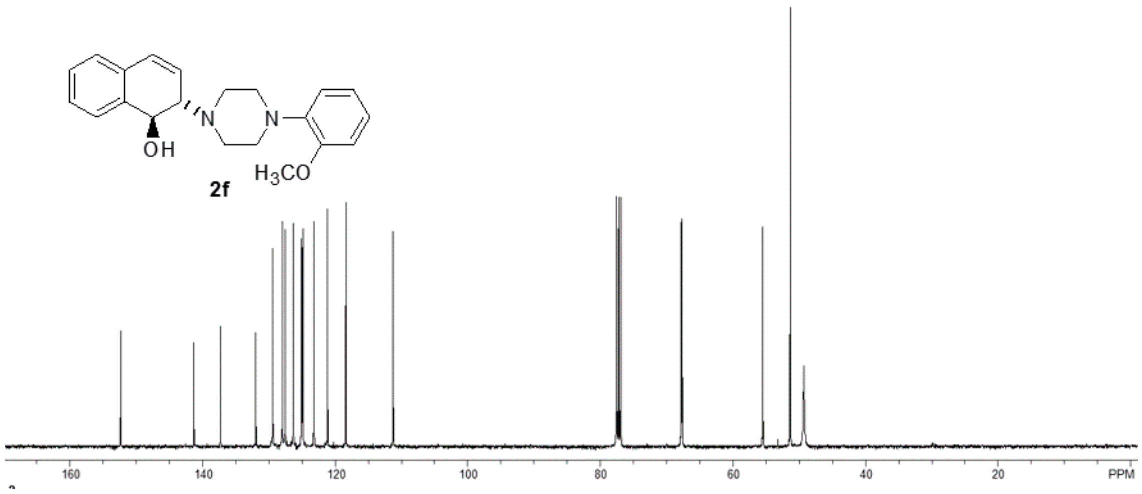

Figure S17. ^{13}C-NMR Spectra of Compound **2f**.

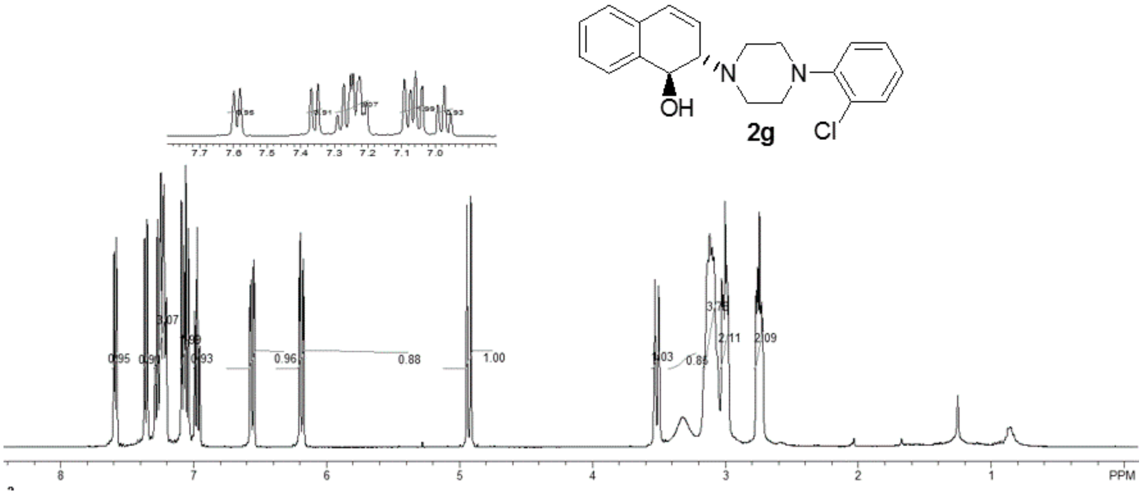

Figure S18. ^{1}H-NMR Spectra of Compound **2g**.

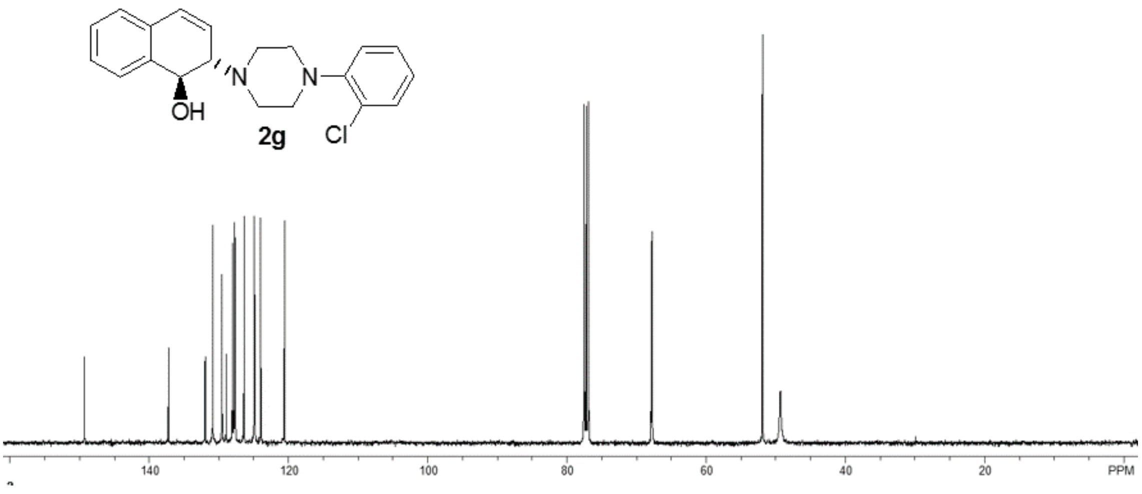

Figure S19. ^{13}C-NMR Spectra of Compound **2g**.

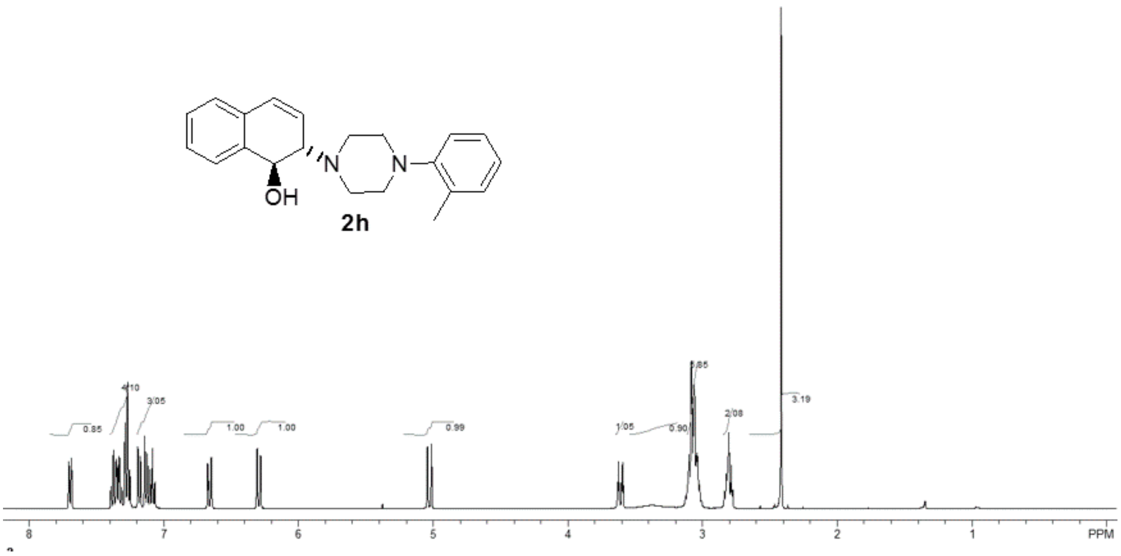

Figure S20. ^{1}H-NMR Spectra of Compound **2h**.

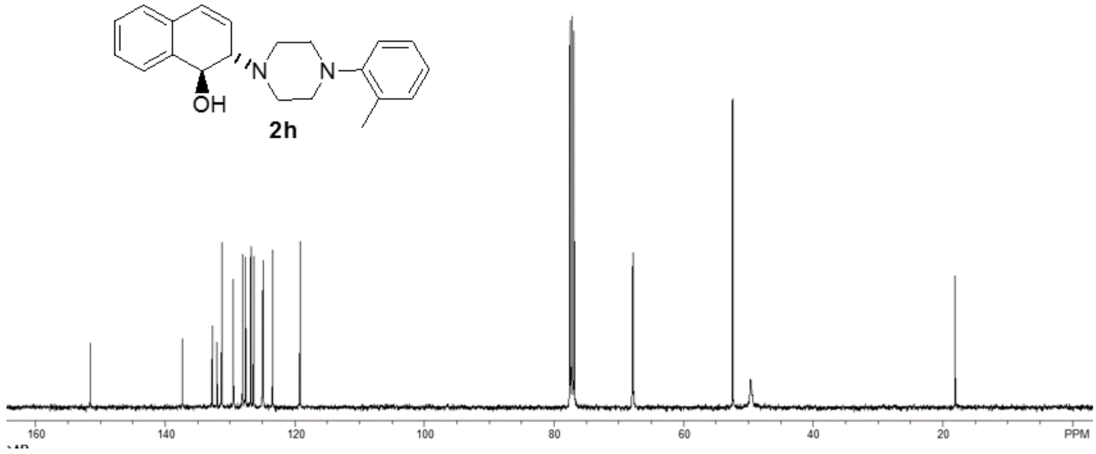

Figure S21. ^{13}C-NMR Spectra of Compound **2h**.

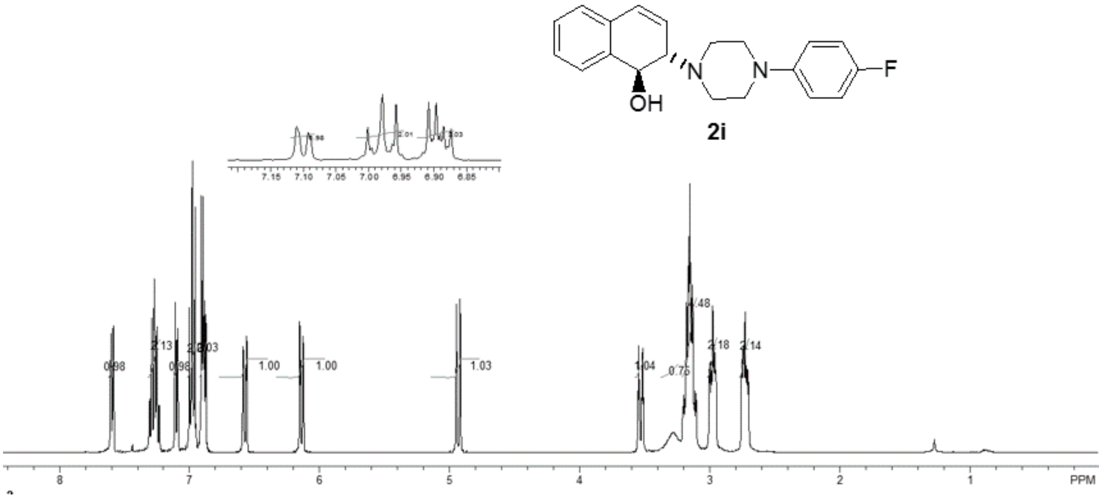

Figure S22. ^{1}H-NMR Spectra of Compound **2i**.

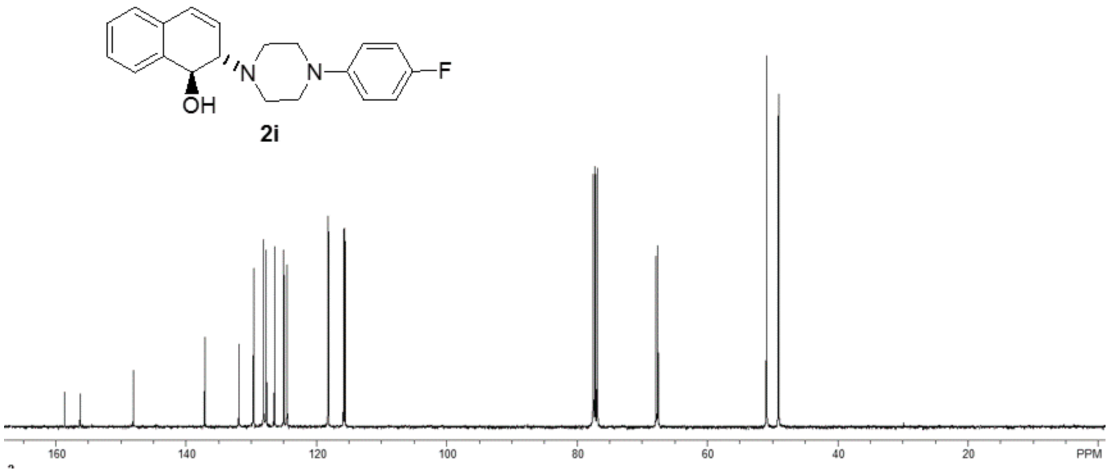

Figure S23. ^{13}C-NMR Spectra of Compound **2i**.

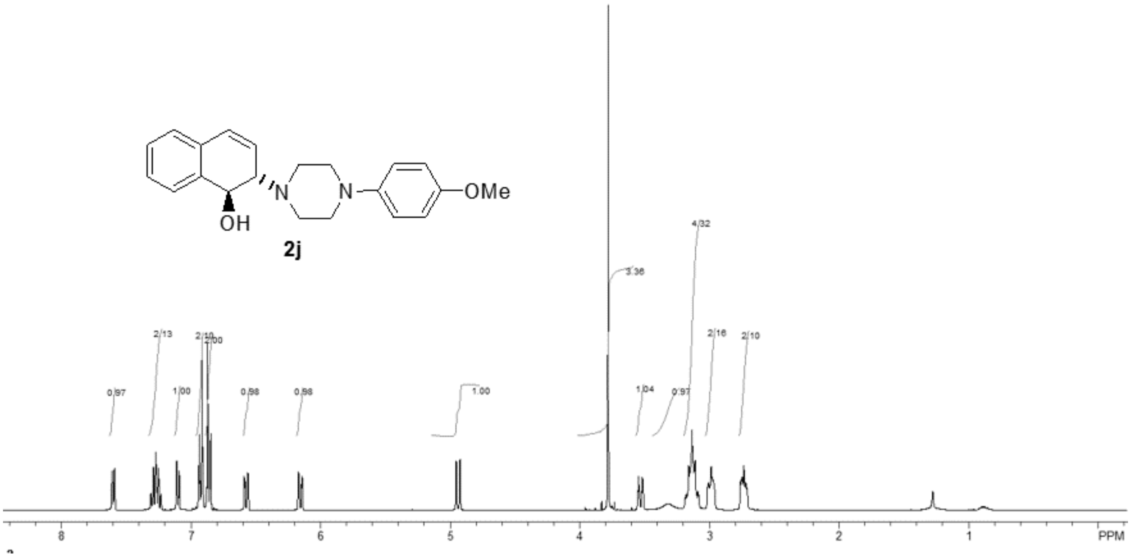

Figure S24. ^{1}H-NMR Spectra of Compound **2j**.

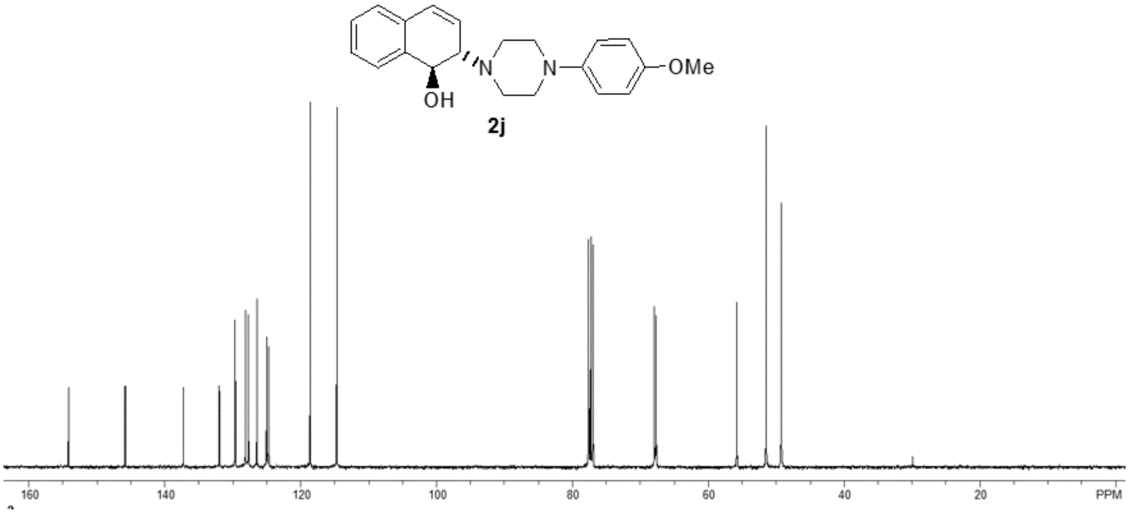

Figure S25. ^{13}C-NMR Spectra of Compound **2j**.

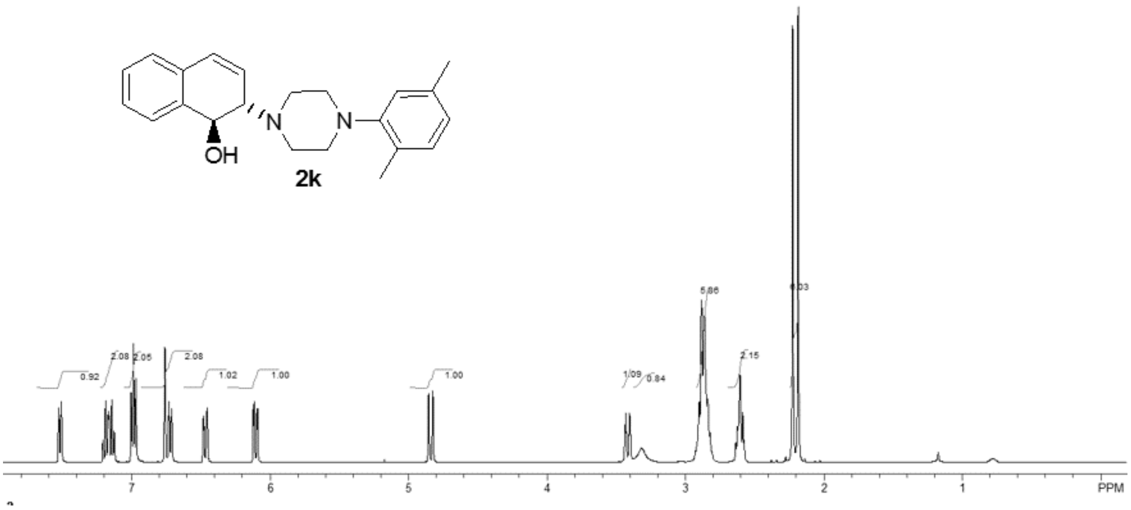

Figure S26. ^{1}H-NMR Spectra of Compound **2k**.

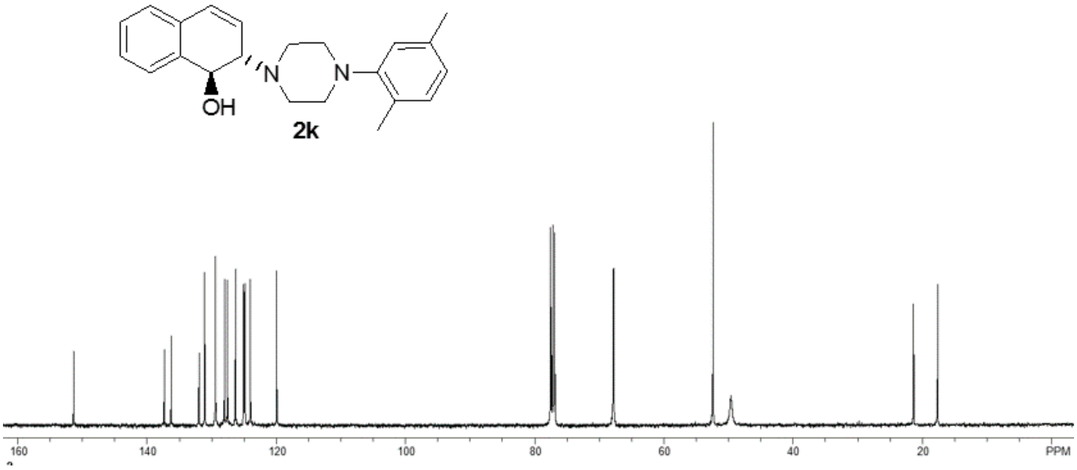

Figure S27. ^{13}C-NMR Spectra of Compound **2k**.

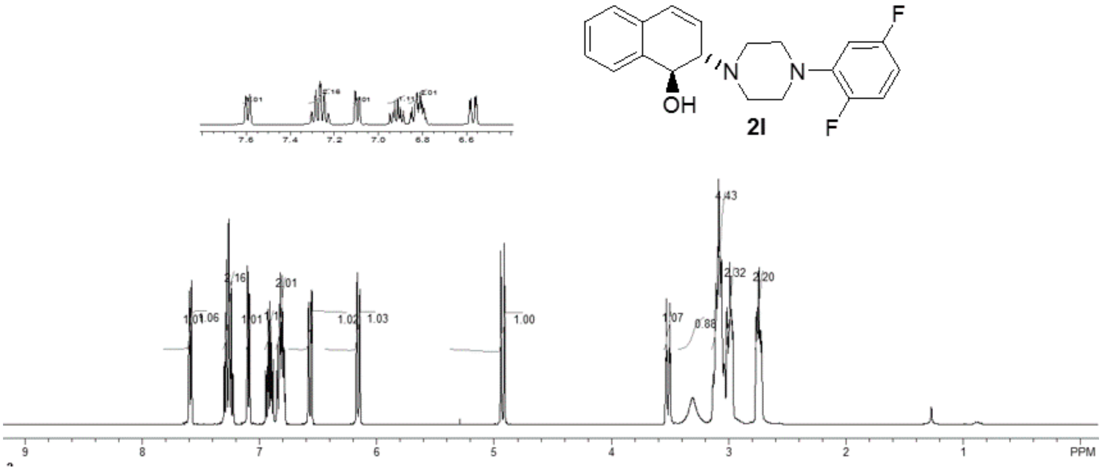

Figure S28. ^{1}H-NMR Spectra of Compound **2l**.

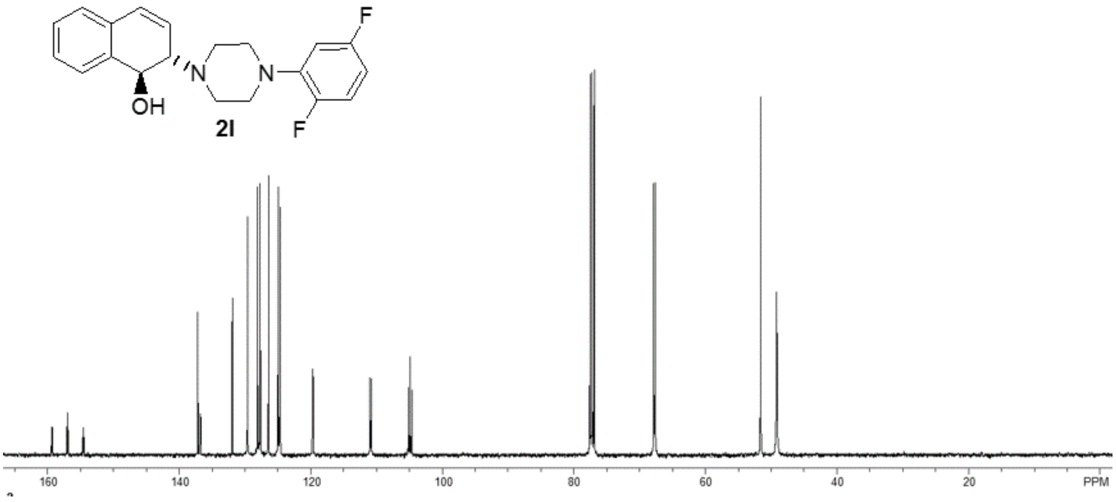

Figure S29. ^{13}C-NMR Spectra of Compound **2l**.

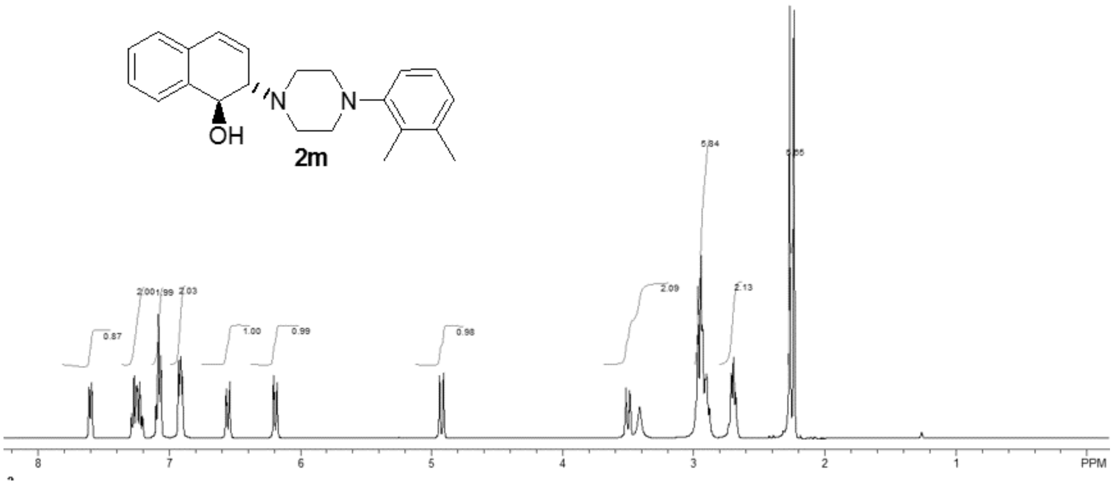

Figure S30. ^{1}H-NMR Spectra of Compound **2m**.

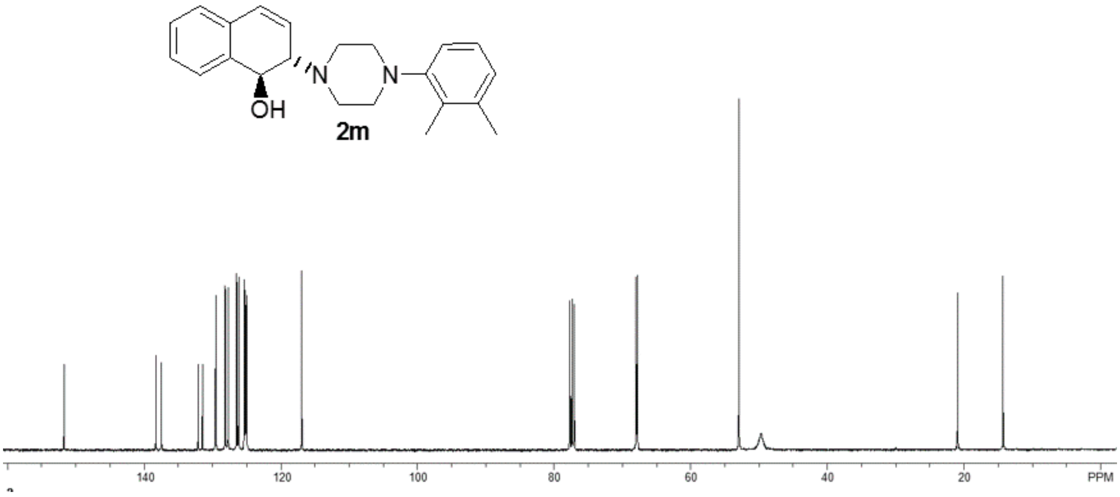

Figure S31. ^{13}C-NMR Spectra of Compound **2m**.

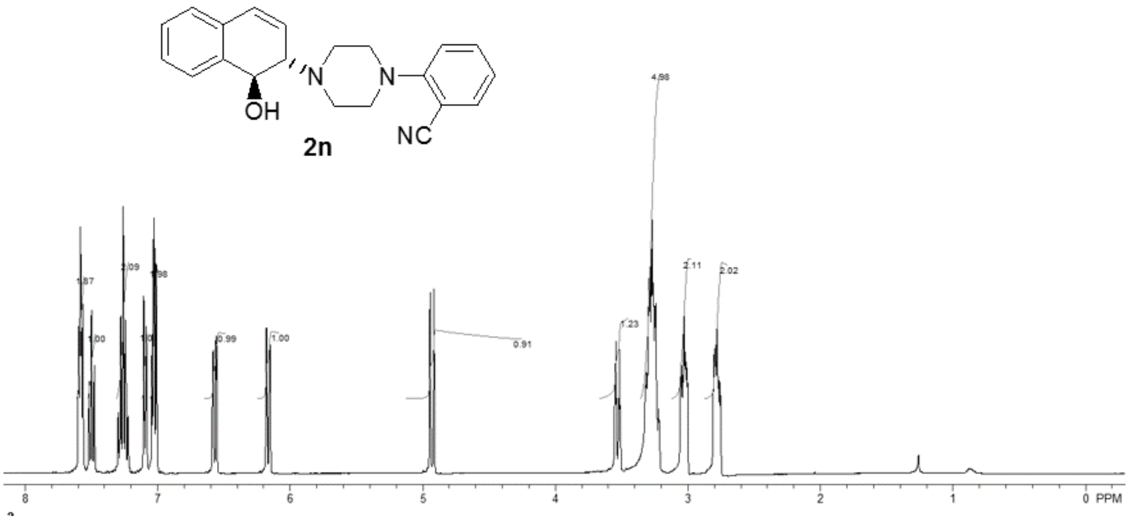

Figure S32. ^{1}H-NMR Spectra of Compound **2n**.

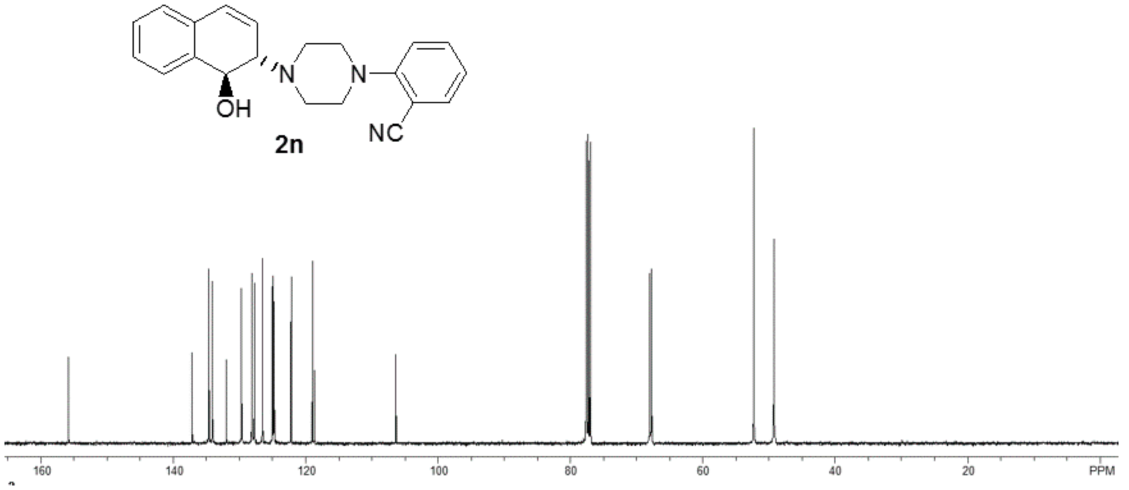

Figure S33. ^{13}C-NMR Spectra of Compound **2n**.

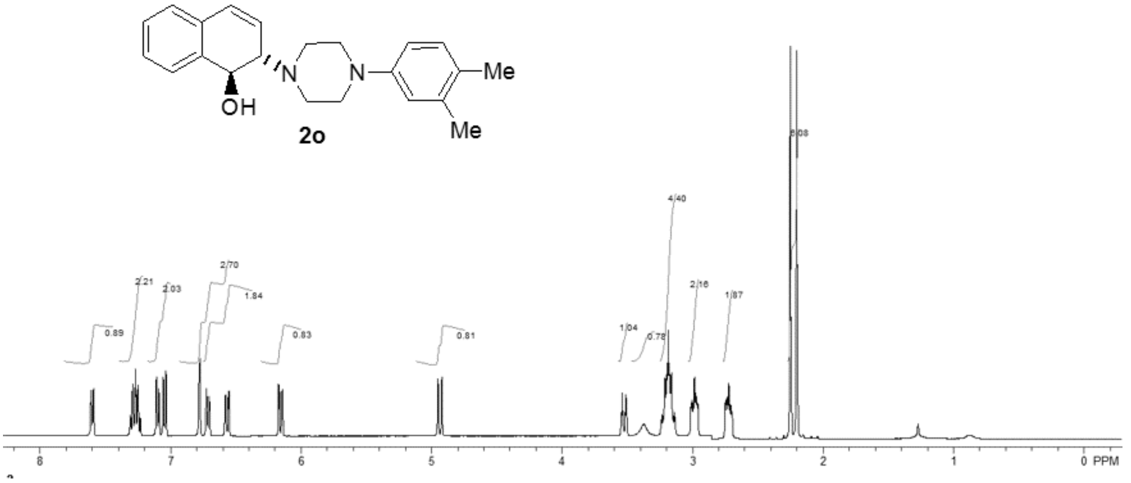

Figure S34. ^{1}H-NMR Spectra of Compound **2o**.

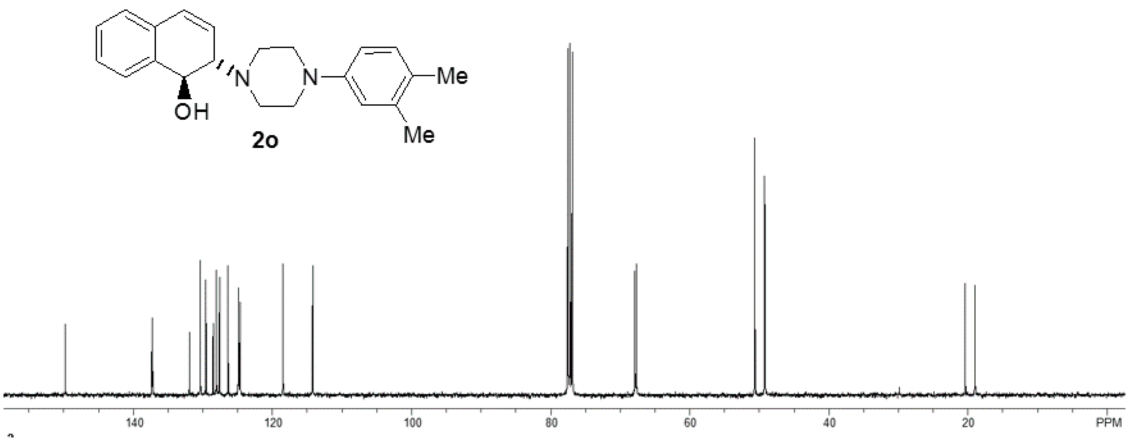

Figure S35. ^{13}C-NMR Spectra of Compound **2o**.

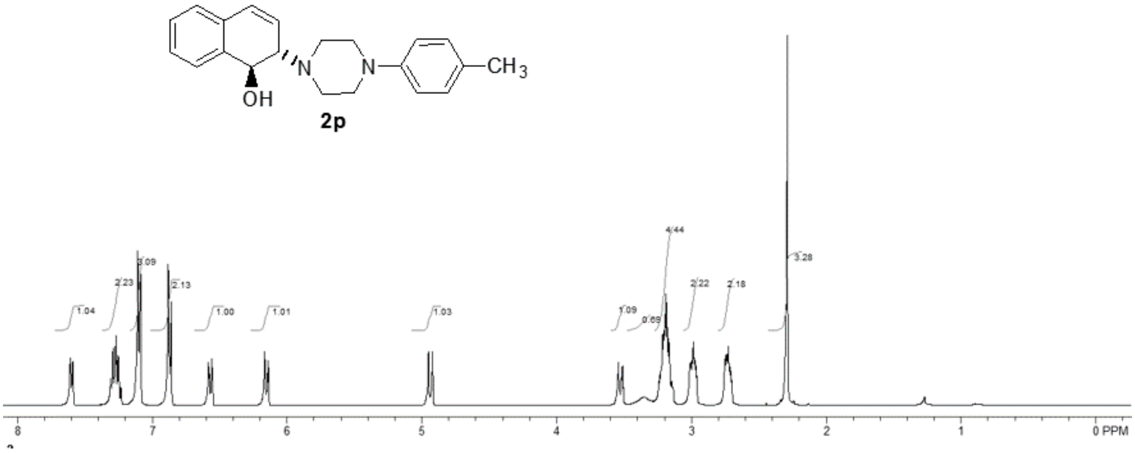

Figure S36. ^{1}H-NMR Spectra of Compound **2p**.

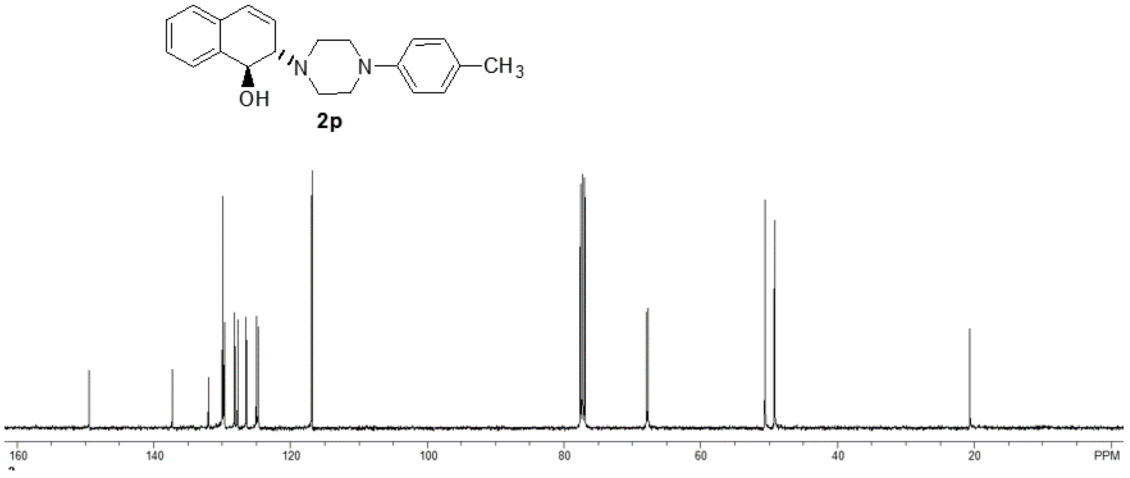

Figure S37. ^{13}C-NMR Spectra of Compound **2p**.

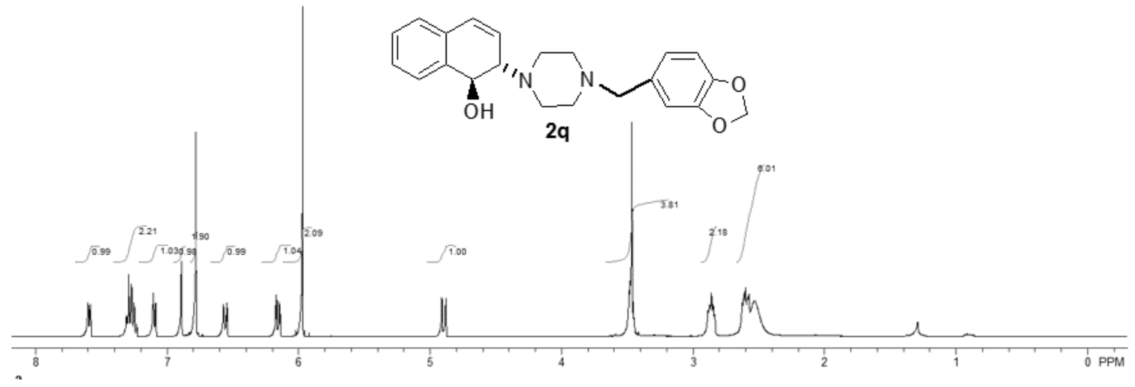

Figure S38. ^{1}H-NMR Spectra of Compound **2q**.

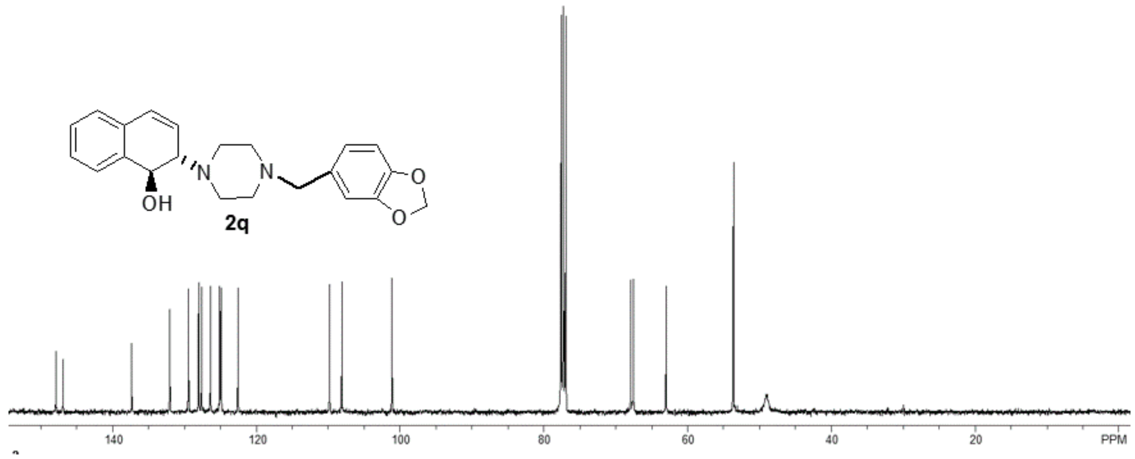

Figure S39. ^{13}C-NMR Spectra of Compound **2q**.

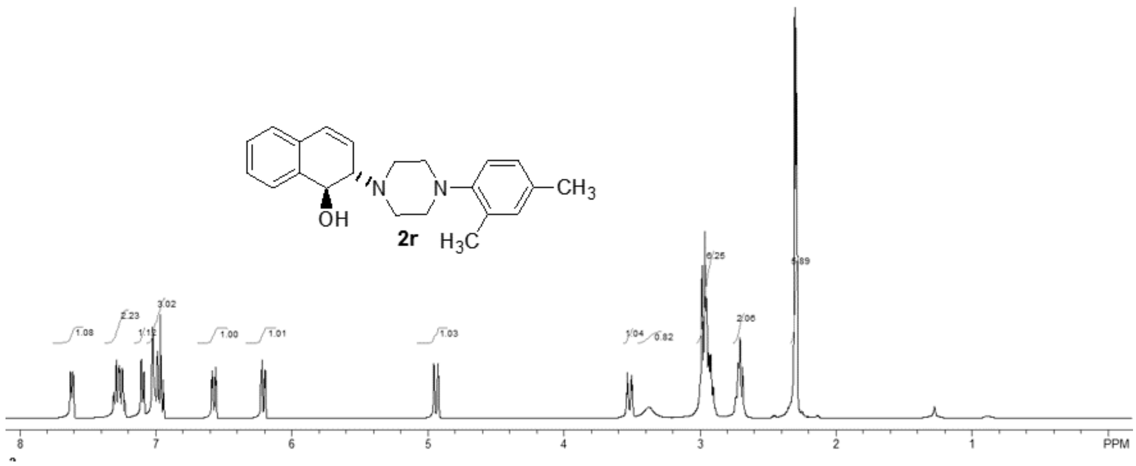

Figure S40. ^{1}H-NMR Spectra of Compound **2r**.

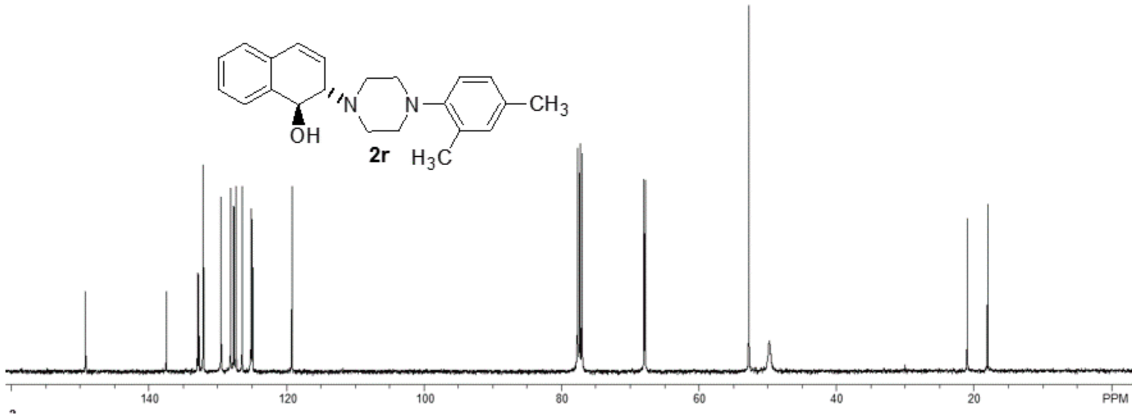

Figure S41. ^{13}C-NMR Spectra of Compound **2r**.

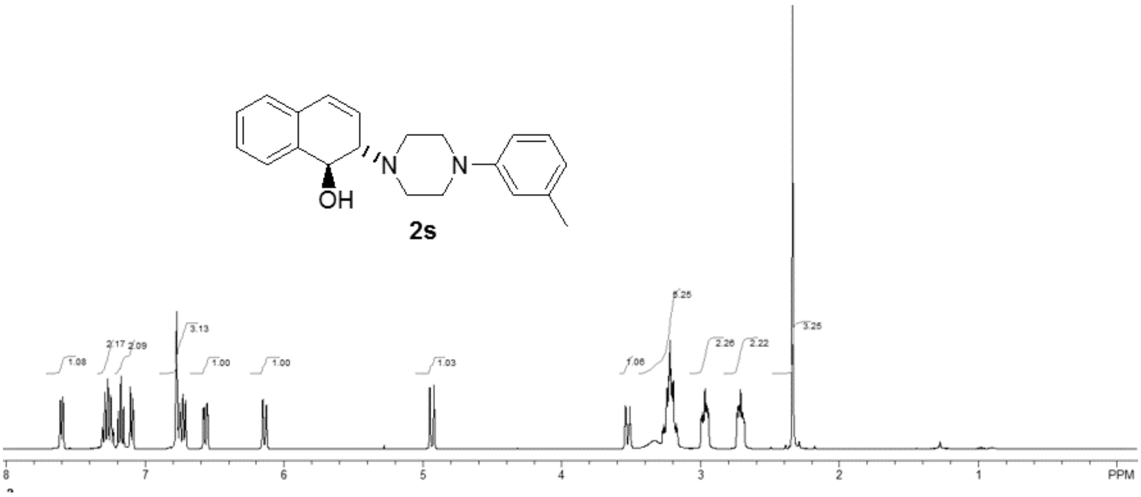

Figure S42. ^{1}H-NMR Spectra of Compound **2s**.

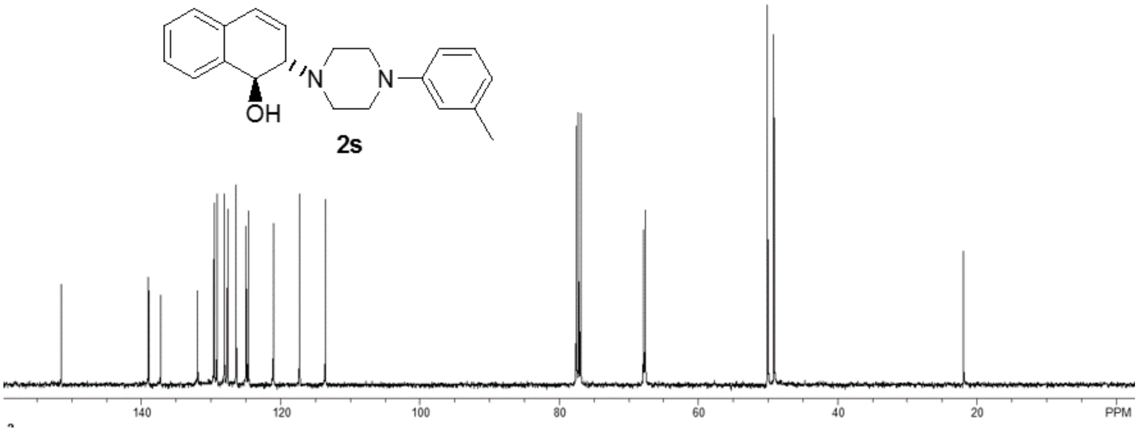

Figure S43. ^{13}C-NMR Spectra of Compound **2s**.

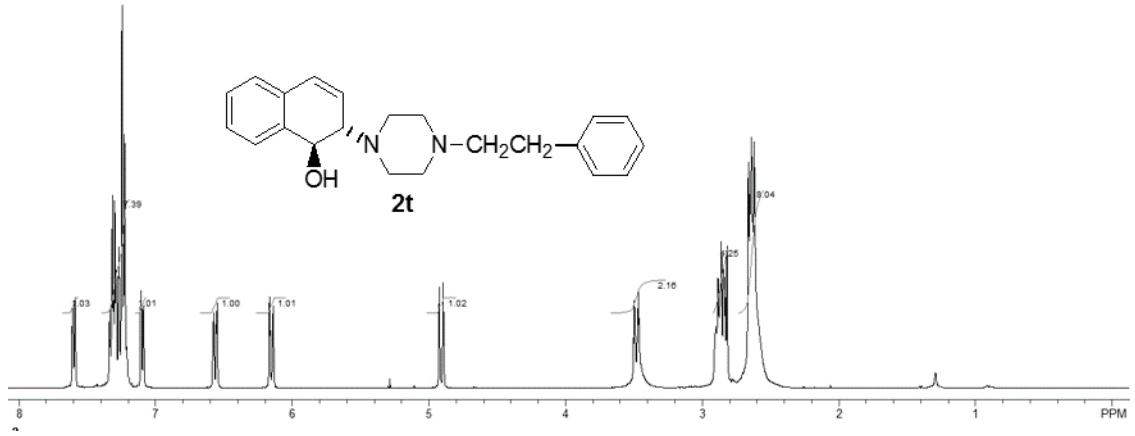

Figure S44. ^{1}H-NMR Spectra of Compound **2t**.

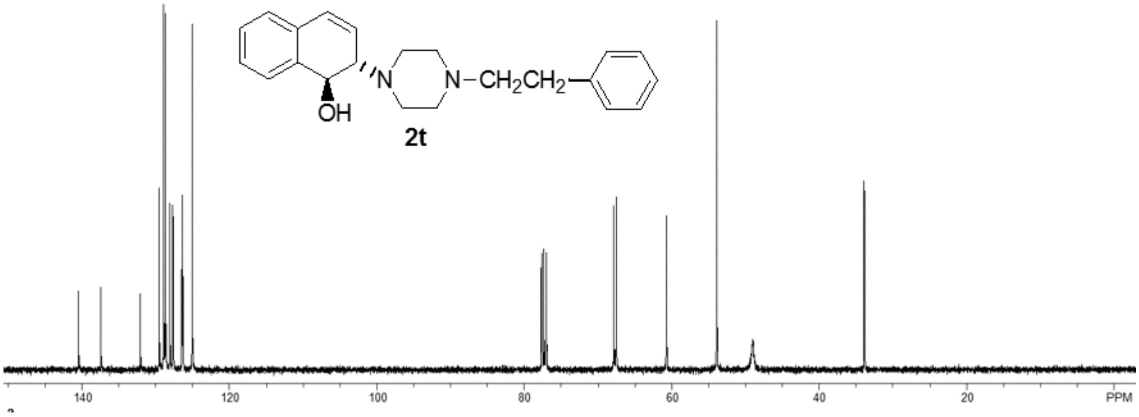

Figure S45. ^{13}C-NMR Spectra of Compound **2t**.

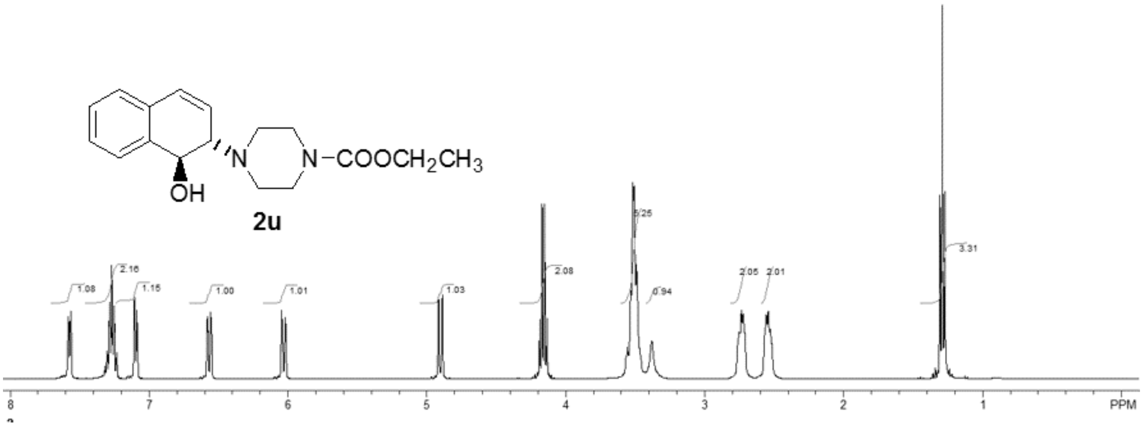

Figure S46. ^{1}H-NMR Spectra of Compound **2u**.

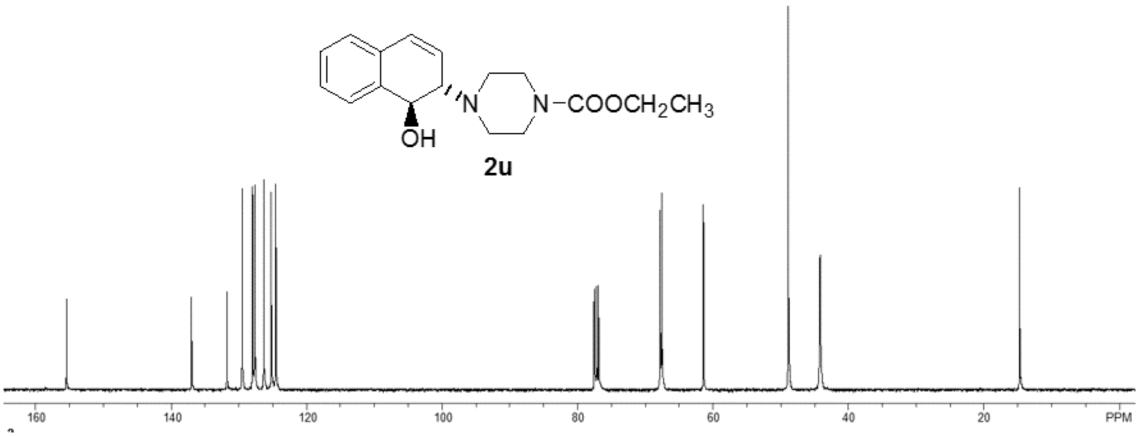

Figure S47. ^{13}C-NMR Spectra of Compound **2u**.

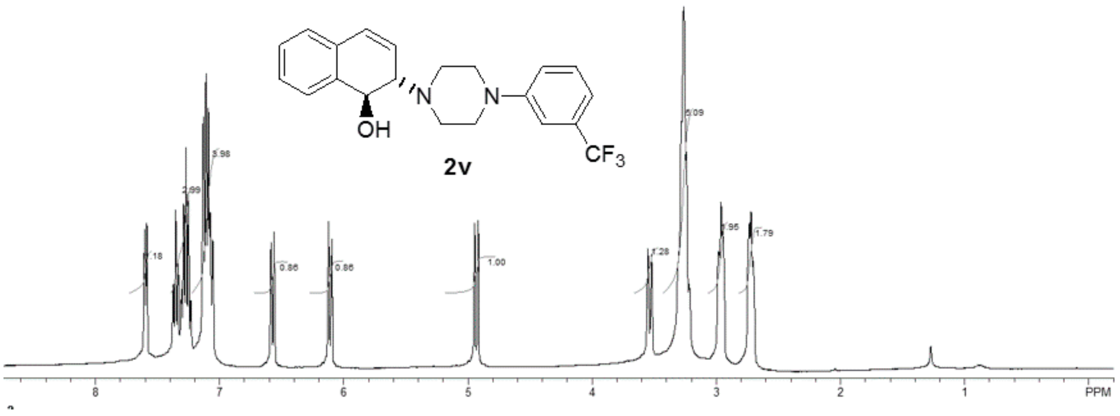

Figure S48. ^{1}H-NMR Spectra of Compound **2v**.

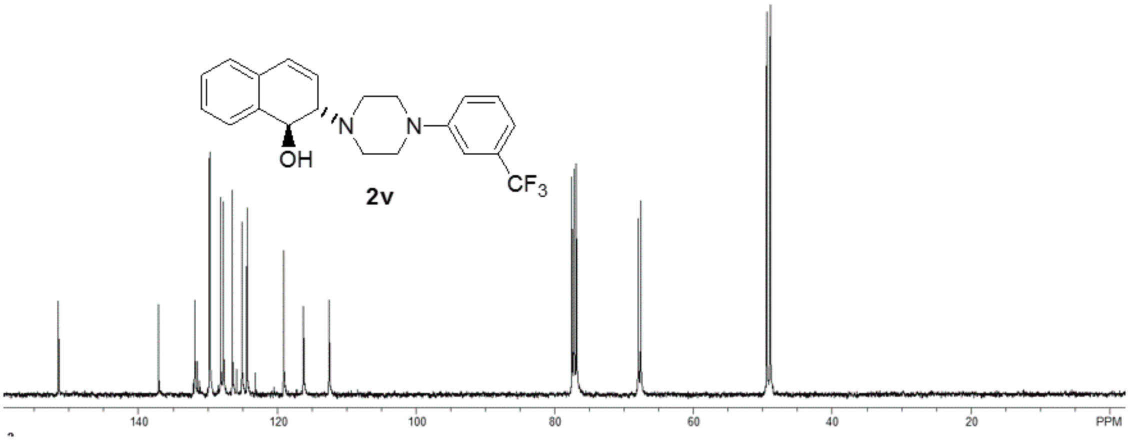

Figure S49. ^{13}C-NMR Spectra of Compound **2v**.

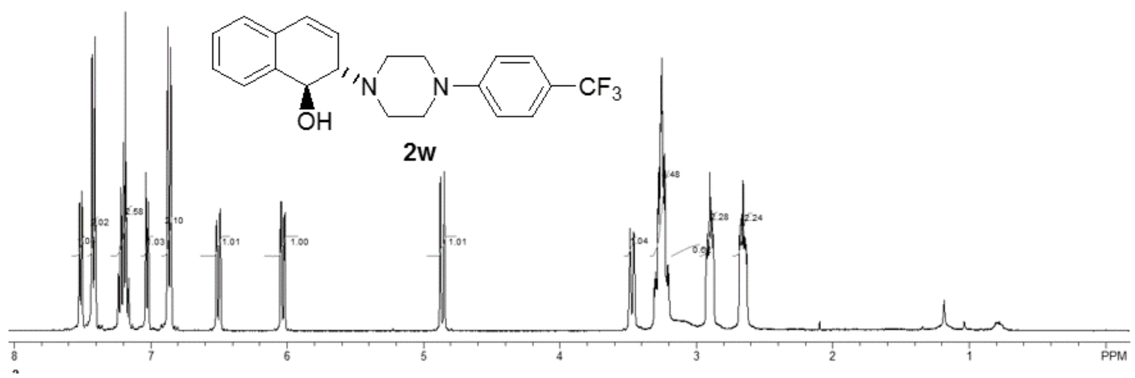

Figure S50. ^{1}H-NMR Spectra of Compound **2w**.

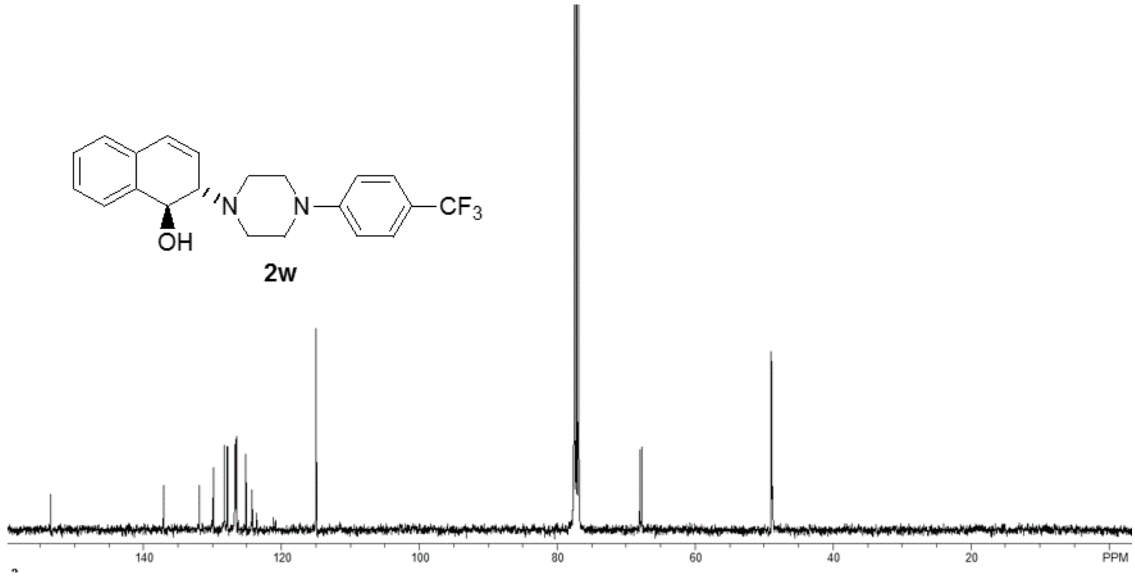

Figure S51. ^{13}C-NMR Spectra of Compound **2w**.

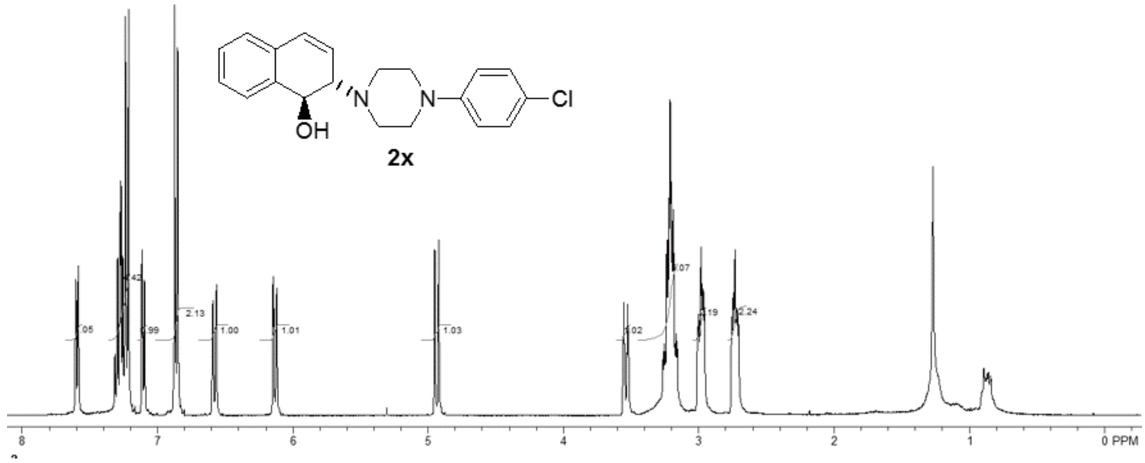

Figure S52. ^{1}H-NMR Spectra of Compound **2x**.

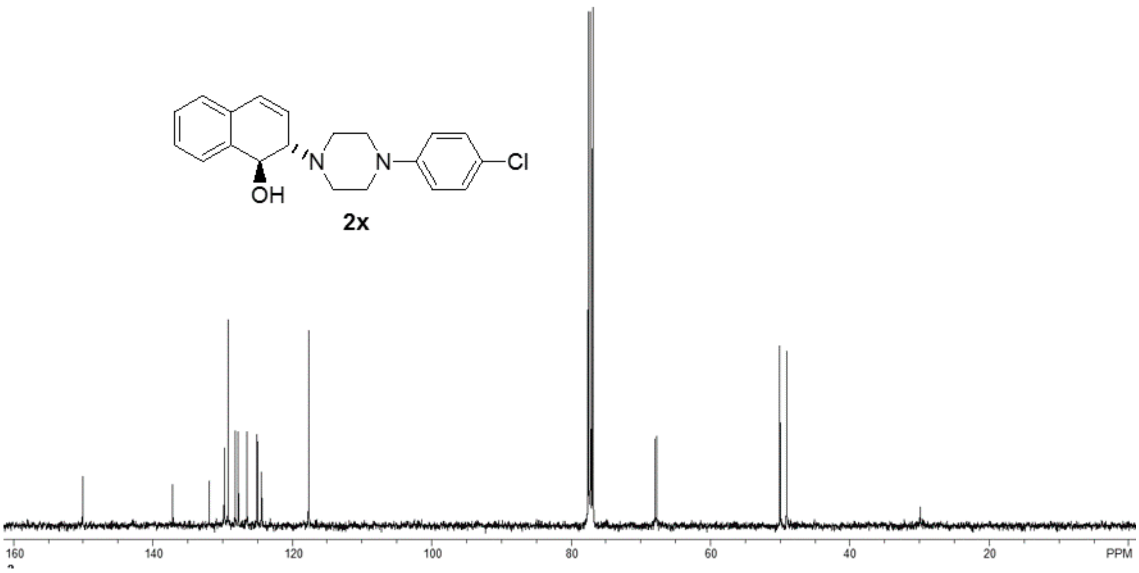

Figure S53. ^{13}C-NMR Spectra of Compound **2x**.

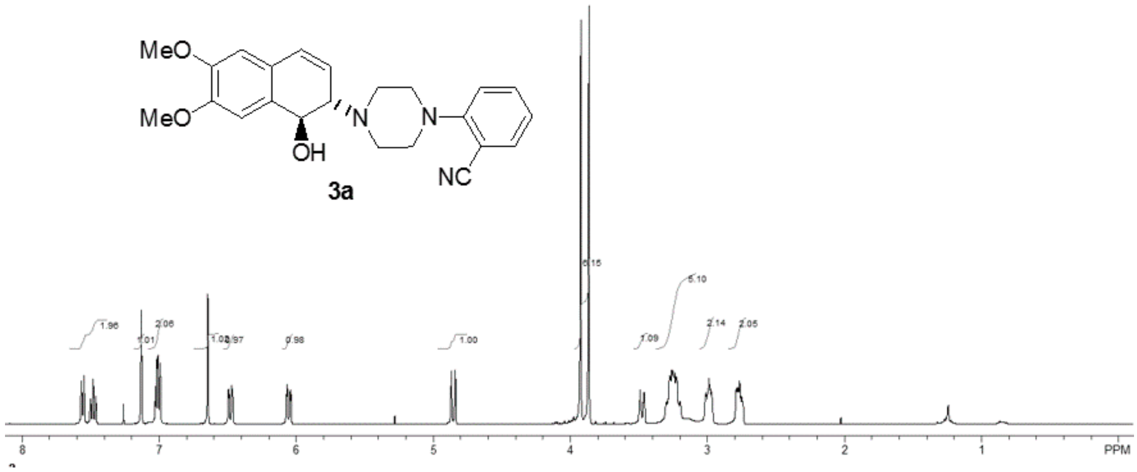

Figure S54. ^{1}H-NMR Spectra of Compound **3a**.

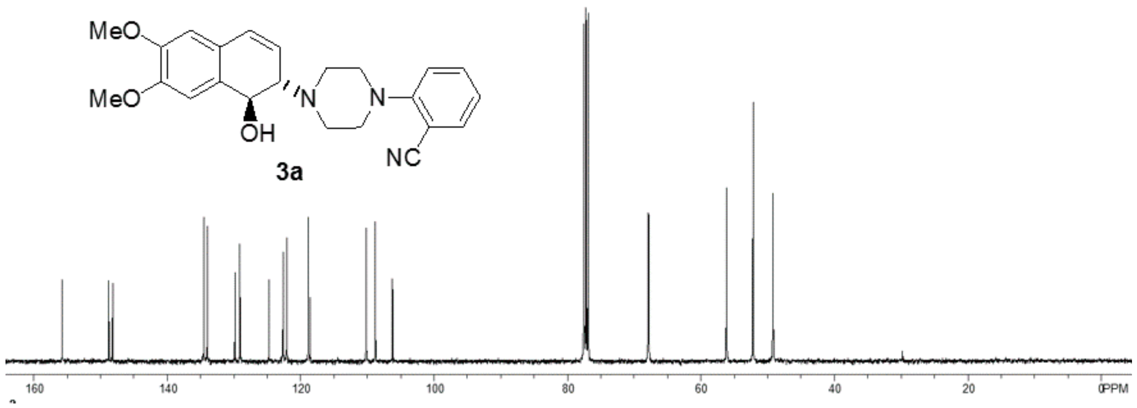

Figure S55. ^{13}C-NMR Spectra of Compound **3a**.

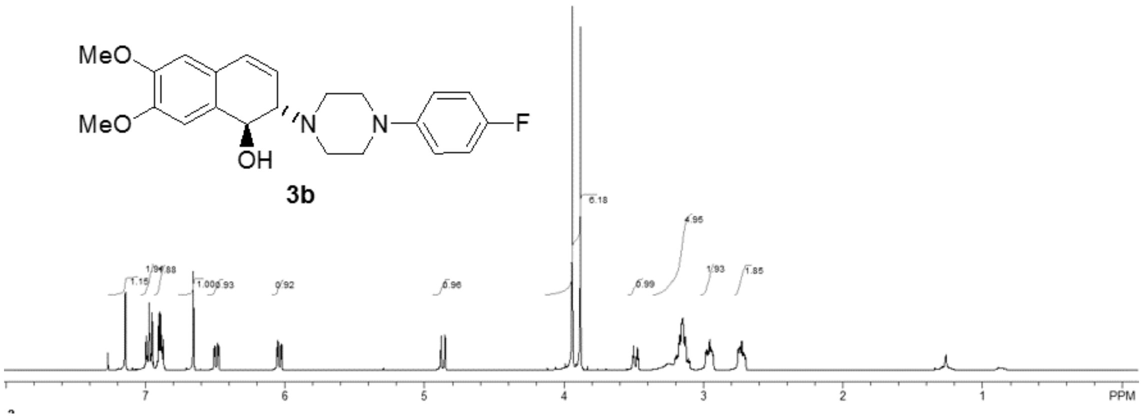

Figure S56. ^{1}H-NMR Spectra of Compound **3b**.

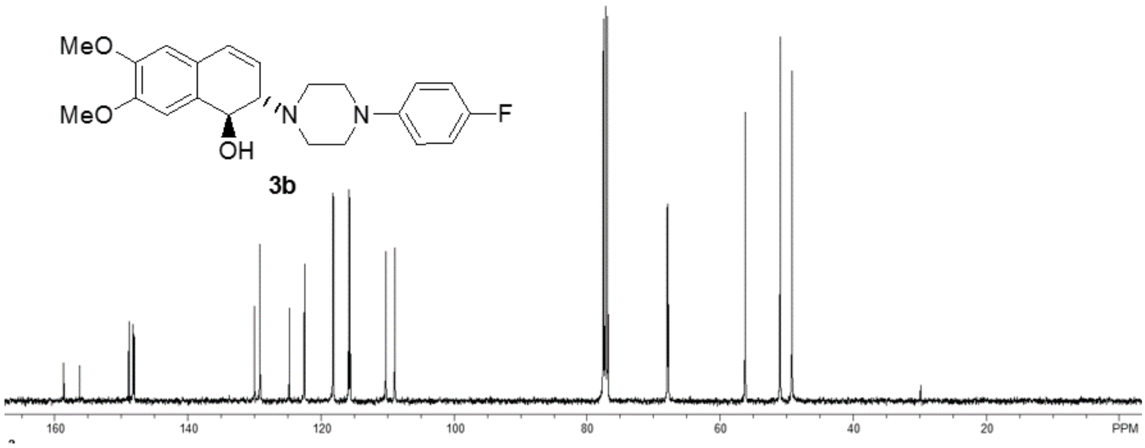

Figure S57. ^{13}C-NMR Spectra of Compound **3b**.

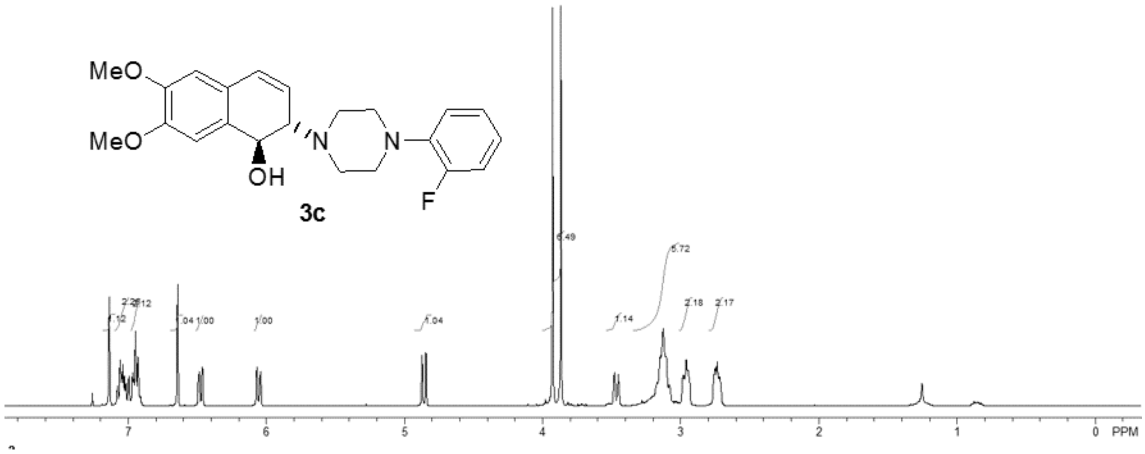

Figure S58. ^{1}H-NMR Spectra of Compound **3c**.

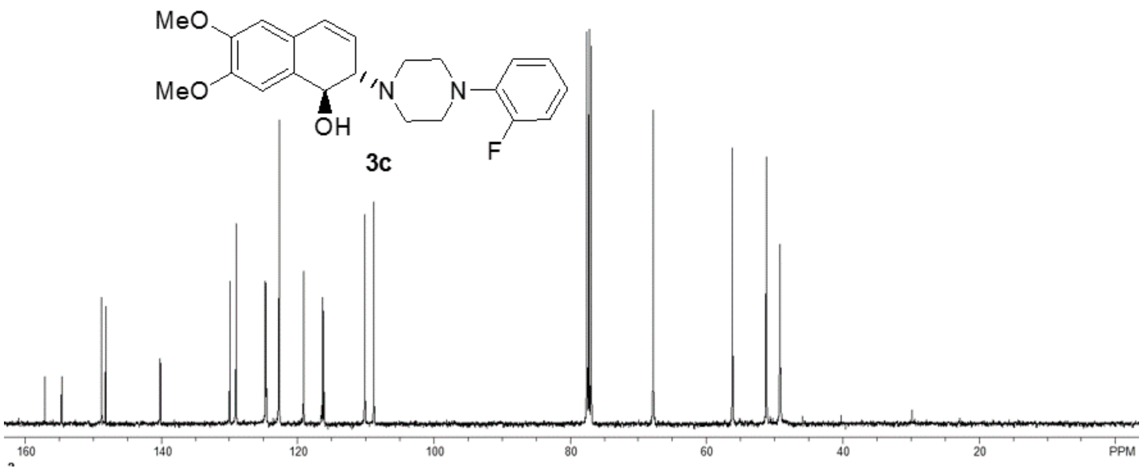

Figure S59. ^{13}C-NMR Spectra of Compound **3c**.

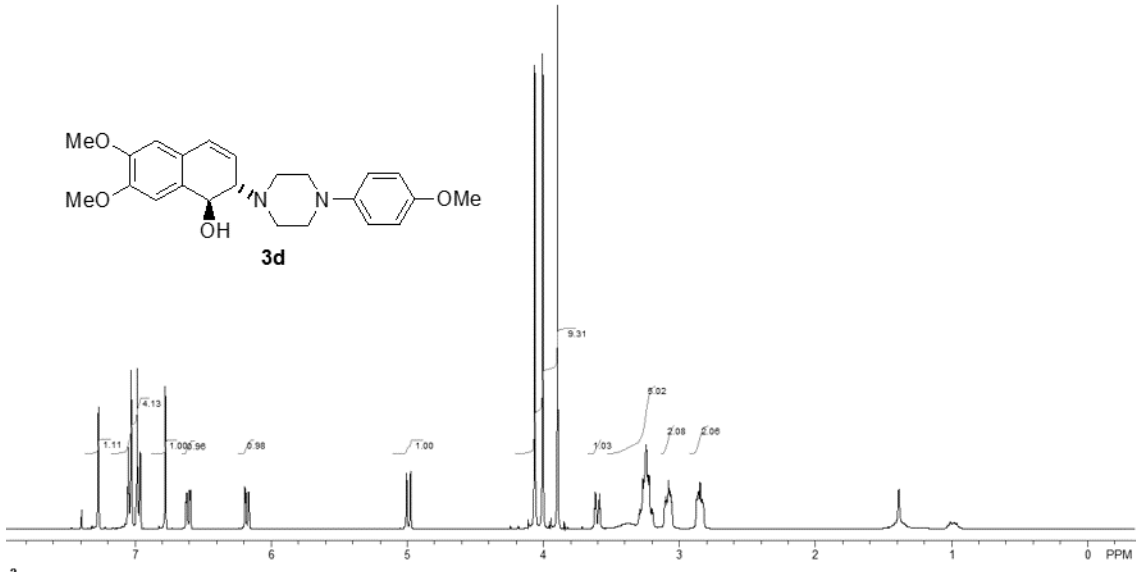

Figure S60. ^{1}H-NMR Spectra of Compound **3d**.

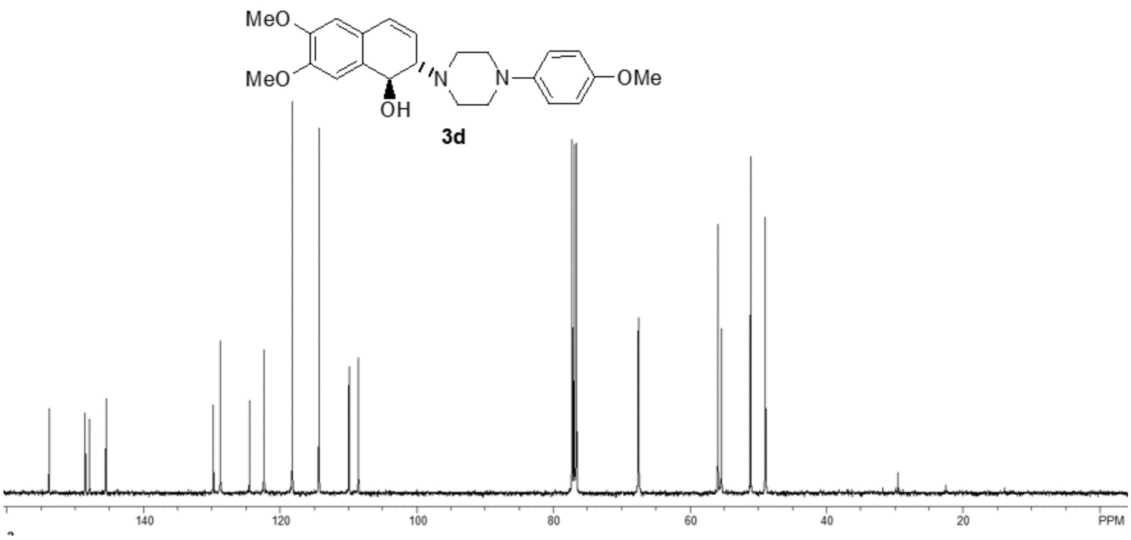

Figure S61. ^{13}C-NMR Spectra of Compound **3d**.

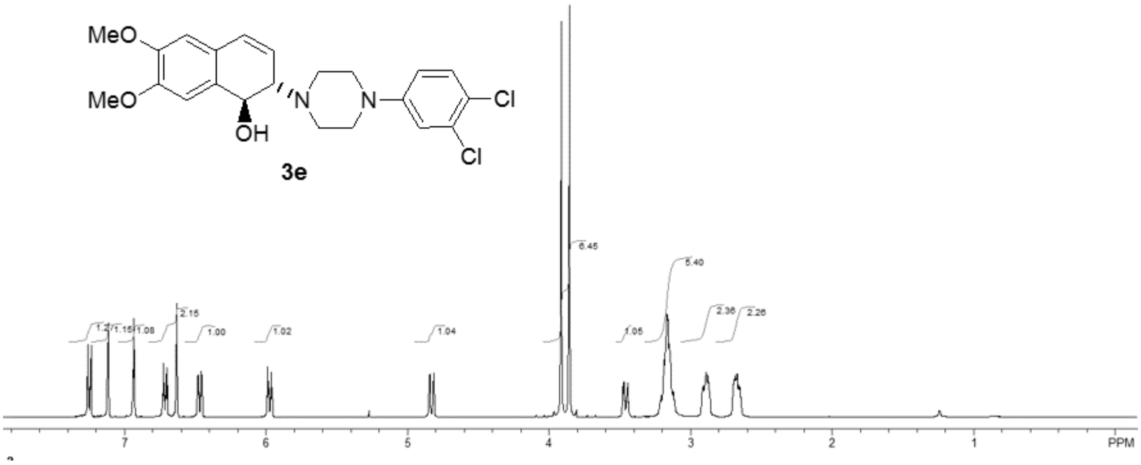

Figure S62. ^{1}H-NMR Spectra of Compound **3e**.

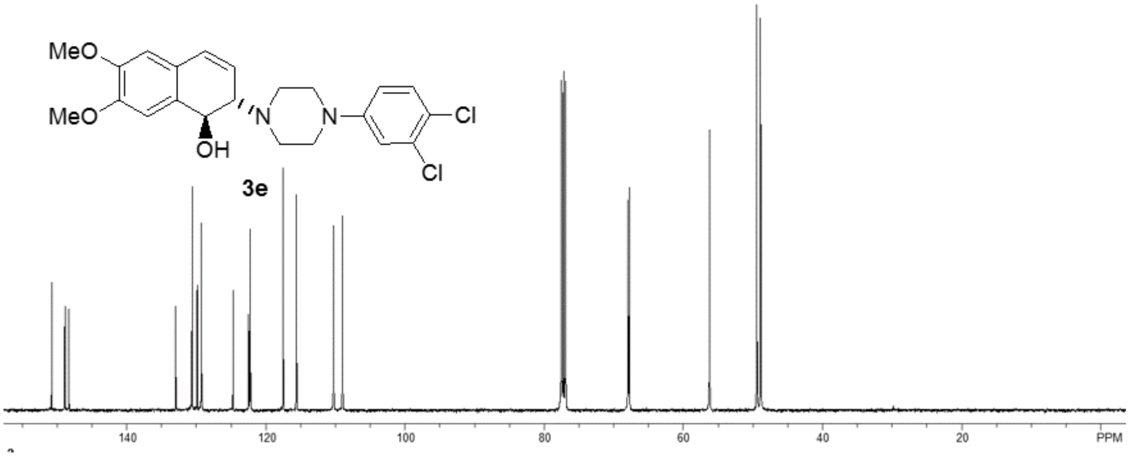

Figure S63. ^{13}C-NMR Spectra of Compound **3e**.

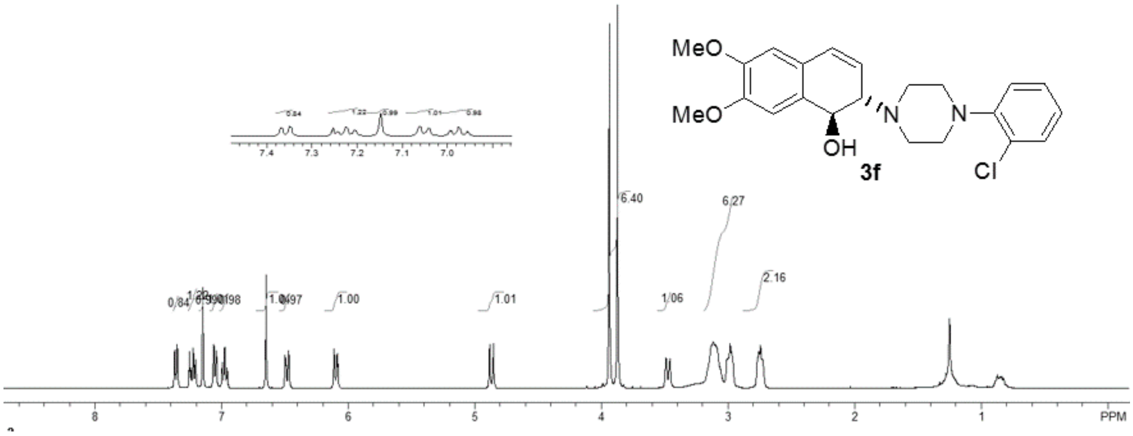

Figure S64. ^{1}H-NMR Spectra of Compound **3f**.

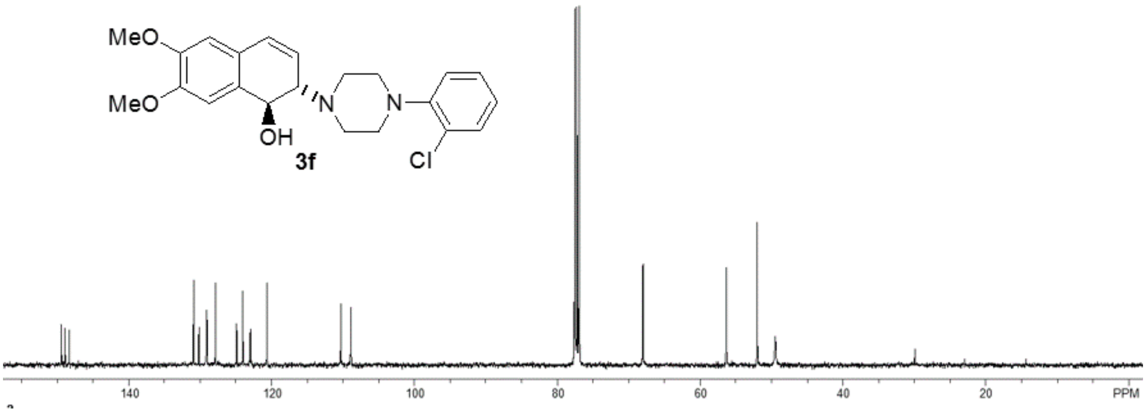

Figure S65. ^{13}C-NMR Spectra of Compound **3f**.

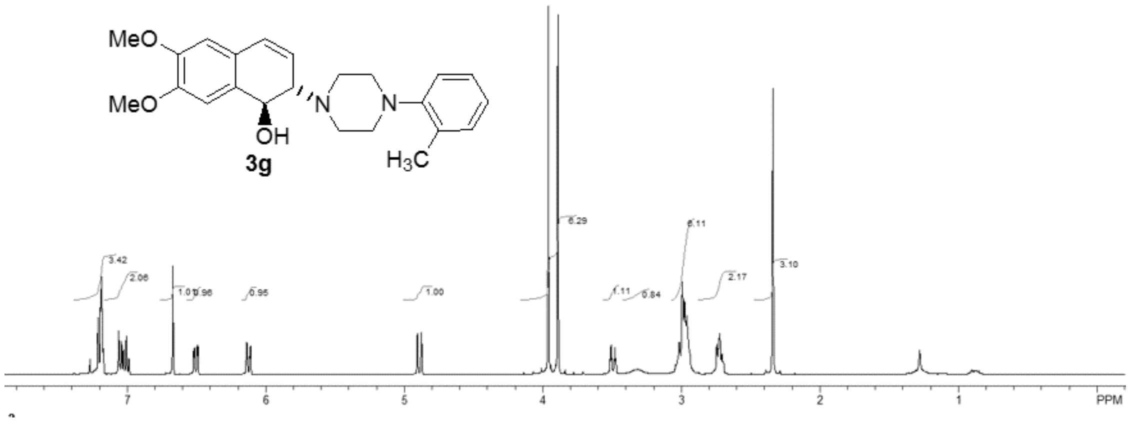

Figure S66. ^{1}H-NMR Spectra of Compound **3g**.

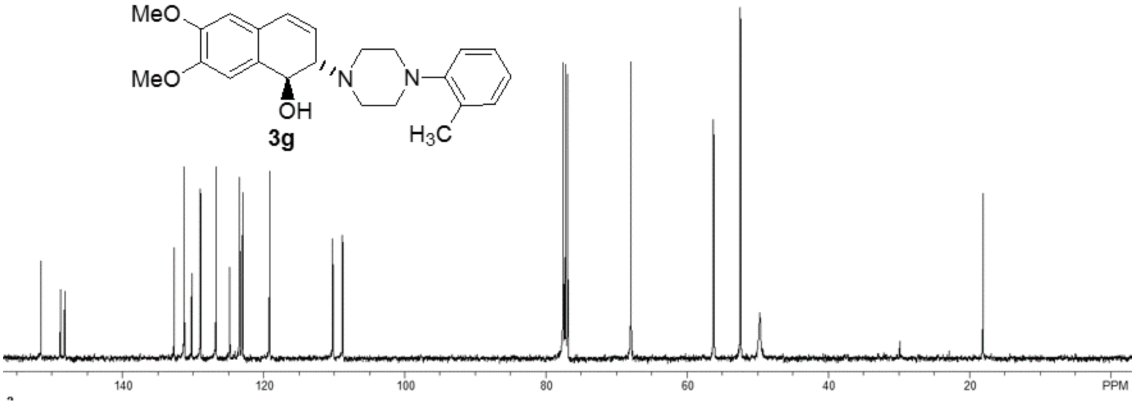

Figure S67. ^{13}C-NMR Spectra of Compound **3g**.

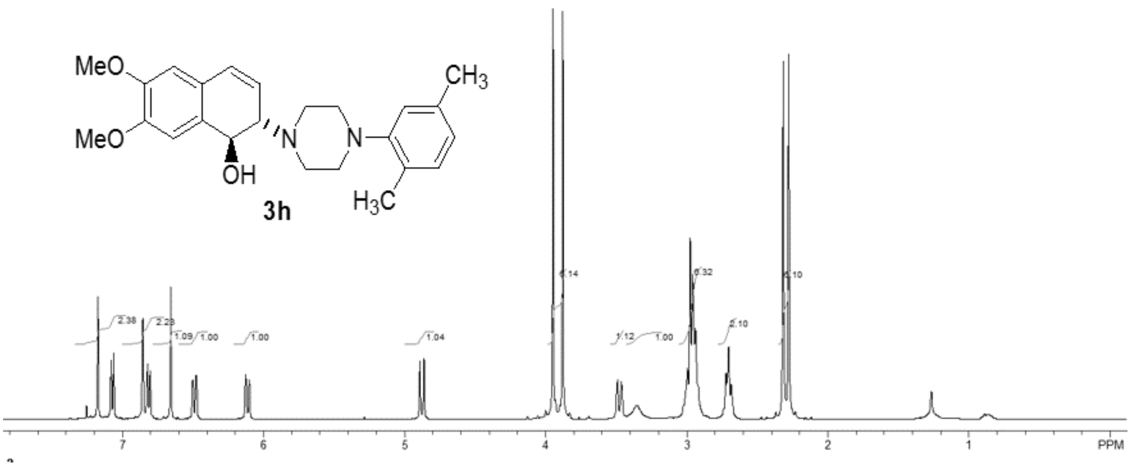

Figure S68. ^{1}H-NMR Spectra of Compound **3h**.

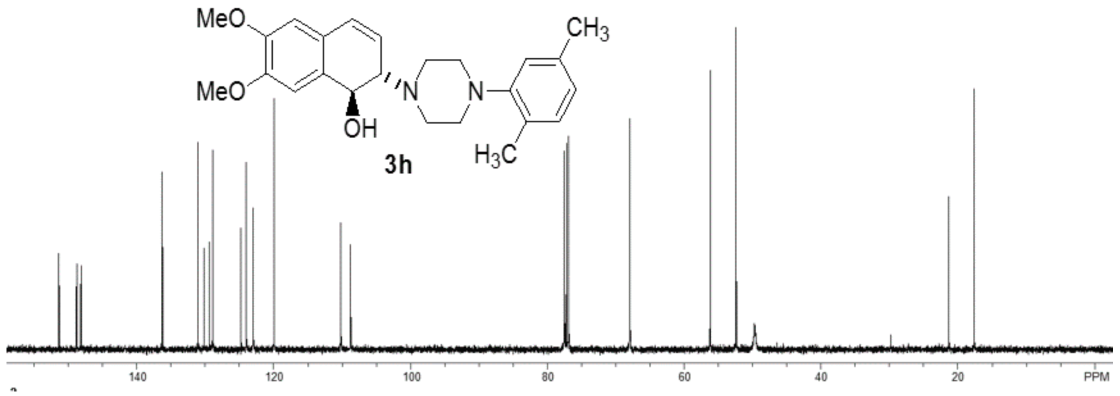

Figure S69. ^{13}C-NMR Spectra of Compound **3h**.

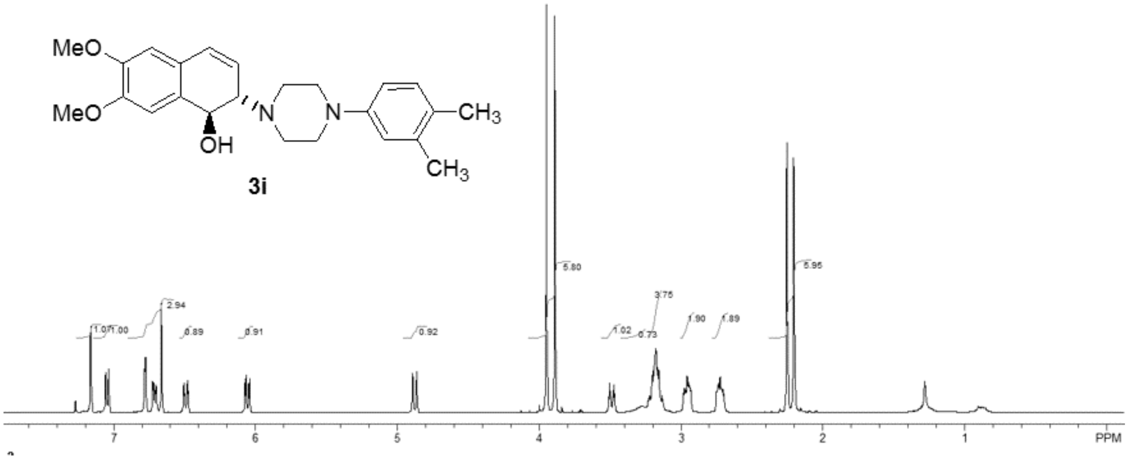

Figure S70. ^{1}H-NMR Spectra of Compound **3i**.

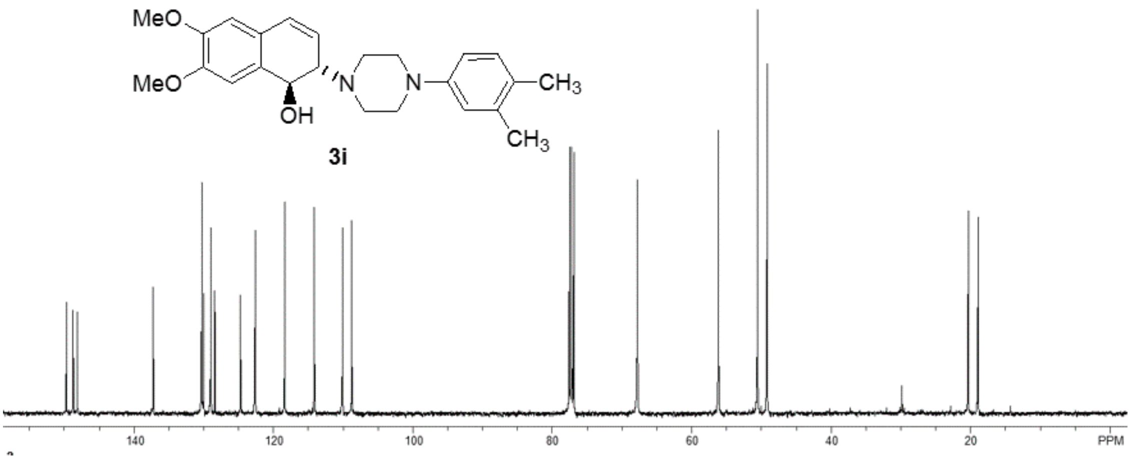

Figure S71. ^{13}C-NMR Spectra of Compound **3i**.

3. The Parts of Copies of HPLC of 2a, 2c, 2g–2h, 2i–2k, 2m–2o, 2q–2r, 2u–2x, and 3b

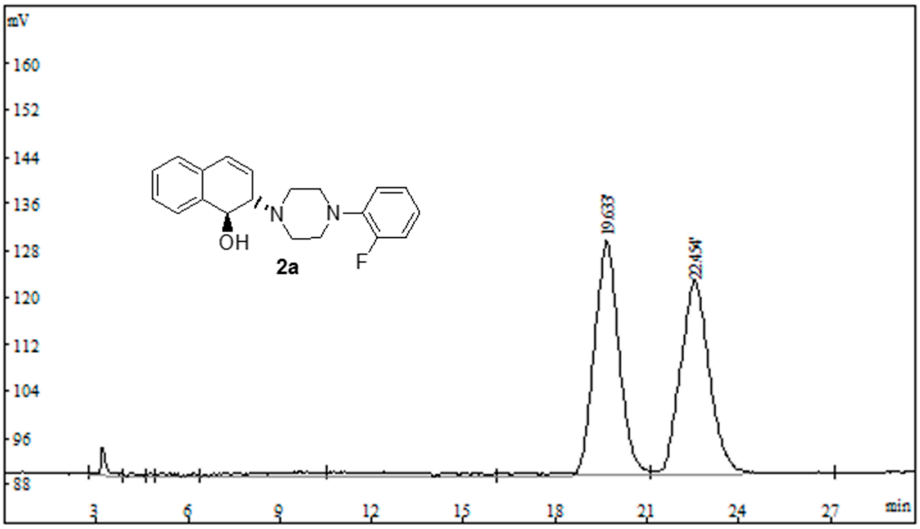

Peak No.	Time (min)	Area (mV × s)	Area (%)
1	19.633	2,286,790	45.72
2	22.454	2,202,149	44.03

Figure S72. HPLC trace for racemic-**2a**.

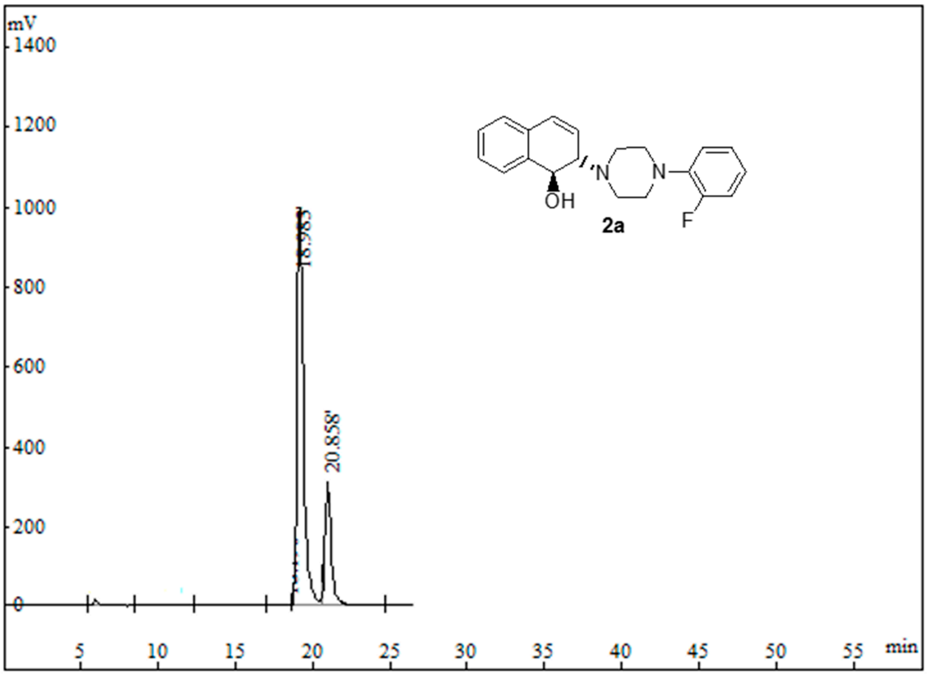

Peak No.	Time (min)	Area (mV × s)	Area (%)
1	18.983	30,127,222	75.35
2	20.858	8,637,715	21.6

Figure S73. HPLC trace for enantienriched-**2a** (*ee* = 54%).

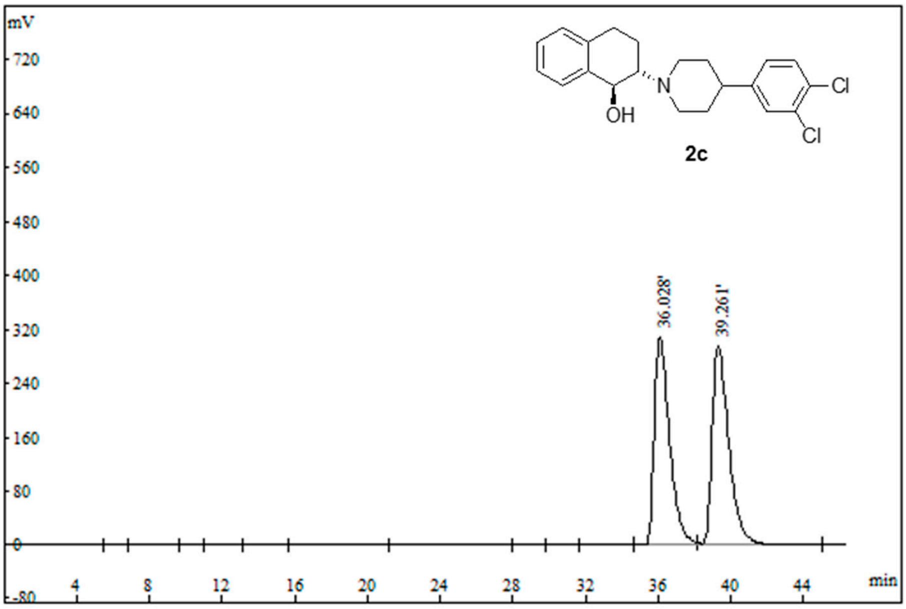

Peak No.	Time (min)	Area (mV × s)	Area (%)
1	36.028	18,296,867	48.97
2	39.261	18,777,477	50.25

Figure S74. HPLC trace for racemic-**2c**.

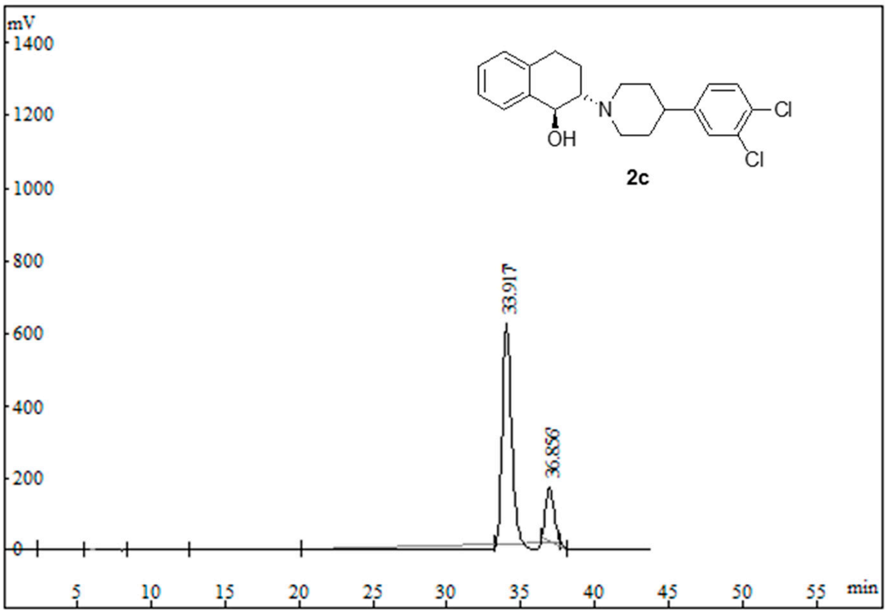

Peak No.	Time (min)	Area (mV × s)	Area (%)
1	33.917	31,687,830	81.72
2	36.856	5,873,271	15.15

Figure S75. HPLC trace for enantienriched-**2c** (*ee* = 67%).

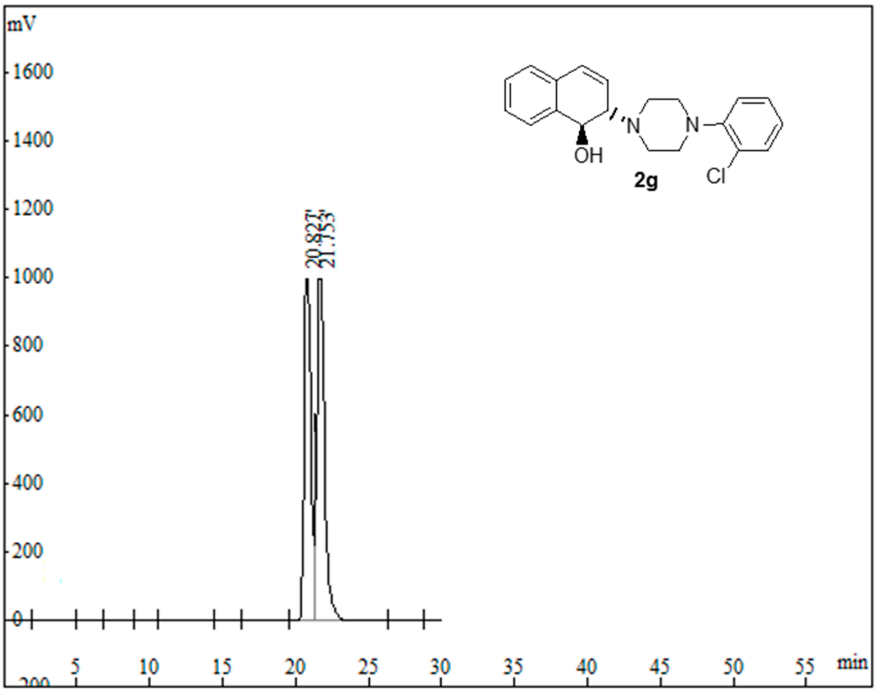

Peak No.	Time (min)	Area (mV × s)	Area (%)
1	20.827	36,409,520	50.68
2	21.753	34,616,080	48.19

Figure 76. HPLC trace for racemic-**2g**.

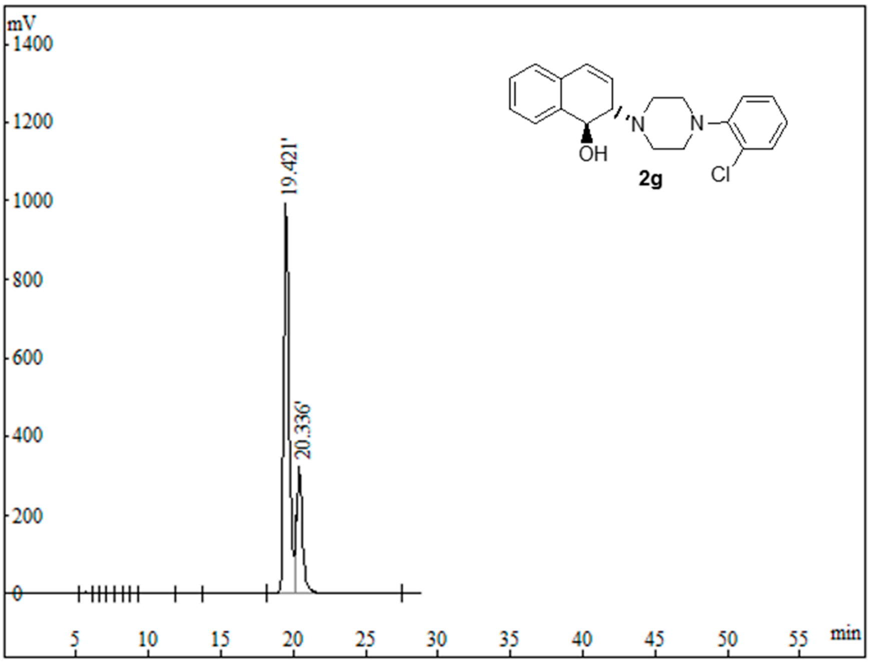

Peak No.	Time (min)	Area (mV × s)	Area (%)
1	19.421	26,983,894	72.82
2	20.336	8,912,826	24.05

Figure S77. HPLC trace for enantienriched-**2g** (*ee* = 50%).

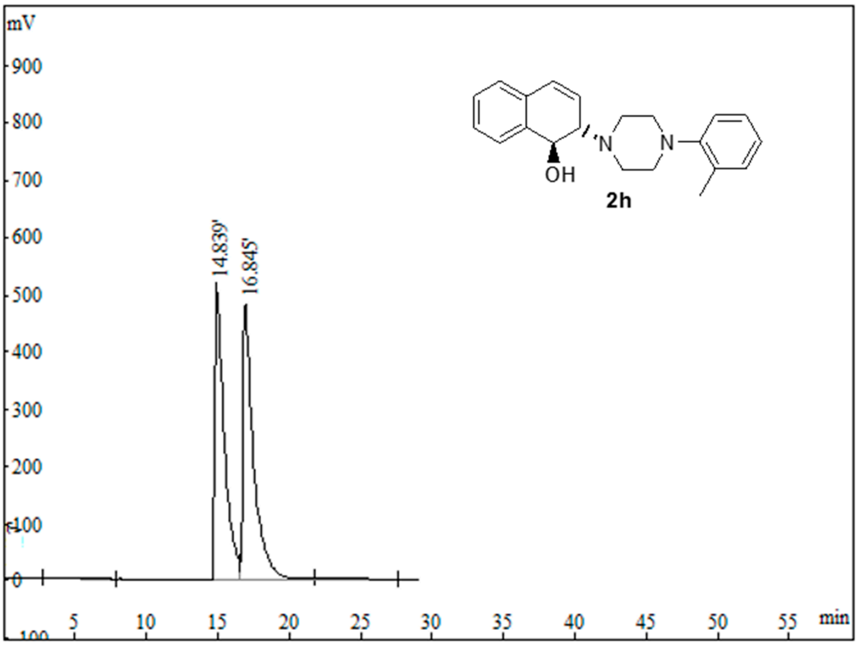

Peak No.	Time (min)	Area (mV × s)	Area (%)
1	14.839	24,075,317	47.04
2	16.845	25,723,102	50.26

Figure S78. HPLC trace for racemic-**2h**.

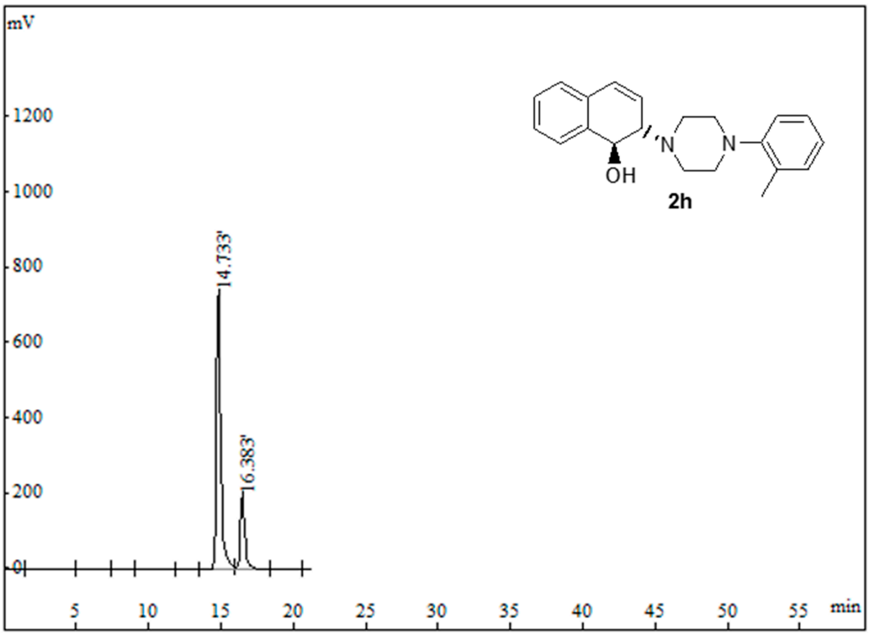

Peak No.	Time (min)	Area (mV × s)	Area (%)
1	14.733	16,082,419	76.49
2	16.383	4,764,761	22.66

Figure S79. HPLC trace for enantienriched-**2h** (*ee* = 54%).

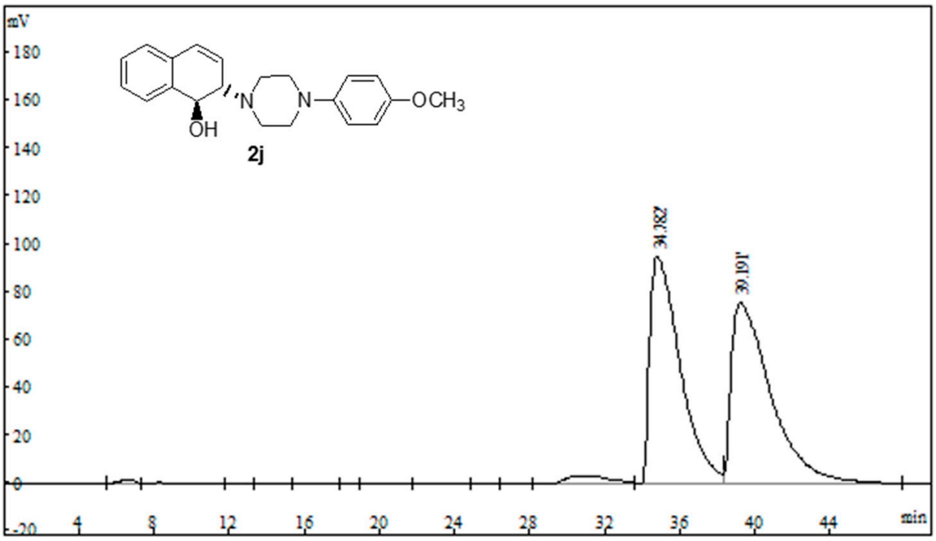

Peak No.	Time (min)	Area (mV × s)	Area (%)
1	34.782	10,868,088	47.55
2	39.191	11,020,779	48.21

Figure S80. HPLC trace for racemic-**2j**.

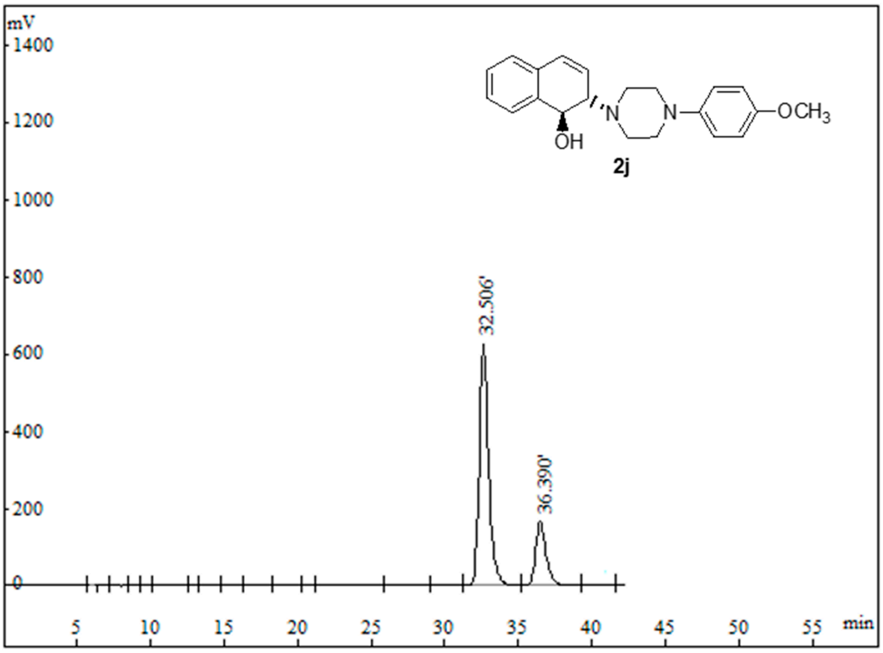

Peak No.	Time (min)	Area (mV × s)	Area (%)
1	32.506	27,526,003	71.29
2	36.390	8,157,145	21.13

Figure S81. HPLC trace for enantienriched-**2j** (*ee* = 54%).

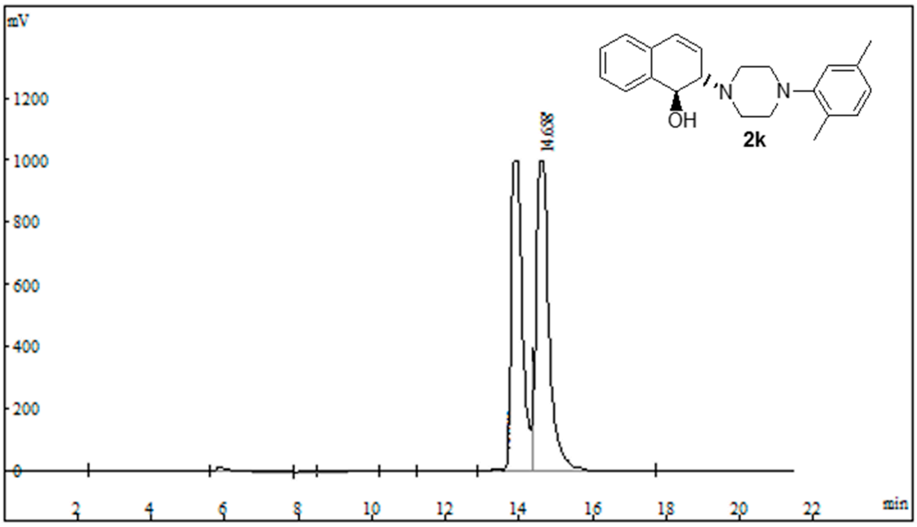

Peak No.	Time (min)	Area (mV × s)	Area (%)
1	13.658	22,953,626	46.48
2	14.658	24,009,168	48.61

Figure S82. HPLC trace for racemic-**2k**.

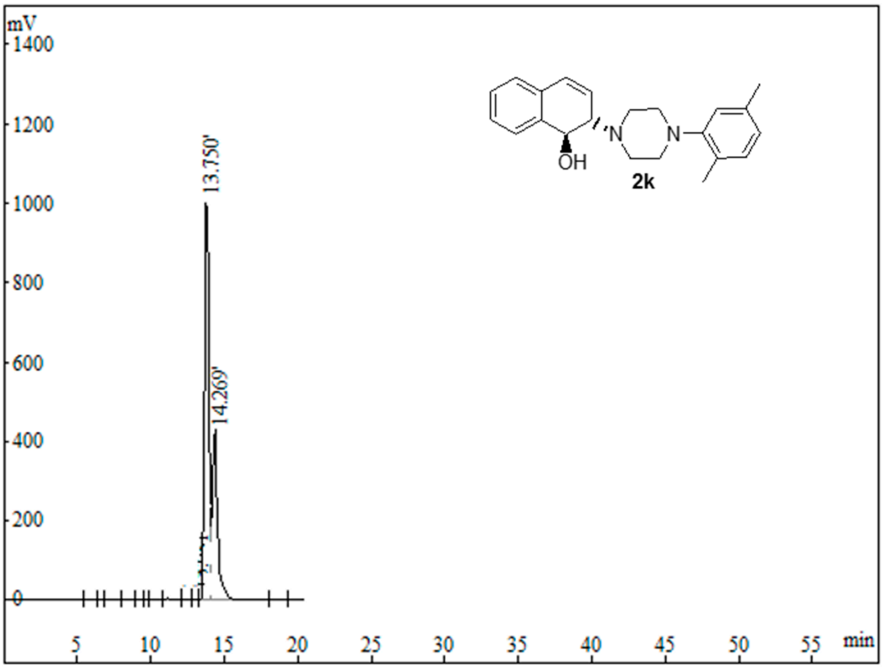

Peak No.	Time (min)	Area (mV × s)	Area (%)
1	13.750	22,130,480	66.58
2	14.269	10,407,103	31.31

Figure S83. HPLC trace for enantienriched-**2k** (*ee* = 36%).

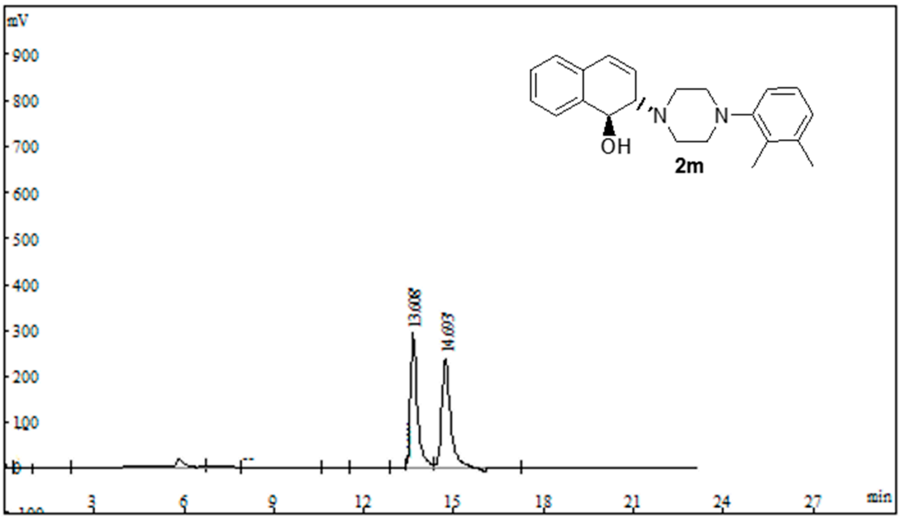

Peak No.	Time (min)	Area (mV × s)	Area (%)
1	13.608	4,981,223	41.64
2	14.693	5,073,084	42.4

Figure S84. HPLC trace for racemic-**2m**.

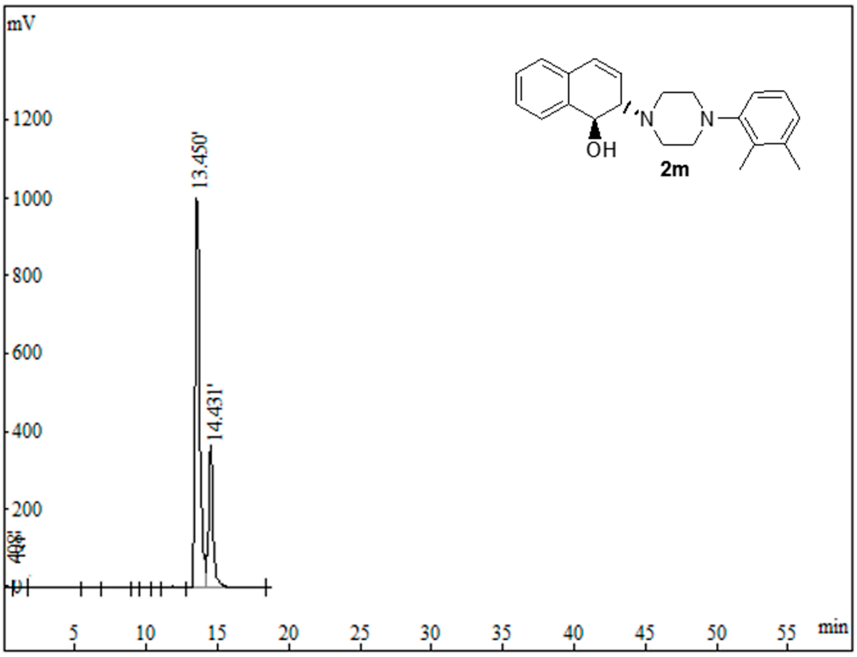

Peak No.	Time (min)	Area (mV × s)	Area (%)
1	13.450	23,271,726	72.72
2	14.431	8,359,838	26.12

Figure S85. HPLC trace for enantienriched-**2m** (*ee* = 47%).

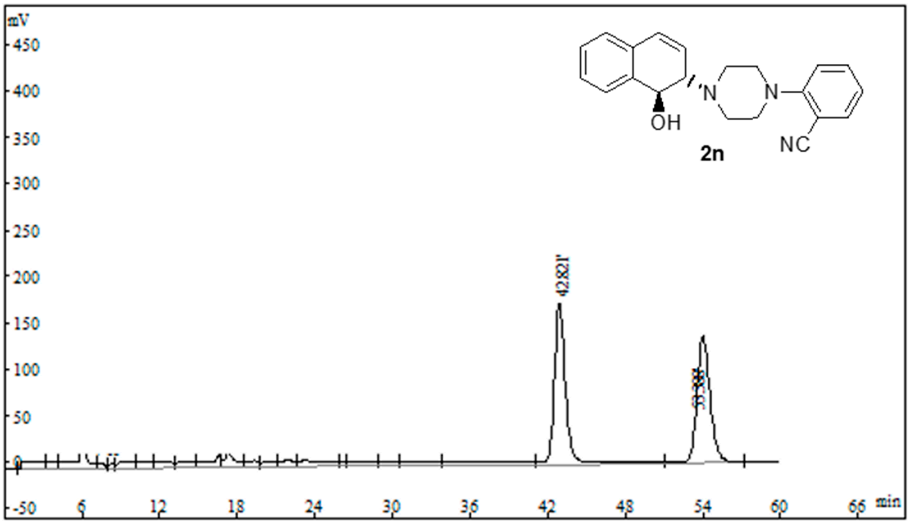

Peak No.	Time (min)	Area (mV × s)	Area (%)
1	42.821	10,633,778	31.2
2	53.388	9,727,495	28.54

Figure S86. HPLC trace for racemic-**2n**.

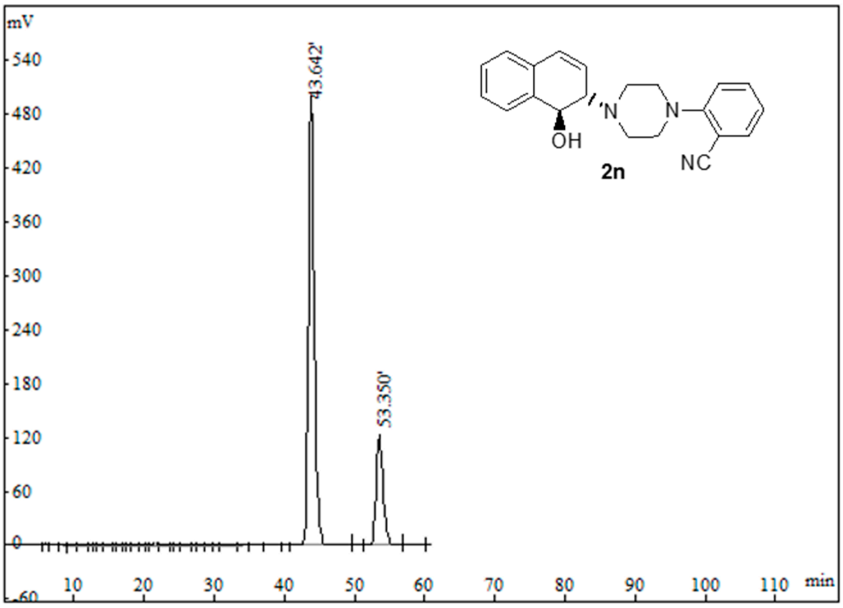

Peak No.	Time (min)	Area (mV × s)	Area (%)
1	43.642	28,898,995	72.56
2	53.350	8,575,389	21.53

Figure S87. HPLC trace for enantienriched-**2n** (*ee* = 54%).

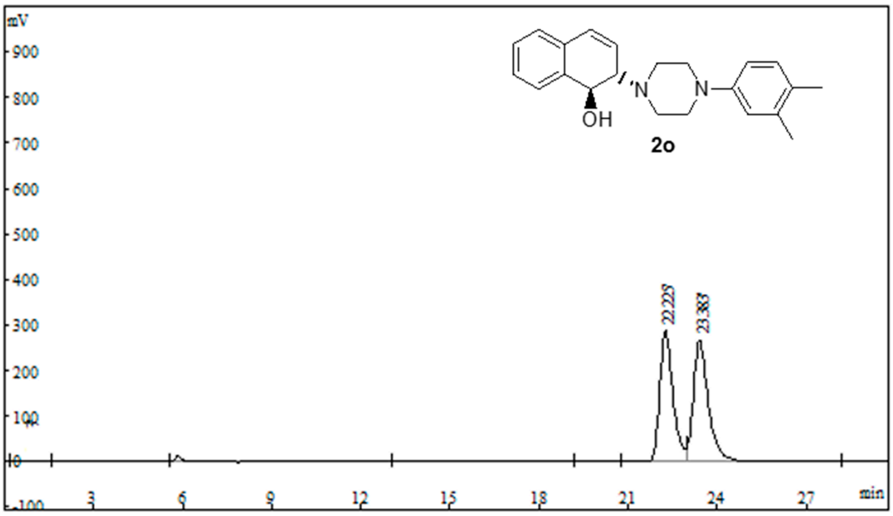

Peak No.	Time (min)	Area (mV × s)	Area (%)
1	22.225	9,151,633	47.66
2	23.383	9,403,859	48.98

Figure S88. HPLC trace for racemic-**2o**.

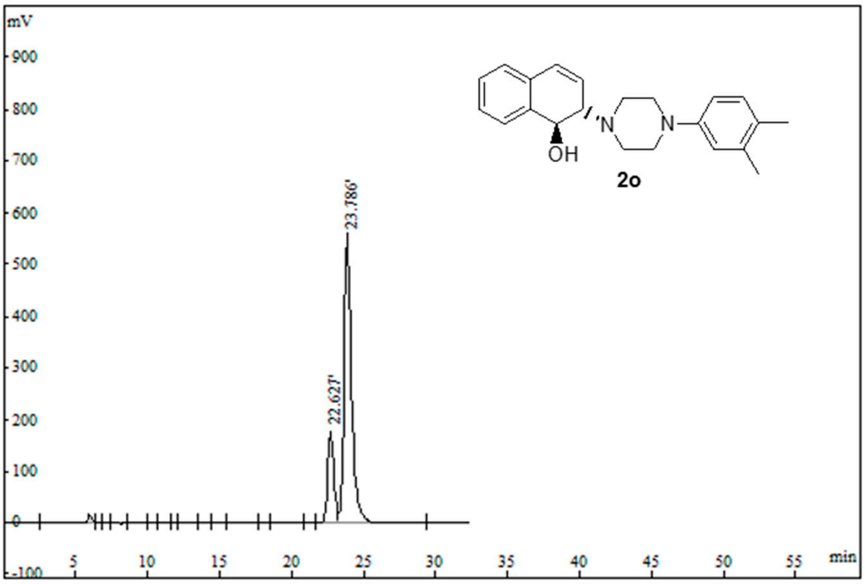

Peak No.	Time (min)	Area (mV × s)	Area (%)
1	22.627	5,422,855	19.78
2	23.786	20,200,648	73.69

Figure S89. HPLC trace for enantienriched-**2o** (*ee* = 58%).

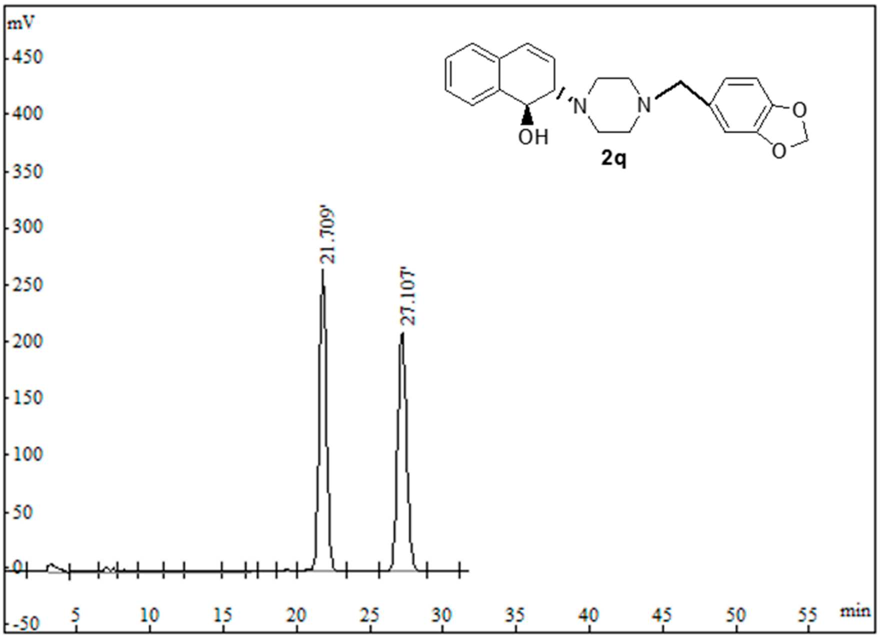

Peak No.	Time (min)	Area (mV × s)	Area (%)
1	21.709	9,459,897	43.64
2	27.107	9,146,611	42.2

Figure S90. HPLC trace for racemic-**2q**.

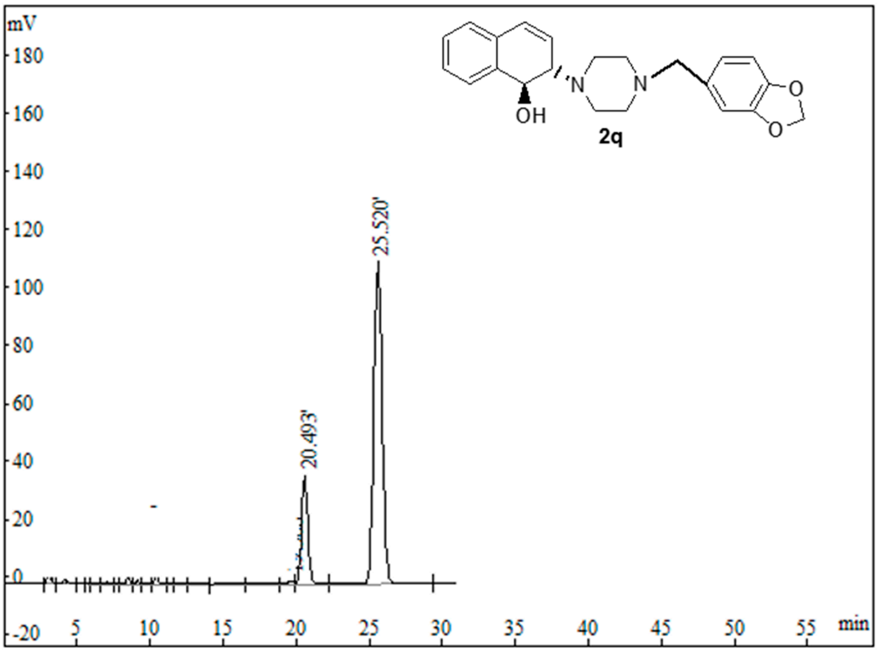

Peak No.	Time (min)	Area (mV × s)	Area (%)
1	20.493	1,191,507	18.73
2	25.520	4,343,328	68.27

Figure S91. HPLC trace for enantienriched-**2q** (*ee* = 57%).

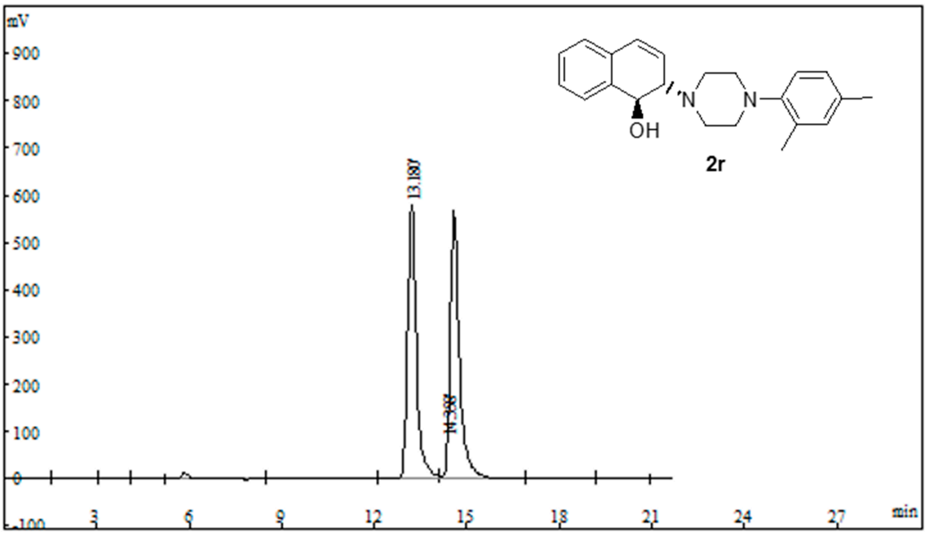

Peak No.	Time (min)	Area (mV × s)	Area (%)
1	13.180	11,899,451	47.59
2	14.358	12,051,429	48.19

Figure S92. HPLC trace for racemic-**2r**.

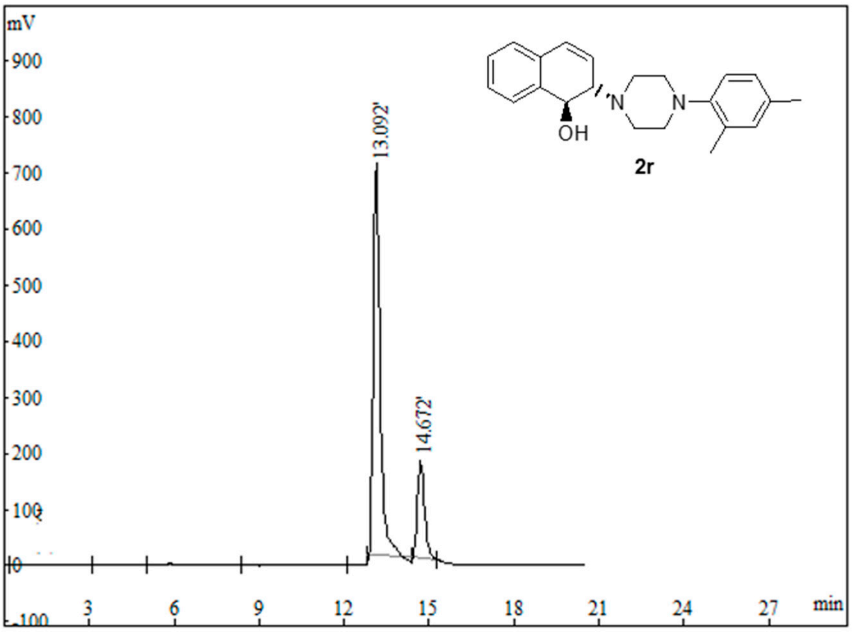

Peak No.	Time (min)	Area (mV × s)	Area (%)
1	13.092	11,836,752	78.49
2	14.672	3,075,319	20.39

Figure S93. HPLC trace for enantienriched-**2r** (*ee* = 59%).

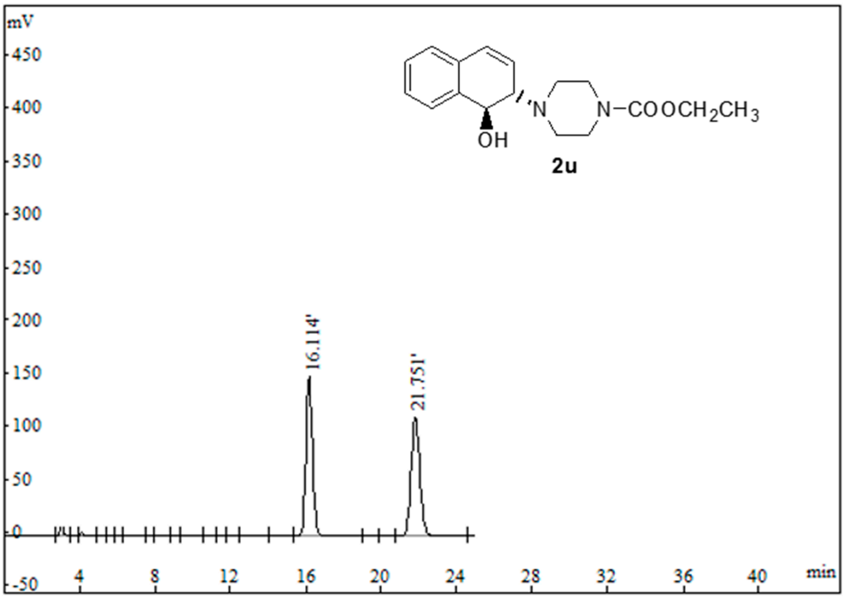

Peak No.	Time (min)	Area (mV × s)	Area (%)
1	16.114	3,729,452	45.83
2	21.751	3,654,966	44.91

Figure S94. HPLC trace for racemic-**2u**.

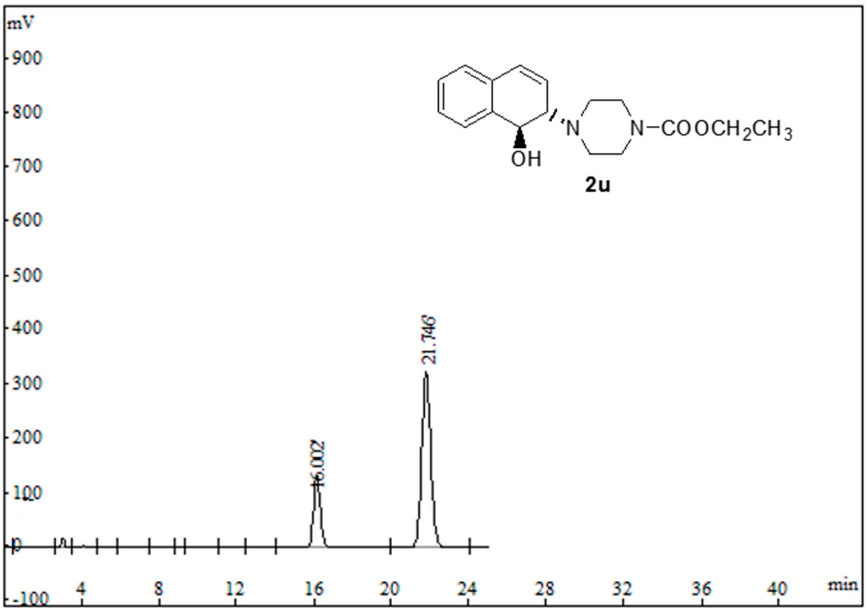

Peak No.	Time (min)	Area (mV × s)	Area (%)
1	16.002	3,519,111	23.41
2	21.746	10,675,825	71.02

Figure S95. HPLC trace for enantienriched-**2u** (*ee* = 51%).

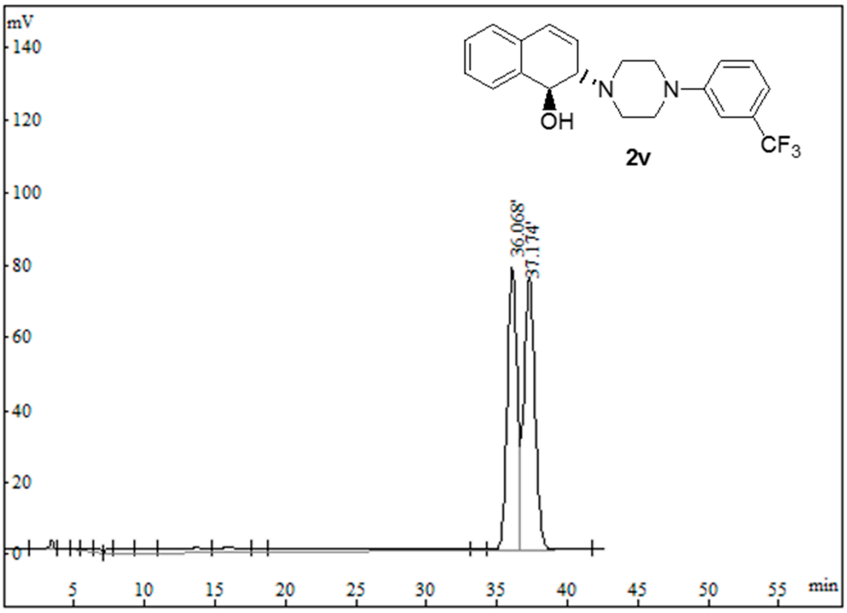

Peak No.	Time (min)	Area (mV × s)	Area (%)
1	36.068	3,908,792	37.32
2	37.174	4,108,356	39.23

Figure S96. HPLC trace for racemic-**2v**.

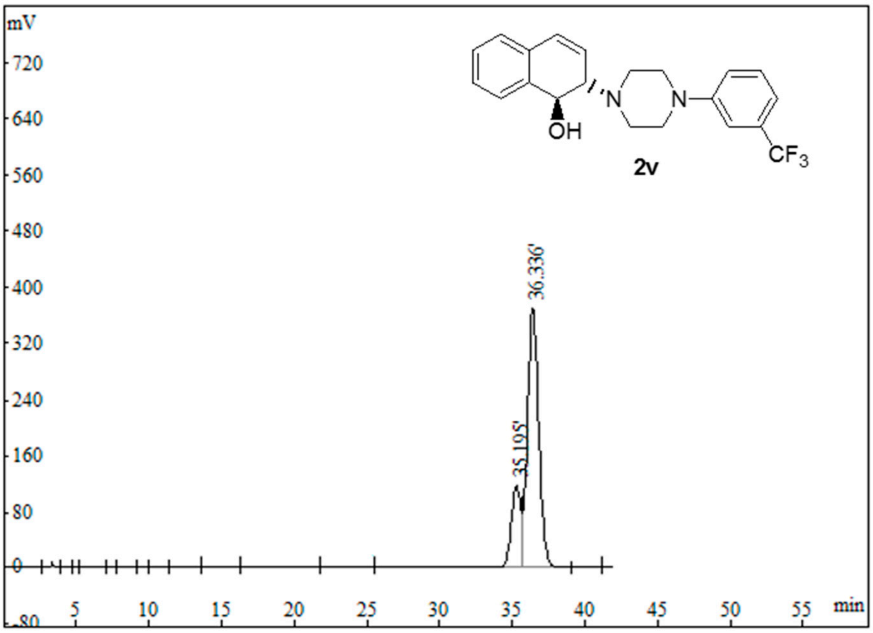

Peak No.	Time (min)	Area (mV × s)	Area (%)
1	35.195	5,825,376	22.11
2	36.336	19,589,714	74.34

Figure S97. HPLC trace for enantienriched-**2v** (*ee* = 54%).

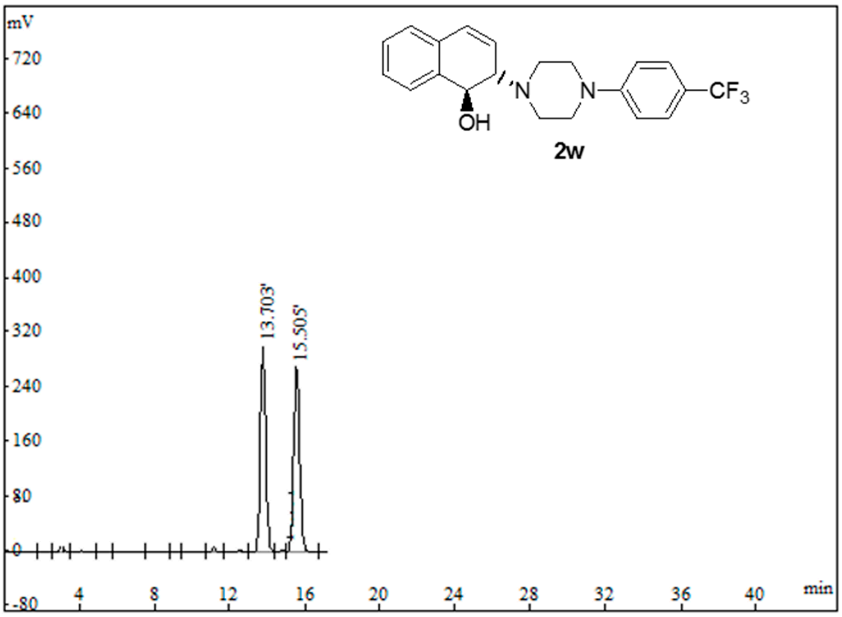

Peak No.	Time (min)	Area (mV × s)	Area (%)
1	13.703	6,074,666	47.06
2	15.505	6,107,114	47.32

Figure S98. HPLC trace for racemic-**2w**.

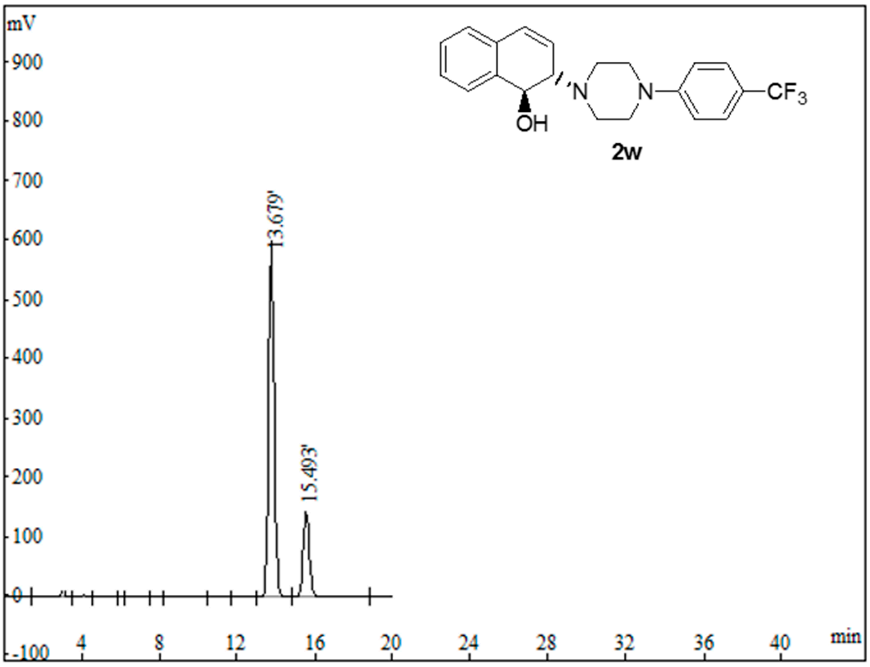

Peak No.	Time (min)	Area (mV × s)	Area (%)
1	13.679	11,962,356	75.53
2	15.493	3,336,627	21.07

Figure S99. HPLC trace for enantienriched-**2w** (*ee* = 56%).

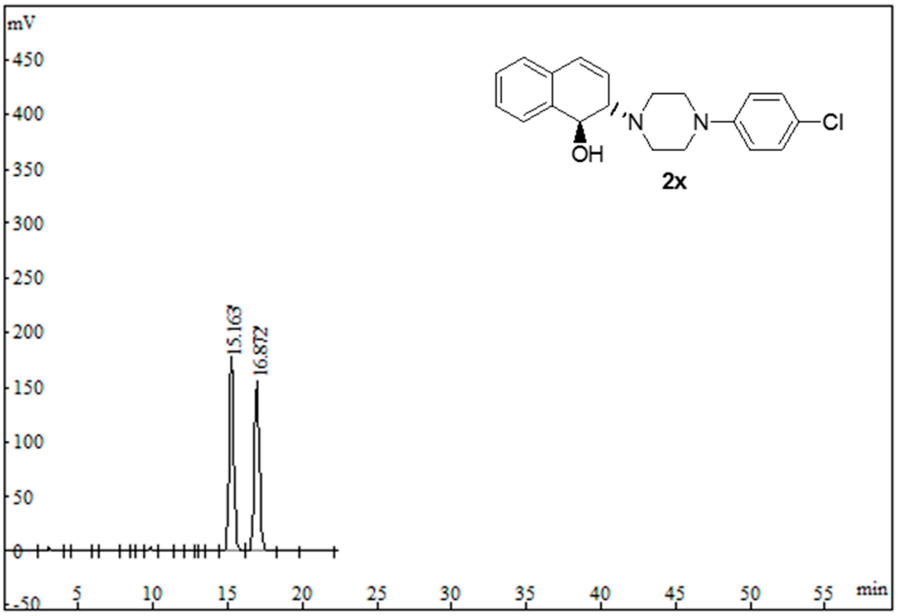

Peak No.	Time (min)	Area (mV × s)	Area (%)
1	15.163	3,929,200	45.61
2	16.872	3,797,234	44.08

Figure S100. HPLC trace for racemic-**2x**.

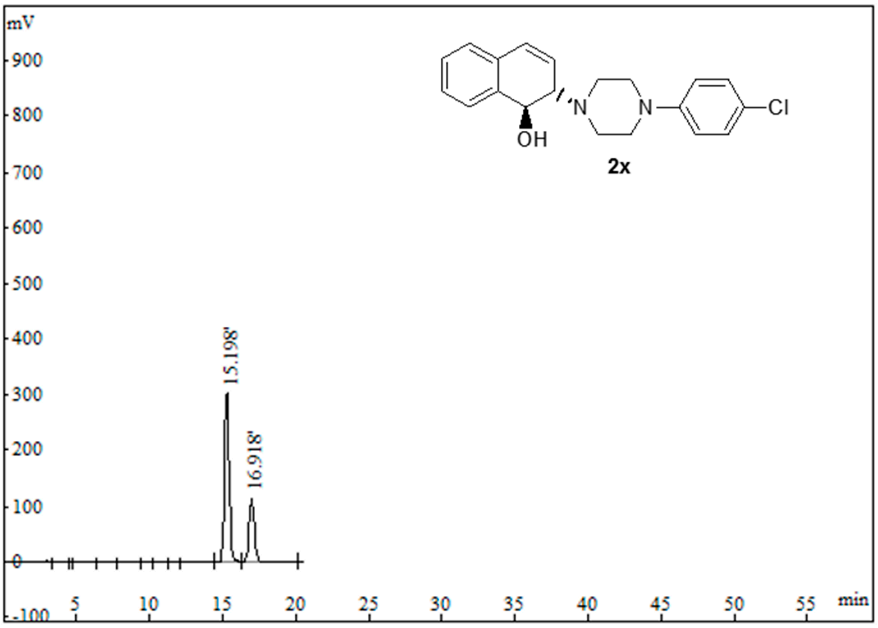

Peak No.	Time (min)	Area (mV × s)	Area (%)
1	15.198	6,978,256	63.59
2	16.918	3,077,079	28.04

Figure S101. HPLC trace for enantienriched-**2x** (*ee* = 39%).

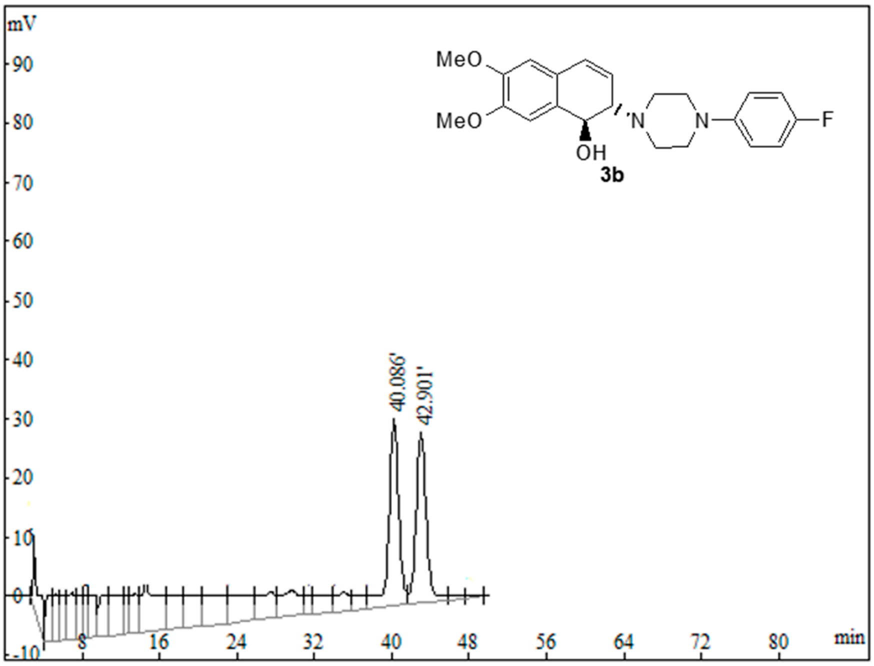

Peak No.	Time (min)	Area (mV × s)	Area (%)
1	40.086	2,333,980	15.26
2	42.901	2,166,930	14.16

Figure S102. HPLC trace for racemic-**3b**.

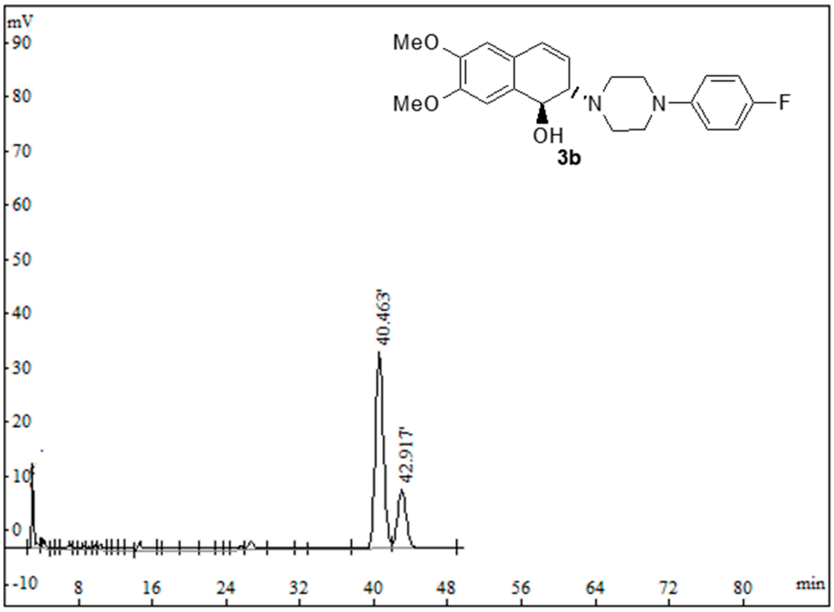

Peak No.	Time (min)	Area (mV × s)	Area (%)
1	40.463	2,253,452	50.47
2	42.917	779,103	17.45

Figure S103. HPLC trace for enantienriched-**3b** (*ee* = 49%).
